# Being HIV positive and staying on antiretroviral therapy in Africa: A qualitative systematic review and theoretical model

**DOI:** 10.1371/journal.pone.0210408

**Published:** 2019-01-10

**Authors:** Ingrid Eshun-Wilson, Anke Rohwer, Lynn Hendricks, Sandy Oliver, Paul Garner

**Affiliations:** 1 Department of Global Health, Faculty of Medicine and Health Sciences, Stellenbosch University, Cape Town, South Africa; 2 Department of Medicine, University of California San Francisco, San Francisco, California, United States of America; 3 UCL Institute of Education, University College London, London, United Kingdom; 4 Africa Centre for Evidence, University of Johannesburg, Johannesburg, South Africa; 5 Centre for Evidence Synthesis in Global Health, Liverpool School of Tropical Medicine, Liverpool, United Kingdom; Médecins Sans Frontières (MSF), SOUTH AFRICA

## Abstract

**Background:**

Adherence to antiretroviral therapy (ART) and long-term uninterrupted engagement in HIV care is difficult for HIV-positive people, and randomized trials of specific techniques to promote adherence often show small or negligible effects. Understanding what influences decision-making in HIV-positive people in Africa may help researchers and policy makers in the development of broader, more effective interventions and policies.

**Methods:**

We used thematic synthesis and a grounded theory approach to generate a detailed narrative and theoretical model reflecting life with HIV in Africa, and how this influences ART adherence and engagement decisions. We included qualitative primary studies that explored perspectives, perceptions and experiences of HIV-positive people, caregivers and healthcare service providers. We searched databases from 1 January 2013 to 9 December 2016, screened all studies, and selected those for inclusion using purposeful sampling methods. Included studies were coded with Atlas.ti, and we assessed methodological quality across five domains.

**Results:**

We included 59 studies from Africa in the synthesis. Nine themes emerged which we grouped under three main headings. First, people who are HIV-positive live in a complicated world where they must navigate the challenges presented by poverty, competing priorities, unpredictable life events, social identity, gender norms, stigma, and medical pluralism—these influences can make initiating and maintaining ART difficult. Second, the health system is generally seen as punishing and uninviting and this can drive HIV-positive people out of care. Third, long-term engagement and adherence requires adaptation and incorporation of ART into daily life, a process which is facilitated by: inherent self-efficacy, social responsibilities, previous HIV-related illnesses and emotional, practical or financial support. These factors together can lead to a “tipping point”, a point in time when patients choose to either engage or disengage from care. HIV-positive people may cycle in and out of these care states in response to fluctuations in influences over time.

**Conclusion:**

This analysis provides a practical theory, arising from thematic synthesis of research, to help understand the dynamics of adherence to ART and engagement in HIV care. This can contribute to the design of service delivery approaches, and informed thinking and action on the part of policy makers, providers, and society: to understand what it is to be HIV-positive in Africa and how attitudes and the health service need to shift to help those with HIV lead ‘normal’ lives.

## Introduction

Initiating and maintaining HIV-positive people on antiretroviral therapy (ART) over the long course of HIV infection represents a growing challenge, as initiatives such ‘test and treat’ [1] and ‘rapid-ART’ initiation [2] expand ART provision globally. Africa carries the largest burden of HIV infections, with approximately 26 million HIV-positive people living in the region, and just over 50% of these accessing ART in 2017 [3]. Reports suggest that—among those on ART—retention in care [4] and viral suppression continues to lag behind targets [5] and this has led to substantial investments in interventions to improve retention and adherence [6]. Meta-analyses demonstrate that single interventions such as mobile phone reminders have modest effects on improving adherence to ART, that there is marked heterogeneity of effectiveness across trials, that effects may wane over time, and that even community-based initiatives show little impact overall [7–9]. Several ‘patient-centered’ interventions are currently being developed and implemented [6], however few offer highly effective solutions, particularly for vulnerable groups such as women and children, key populations, men, and young adults.

The lack of effectiveness of adherence and retention interventions has led to a shift from a technocratic approach to sociopolitical resulting in a proliferation of studies evaluating barriers and facilitators to ART adherence and engagement in care. The current literature however does not clearly reflect the pervasive set of challenges that HIV-positive people face, the interdependence of all these influences and how these impact on the HIV care cascade. Our evaluation of published systematic reviews on barriers and facilitators to ART adherence and engagement identified that many syntheses are either outdated, focused on specific sub-populations, generate simplified lists of barriers and facilitators, or evaluate primarily individual components of the HIV care cascade, such as linkage or retention [10–26]. And, although there are several existing health behavior theories which can be applied to HIV care [27, 28], many these, including the Information, Motivation, Behavioral Skills Model, Theories of Reasoned Action and Planned Behavior, the Transtheoretical Model, and Social Cognitive Theory focus primarily on an individual’s ability to perform a health behavior with comparatively little focus on the role of society and the environment. Frameworks such as the social ecological framework [28, 29] and the behavior change wheel proposed Michie et al [30], incorporate the role of society and the environment at a greater level, and are useful for categorizing and mapping barriers and facilitators directly to interventions, but do not capture the dynamic nature of influences and how these change over time for individuals.

We used thematic synthesis and a grounded theory approach to conduct a systematic review and meta-synthesis of qualitative research conducted across Africa and present a broad perspective of the fundamental drivers of ART adherence and engagement behaviors, with the intention of generating a generalizable theory to help practitioners, policy makers and researchers consider what influences may impact on responses to HIV care at all levels of the cascade. Our aim is to inform an understanding of ‘why people do what they do’ and assist with future development of truly patient-centered health services and policies for HIV-positive people in Africa.

## Methods

We defined our methods in advance with a protocol agreed between authors [31], differences between the protocol and final methods are detailed in S1 Appendix.

### Criteria for considering studies for inclusion

We considered qualitative studies that used ethnographies, process evaluations, case studies, and mixed-methods and used data collection techniques including observations, interviews, focus groups, and document analyses to collect qualitative data; and thematic analyses, narrative analyses, and presentations of findings to analyze qualitative data. Studies that used qualitative data collection methods but not qualitative data analysis methods were ineligible. We restricted studies to those conducted in low-and-middle-income countries (LMICs). We included studies that explored our phenomenon of interest, specifically—perspectives, perceptions, and experiences of HIV-positive people, caregivers, and providers that influenced linkage, retention, and adherence to ART. We included studies with participants who were: HIV-positive children, adolescents and adults, healthcare workers, traditional healers who provided services to HIV-positive people, and caregivers of HIV-positive people. We excluded studies conducted in women receiving short-term ART prophylaxis solely for prevention of mother to child transmission and not for their own health.

### Search methods for identifying studies

We searched Medline, Embase, CINAHL, PsychInfo, LILACS, Global Health Library (date of last search 4 December 2016) and the Proquest Dissertation and Thesis database (9 December 2016). We limited our search to studies published from 1 January 2013 to include the most recent literature. Our search strategy included terms related to HIV, retention in care, adherence, linkage, LMICs and qualitative data collection and analysis methods. The full search strategies for each database are reported in S2 Appendix.

### Selection of studies

After de-duplication, two review authors (IEW and AR or LH) independently screened titles and abstracts to identify potentially relevant studies using Covidence [32], resolving discrepancies through discussions and consultation of a third author. Two authors (IEW and AR or LH) independently screened all full texts to identify eligible studies and one author (IEW) extracted data on the participants, setting, phenomenon of interest and richness of data. Discrepancies in eligibility and extracted data were discussed with a third author.

Once all studies which were eligible for inclusion were identified we used three purposeful sampling techniques to select the studies for final inclusion and data extraction [33–35]. First, we used intensity sampling to select rich examples of the phenomenon of interest. We assessed richness of included studies and classified studies accordingly as ‘thin’ or ‘thick’. ‘Thick’ was defined according to the depth of analysis reflected in the primary study authors interpretation of findings, including any of the following features: 1) the extent to which the authors transformed/analyzed their findings (beyond lists of barriers and facilitators), 2) insight into participants perspectives was demonstrated, 3) richness and complexity had been portrayed (variation explained, meanings illuminated), and 4) theoretical or conceptual development. Second, to ensure that all population groups were represented we expanded the sample by further selecting eligible studies conducted in additional population groups of interest. Here we included all additional studies—irrespective of whether they were ‘thick or thin’—conducted in children and adolescents, pregnant women, key populations (sex-workers, people who inject drugs, men who have sex with men) and vulnerable populations (people with disabilities, refugees). Finally, we restricted our sample to studies conducted in Africa as it became evident that our search yielded few ‘thick’ studies from other LMIC settings.

### Data extraction and synthesis

Two authors (IEW and AR or LH) independently coded studies inductively, identifying new codes using a grounded theory approach. We conducted In Vivo coding and generated memos capturing emerging themes using Atlas.ti software [36]. We discussed the codes of each study, resolved discrepancies and added new codes. We coded the authors results, discussion and conclusions from the primary studies. We used thematic synthesis to identify themes and subthemes. This was an iterative process through frequent weekly or biweekly small group meetings (IEW, AR and LH), three intensive three day team sessions (IEW, AR, LH, and PG) and monthly large group meetings (IEW, AR, LH, PG, and SO). We repeatedly refined our conceptual model and codes according to the preliminary themes. We then re-evaluated the remaining eligible studies and searched for confirming and disconfirming cases and selected additional studies for data extraction [33]. Here we selected those studies that expanded on existing themes, added illustrative examples, added data for new themes or contradicted existing themes. Essentially, further studies were included if they enriched our data further, either by supporting previous findings where there was limited data, broadening our perspective of preliminary findings or through introduction of new perspectives. We excluded studies where no new information was introduced or there was no need for further supporting information in our synthesis regarding the codes and themes identified in the primary study. Two authors (IEW and AR) independently assessed these studies for inclusion, then proceeded with independent coding of included studies and discussed each study to resolve discrepancies. The additional data helped expand on and refine preliminary themes. This process was continued until no new themes emerged and data saturation had been reached. The final codes used to refine themes are presented in S3 Appendix. We adhered to the ENTREQ guideline for transparency in reporting of synthesis of qualitative research (S4 Appendix).

### Methodological quality assessment

We assessed quality of included studies using a modified version of the tool developed by the EPPI-center [37] (S1 Table). Two authors (IEW and LH) independently assessed included studies in terms of (1) rigor in sampling: the sampling strategy was appropriate to the questions posed in the study; attempts were made to obtain a diverse sample of the population in question; characteristics of the sample critical to the understanding of the study context and findings were presented, (2) rigor in data collection: data collection tools were piloted; data collection was comprehensive, flexible and/or sensitive enough to provide a complete and/or vivid and rich description of people’s perspectives and experiences; steps were taken to ensure that all participants were able and willing to contribute, (3) rigor in analysis: data analysis methods were systematic; diversity in perspective was explored; the analysis was balanced in the extent to which it was guided by preconceptions or by the data; the analysis sought to rule out alternative explanations for findings, (4) findings of the study grounded in/ supported by the data: enough data are presented to show how the authors arrived at their findings; the data presented fit the interpretation/support claims about patterns in data; the data presented illuminate/illustrate the findings; quotes are numbered or otherwise identified, and (5) the breadth and depth of study findings: (considering ‘breadth’ as the extent of description and ‘depth’ as the extent to which data has been transformed/analysed); a range of issues are covered; the perspectives of participants are fully explored in terms of breadth (contrast of two or more perspectives) and depth (insight into a single perspective); richness and complexity has been portrayed (e.g. variation explained, meanings illuminated); there has been theoretical/conceptual development. These domains were assessed as: (1) Yes, a fairly thorough attempt was made, (2) Yes, several steps were taken, (3) Yes, a few steps were taken, and (4) No, not at all/Not stated/Can’t tell. Discrepancies were resolved through discussion or consultation of a third author (PG or SO).

### Reflexivity

The author team had a variety of research skills, which included both primary research and evidence synthesis methods for quantitative and qualitative study designs. All had experience working or conducting research in sub-Saharan Africa and fields of study were diverse including medicine, HIV management (IEW), infectious diseases (PG), nursing and midwifery (AR), and social science (LH and SO). Due to the subjective nature of qualitative synthesis we had regular discussion meetings to reflect on personal biases or judgements throughout the process of the synthesis. These discussions allowed us to reflect on emerging themes through different perspectives and construct meaning in the current synthesis.

## Results

### Results of the search

**Fig 1:**
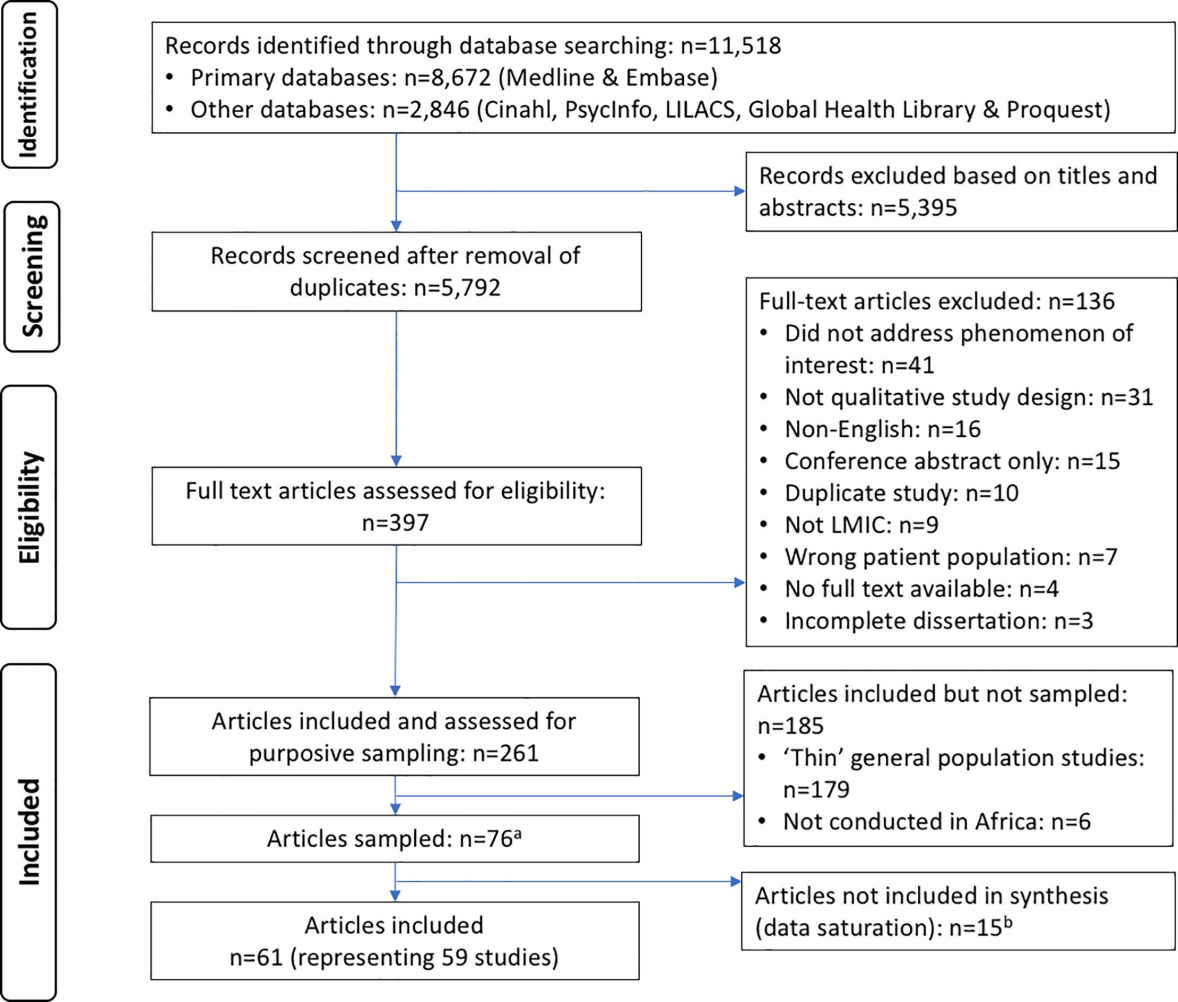
PRISMA flow diagram. ^a^ Sampled studies include those characterized as (1) conducted in Africa, and ‘thick’, or (2) ‘thin’ and belonging to specific population group of interest: A ‘thick’ study was defined according to the depth of analysis which included the following features: 1) the extent to which the authors transformed/analysed their findings, 2) insight into perspectives was demonstrated, 3) richness and complexity had been portrayed, and 4) there was theoretical or conceptual development. “Thin” studies were all other studies not classified as ‘thick’. Specific population of interest studies included those classified as thin but that evaluated perspectives which were of special interest for the review, including; provider perspectives, MSM populations, sex-worker populations; PWID populations; youth being transferred to new facilities; or conducted among refugee populations. ^b^Data saturation was reached.

Our search yielded a total of 11,518 records (Fig 1). After removal of duplicates, we screened titles and abstracts of 5792 records and full texts of 397 articles. We excluded 136 full-text articles with reasons. Of the 261 eligible articles, 185 articles were not included in the sample as they were either classified as ‘thin’ studies (n = 179), or were conducted in countries outside of Africa (n = 6). We purposively sampled 76 articles and included 61 articles [38–99], reporting on 59 studies, in the qualitative synthesis. The remaining 15 articles were not included in the synthesis as data saturation was reached.

### Studies included in the qualitative synthesis

A summary of the characteristics of studies included in the qualitative synthesis is presented in Table 1. Of included studies 15 were conducted in South Africa, 9 in Uganda, 8 in Tanzania, 4 in Swaziland, 4 in Zimbabwe, 3 each in Ethiopia, Kenya, Malawi, and Zambia, and 1 each in Cameroon, Lesotho, Mozambique, Nigeria, Rwanda, Kenya, Nigeria, and Tanzania. Studies examined factors influencing linkage to care, adherence to ART or retention in care amongst adults (n = 22), adolescents or children (n = 10), adults, adolescents and children (n = 2), men (n = 4), women (n = 4), pregnant or postpartum women (n = 7), female sex workers (n = 3), people who inject drugs (PWID) (n = 2), people living with disabilities (PWD) (n = 2), men who have sex with men (MSM) (n = 2) and refugees (n = 1). Studies used a variety of methods to collect data, including in-depth interviews, focus-group discussions, ethnography, participant observation, role-play and photo-elicitation interviews, most studies used a combination of methods. In-depth interviewing was the most common method of collecting data. Study participants comprised HIV-positive as well as a few HIV negative people, caregivers of HIV-positive children and adolescents, healthcare workers, traditional leaders, lay counsellors, community health workers and other key informants.

**Table 1:** Characteristics of included studies.

Study	Country	Phenomenon of interest	Data collection methods	Study participants
*General adults*				
Appelbaum Belisle 2015	South Africa	Concurrent use of traditional African medicine and ART	In-depth interviews and focus group discussions	HIV positive adults, health workers, HIV counsellors, traditional healers n = 26
Asgary 2014	Ethiopia	Beliefs about prevention, treatment, alternative cures and role of spiritual leaders in health education	In-depth interviews and focus group discussions	Adults (positive and negative HIV status), non-health personnel at health facilities, school staff, health workers, medical providers, community leaders n = 52
Assefa 2014	Ethiopia	Understanding interventions at health facilities and community-based organisations that improve retention in care	In-depth interviews and focus group discussions	Clinicians, adherence counsellors, case managers, adherence supporters, community-based service providers n = 72
Axelsson 2015	Lesotho	Adherence patterns, barriers to and facilitators of adherence to ART	In-depth interviews	HIV positive adults n = 28
Barfod 2013				
Beckmann 2013	Tanzania	Understanding how HIV-positive people navigate life and the complex treatment regimens	Ethnographic observations, in-depth interviews	HIV positive adults n = not reported
Bezabhe 2014	Ethiopia	Barriers and facilitators to adherence	In-depth interviews and focus group discussions	HIV positive adults and healthcare workers n = 58
Bhagwanjee 2013	South Africa	Impact of gender roles and relationships on HIV adherence	In-depth interviews	Seroconcordant couples n = 24
Bogart 2013	South Africa	Barriers to linkage of care	In-depth interviews and focus group discussions	HIV positive adults and healthcare workers n = 62
Braga 2013	Mozambique	Experience of living with HIV and interacting with health services	Ethnographic observations, in-depth interviews	HIV positive adults, health workers, lay counsellors, policy makers, traditional healers, faith healers n = 223
Campbell 2015	Zimbabwe	Patient labelling as good or bad patients and how it affects patient experiences at clinic visits	Ethnographic observations, in-depth interviews and focus group discussions	HIV positive adults, caregivers of children with HIV, health workers n = 104
Jones 2015	South Africa	Treatment seeking behaviours of HIV positive adults	Ethnographic observations and in-depth interviews	HIV positive adults, health workers, lay counsellors n = 35
Katz 2015	South Africa	Experiences linked to transfer of care from PEPFAR funded clinic to state funded clinics and the impact on retention in care	In-depth interviews	HIV positive adults n = 36
Layer 2014	Tanzania	Barriers and facilitators to linkage and retention in care	In-depth interviews and participant observation	HIV positive adults, health workers, traditional healers, spiritual healers and lay counsellors n = 221
Layer 2014	Tanzania	Experiences and factors influencing decisions to disengage from HIV care	In-depth interviews	HIV positive adults who had disengaged from care n = 14
Maeri 2016	Kenya	Experiences and beliefs about HIV disclosure among couples	In-depth interviews	HIV positive and negative adults, health workers, health managers, lay counsellors, research assistants, community health assistants and peer educators n = 194
Masquillier 2015	South Africa	Role and influence of household in supporting adherence and retention in care	In-depth interviews and focus group discussions	HIV positive adults and community health workers n = 68
Naik 2013	South Africa	Factors that influence linkage to HIV care	In-depth interviews	HIV positive adults n = 30
Niehaus 2014	South Africa	Factors other than treatment literacy, that influence linkage and adherence	Ethnography, case study	Life history of a man living with HIV/AIDS n = 1
Okoror 2013	Nigeria	Cultural context of HIV stigma on adherence	In-depth interviews and focus group discussions	HIV positive adults n = 35
Rasmussen 2013	Uganda	HIV counselling practices	Participant observation and in-depth interviews	HIV positive adults, lay counsellors n = 12
Russell 2016	Uganda	Influence of health care providers on HIV self-management	In-depth interviews	HIV positive adults on ART n = 38
Russell 2016		Barriers and facilitators to HIV self-management		
Ware 2013	Nigeria Uganda and Tanzania	Reasons for missed clinic visits and disengagement from HIV care	In-depth interviews	HIV positive adults who had disengaged from care n = 91
*Adults and children/adolescents*				
Scott 2014	Zimbabwe	Role of community networks in facilitating access and adherence to ART	In-depth interviews, focus group discussions and participant observations	HIV positive adults, caregivers of HIV positive children, health workers n = 127
Inzaule 2016	Uganda	Barriers and facilitators to long-term adherence	In-depth interviews and focus group discussions	HIV positive adults and adolescents in long-term HIV care (expert clients), health workers, lay counsellors n = 33
*Adolescents/children*				
Hornschuh 2014	South Africa	Experience of HIV care amongst HIV positive adolescents and young adults	Focus group discussions	HIV positive adolescents and young adults n = 18
Mburu 2014	Zambia	Adolescents' experiences with disclosure	In-depth interviews and focus group discussions	HIV positive adolescents, caregivers and health workers n = 170
Mutumba 2015	Uganda	Challenges and coping strategies of perinatally infected HIV positive adolescents	In-depth interviews	HIV positive adolescents n = 38
Mutwa 2013	Rwanda	Barriers and facilitators to adherence in HIV positive adolescents	In-depth interviews, focus group discussions and role play	HIV positive adolescents and their caregivers n = 52
Wolf 2014	Kenya	Reasons for disengagement in HIV care among adolescents	In-depth interviews and focus group discussions	HIV positive adolescents who had disengaged from care, community health workers and educators n = 55
Busza 2014	Zimbabwe	Barriers and facilitators of caregivers of children with HIV to retention in care	In-depth interviews	Caregivers of children with HIV, key informants from community-based support services n = 24
Kawuma 2014	Uganda	Barriers to adherence during childhood and adolescence	In-depth interviews	HIV positive children and adolescents, caregivers and health workers n = 26
Mattes 2014	Tanzania	Children and adolescents' experiences of living with HIV	Participant observation, in-depth interviews, thematic drawing and photo-elicitation interviews	HIV positive children and adolescents, caregivers n = 24
Coetzee 2015	South Africa	Barriers and facilitators to adherence in HIV positive children	In-depth interviews and focus group discussions	Caregivers of children with HIV, health workers, HIV counsellors and traditional healers n = 42
Sikstrom 2014	Malawi	Challenges to successful long-term paediatric ART	In-depth interviews, participant observation and in-depth case studies	HIV positive children and their caregivers, key informants (religious leaders, health care workers, grandparents, traditional healers, farmers, landowners and self-employed migrants) n = 104
*Men*				
Mburu 2014	Uganda	Influence of stigma and masculinity on engagement in HIV care	In-depth interviews and focus group discussions	HIV positive men and women, household members, health workers, health workers, health managers, lay counsellors, community leaders n = 65
Sikweyiya 2014	South Africa	The influence of HIV on the lives of men and their sense of masculinity	In-depth interviews	HIV positive men n = 18
Siu 2013	Uganda	Men's construction of masculinity and its influence on HIV treatment seeking behaviour	In-depth interviews and participant observation	HIV positive and negative men n = 26
Siu 2014		Influence of HIV on gender relations and men's sense of masculinity		
Zissette 2016	South Africa	Influence of masculinity on access to and utilisation of HIV care	In-depth interviews and participant observation	HIV positive men n = 21
*Women*				
Dlamini-Simelane 2016	Swaziland	Factors influencing access to care amongst married women with HIV	Ethnographic observations and in-depth interviews	Married women with HIV n = 2
Mbonye 2016	Uganda	Perceptions related to early linkage (test and treat)	In-depth interviews	HIV positive and negative women n = 15
Watt 2017	South Africa	Impact of sexual trauma on HIV care	In-depth interviews	HIV positive women with sexual trauma histories n = 15
Wouters 2016	South Africa	Women's experience of living with HIV and accessing care	In-depth interviews	HIV positive women n = 12
*Pregnant/postpartum women*				
Elwell 2016	Malawi	Factors influencing retention in Prevention of Mother to Child Transmission (PMTCT) programmes	In-depth interviews, focus group discussions and participant observations	HIV positive pregnant or postpartum women, health workers, community leaders n = 95
Gourlay 2014	Tanzania	Influence of patient-provider interactions on PMTCT uptake	Participatory learning and action group activities (includes focus group discussions, role-playing, creating maps), in-depth interviews, participant observation	HIV positive and negative women and men, health workers and health officials n = 91
Hatcher 2016	South Africa	Influence of intimate partner violence (IPV) on PMTCT uptake and adherence	In-depth interviews	HIV positive women experiencing IPV n = 32
Katirayi 2016	Swaziland	Barriers and facilitators to linkage and adherence of Option B+	In-depth interviews and focus group discussions	HIV positive pregnant or postpartum women and health workers n = 132
Kim 2016	Malawi	Barriers and facilitators to linkage and adherence of Option B+	In-depth interviews	HIV positive pregnant or postpartum women n = 65
McMahon 2016	Tanzania	Factors influencing disengagement from PMTCT care	In-depth interviews	HIV positive pregnant or postpartum women who had disengaged from HIV care n = 40
Ngarina 2013	Tanzania	Barriers to adherence post delivery	In-depth interviews	HIV positive women with detectable viral load after 24 months of delivery n = 23
*Female sex workers*				
Fielding-Miller 2014	Swaziland	Experience and health service needs of sex workers	In-depth interviews	HIV positive sex workers n = 20
Mtetwa 2013	Zimbabwe	Barriers to linkage and retention in care among sex-workers	Focus group discussions	HIV positive sex workers n = 38
Nakanwagi 2016	Uganda	Facilitators and barriers to linkage to HIV care among female sex workers	In-depth interviews	HIV positive sex workers, peer educators, NGO outreach workers n = 44
*People who inject drugs (PWID)*				
Guise 2016	Kenya	Barriers and facilitators to HIV care amongst People who inject drugs (PWID)	Ethnographic observations and in-depth interviews	HIV positive people who inject drugs n = 118
Saleem 2016	Tanzania	Barriers and facilitators to linkage to care among people who inject drugs	In-depth interviews	HIV positive people who inject drugs attending a methadone clinic, health care providers n = 32
*People living with disabilities*				
Parsons 2015	Zambia	Experiences of HIV positive adults with disabilities and how this influences access to HIV care	In-depth interviews	HIV positive adults with disabilities n = 32
Yoshida 2014	Zambia	Experience of HIV positive people with disabilities	In-depth interviews	HIV positive adults with disabilities n = 21
*Men who have sex with men (MSM)*				
Cange 2015	Cameroon	Influence of stigma on men who have sex with men (MSM) mental health and access to HIV care	In-depth interviews and focus group discussions	MSM (regardless of HIV status) n = 310
Kennedy 2013	Swaziland	Experiences and health needs of HIV positive MSM	In-depth interviews and focus group discussions	HIV positive MSM, HIV programme planners, policy makers, health workers, community leaders n = 62
*Refugees*				
Mendelsohn 2014	Kenya and Malaysia	Factors influencing adherence and retention in care amongst refugees	In-depth interviews	HIV positive refugees n = 26

### Quality

Critical appraisal of the 61 included papers demonstrated that the studies were of high quality with the majority making a ‘fairly thorough attempt’ at increasing rigor in sampling (57; 93%) and data collection (52; 85%), and grounding the findings in the data (54; 88%). Studies scored less well on ‘rigor in analysis’, and the ‘breadth and depth of their findings’, with 41 (67%) and 40 (66%) being assessed as having made a ‘fairly thorough attempt’ respectively. This lower score for some studies was mostly due to poor reporting of analysis techniques and a lack of transformation of the data beyond lists of the main themes. Critical appraisal findings are presented in S2 Table.

### Context specific sub-themes and codes

Codes and themes emerged from a variety of settings repeatedly and reflected commonalities across population groups. There were however a few codes and sub-themes which were unique to particular settings. For example, the use of holy-water as an alternative or adjunctive treatment for HIV was described in studies from Ethiopia, the provision of a financial ‘disability grants’ to HIV-positive people was uniquely described in South Africa, and issues related to adolescents and children in boarding school settings emerged predominantly from East Africa (Uganda, Kenya, Rwanda and Zimbabwe). Challenges pertaining to same day ART initiation were primarily described by women in antenatal services. There were very few examples of these context specific themes as most findings emerged from several African settings and this is highlighted in the supporting evidence (S1–S9 Evidence Annexes).

### Themes

We identified nine themes and grouped these into three main areas: (1) HIV-positive people navigate a complicated world, (2) the health system is punishing and uninviting, and (3) adapting and incorporating ART into life (Table 2).

**Table 2:** The nine emergent themes.

**HIV-positive people navigate a complicated world** 1: Poverty, competing priorities and an unpredictable microworld 2: Social identity and gender norms can have a profound impact on care-seeking behavior 3: Alienation makes it hard to take ART 4: People with HIV receive conflicting information, messages and views **The health system is punishing and uninviting** 5: “Bad patients” are an unhelpful construct of an authoritarian health system 6: Poor clinic services for patients and inadequate support for health workers **It is difficult to adapt to and incorporate ART into life** 7: The new normal requires daily drugs 8: Self-efficacy, social responsibility and support helps 9: The tipping point

Theme 1: Poverty, competing priorities and an unpredictable microworld (S1 Evidence Annex)

The lives of those who live in poverty and are HIV-positive contain many short and long-term competing priorities, complicated by frequent personal life events and external disruptions.

Poverty and the need to prioritize: Poverty creates economic pressures which forces many HIV-positive people in Africa to make difficult choices daily. People may choose to earn an income [41, 46, 48, 62, 80, 81, 97, 100–104], address food insecurity [41, 51, 65, 70, 75, 81, 86, 105], or personal safety [50, 57, 68, 100] over sorting out long-term HIV treatment; and the cost in terms of time and money of getting to services, creates a further obstacle [46, 48, 80, 102]. Survival for some requires them to remain socially connected, and as a result HIV-positive people, particularly women, may choose to maintain their social network rather than take ART, especially if HIV disclosure will compromise economic support systems [43, 51, 62, 68, 69, 81, 94, 100, 103, 106–108].

Unpredictable life events: Life events regularly disrupt routines; intermittent and unpredictable changes in family responsibilities [41, 94, 100], or the sudden onset of refugee status [75] can lead to a reprioritization of immediate needs. Further disruptions such as loss of support systems [75, 90] or unexpected problems with travel in highly mobile populations [57, 59, 81, 94, 100, 104] create structural obstacles to attending health services and contribute to gaps in ART care.

Theme 2: Social identity and gender norms can have a profound impact on care-seeking behavior (S2 Evidence Annex)

Social identity influences how HIV-positive people respond to HIV and ART. When individuals do not fulfil their societal defined roles, they may be judged harshly and ostracized. Many HIV-positive people strive to avoid discrimination and maintain their social identity, and this is sometimes at odds with initiating or continuing ART.

Gender roles impact health care decisions: Women have socially defined roles which often revolve around reproduction and caring for the family [85, 97, 108]. Competing maternal and marital responsibilities make it difficult for them to put their health first [54, 97, 109]. Poor women are often economically dependent on their partners and frequently disempowered in the household: they may not be able to make health care decisions on their own [61, 65, 107, 108], and are vulnerable to discrimination and abuse [54, 57, 65, 68, 69, 78, 100, 107–110]. HIV disclosure [81, 108] and infertility [85, 97, 108] may put women at risk of discrimination, social isolation and put them and their children’s survival at risk. To maintain their physical and economic safety, women carefully negotiate personal relationships and social networks in order to care for their children and themselves [57, 81, 103, 107].

Many men try to fulfill hegemonic representations of masculinity and want to be: strong and powerful [73, 92, 93], respected in society [73, 86, 91–93, 99], and father children [91, 99], work and provide economically for their families [73, 86, 89, 91, 92, 99]. This was hard after HIV diagnosis, as many men struggled with loss of power and respect in society, and at home [73, 91–93]. Attending HIV services requires both the acceptance of a weakened state and entry into traditionally female spaces [73, 91, 92, 99]. As a result, the requirements of long-term HIV care may be at odds with maintaining masculine identity and many men only seek care when a marked clinical deterioration is apparent [73, 91, 92, 99].

It is confusing and isolating being a child or adolescent with HIV: Children and adolescents with HIV desperately want to ‘fit in’ with their peer group [77, 78, 111]; adolescents want to plan for their future[70], have sexual relationships [70, 77], and they struggle to navigate all of this in the context of being HIV-positive. Some respond with anger and rebellion [78, 111]; others become isolated, secretive and depressed [77, 78, 111]. They remain dependent on adults for care and supervision and need stable home and school environments to thrive [59, 77, 78, 90]. Despite these needs, the support systems for HIV infected children and adolescents are often fragmented and change frequently [51, 78, 90]. Paediatric drug formulations for young children may not be palatable, and drug side-effects [51, 70], and lack of privacy for adolescents [51, 58, 59, 77, 78, 96, 111] makes adhering to ART particularly difficult. This is all compounded by adults not knowing how to deal with children who are HIV-positive. Caregivers may delay disclosure of HIV status, due to guilt about their own HIV-status, concerns about children’s cognitive abilities and social norms regarding discussion of sexuality with children [59, 72]. Health care workers often do not communicate directly or appropriately with children [51, 70, 72, 96], and this is exacerbated when children are transferred to non-paediatric adult or general HIV services [58, 59]. Poor communication creates confusion about HIV and ART, and when combined with fear of rejection and secrecy, depression and incoordinate support systems can markedly compromise adherence in this group [59, 70, 111].

Lack of conformity leads to double stigma for HIV-positive key populations: HIV-positive key populations face marked discrimination related to their life-style. MSM, PWID and sex-workers all do not conform to social norms and as a result are morally judged and discriminated against by others [50, 54, 64, 80, 88]. This discrimination is often worse than that experienced by those who are HIV-positive alone [50, 54, 64] and may cause psychological distress [50, 64], compounded by criminalization of MSM and sex-work in several African settings [50, 54, 88]. As a result, key-populations often have very limited support [50, 64, 76, 88] and for PWID, even with excellent support, drug addiction may trump any consideration of health status [56]. Discrimination plays a large role in keeping key populations away from general HIV services [64, 76, 88].

HIV-positive people with disabilities (PWD) defy social expectations: Society often assumes people with disabilities are asexual, and sub-human, and as a result HIV-positive PWD describe being discriminated against on many levels [84, 112]. This and structural obstacles to accessing care, may cause some to disengage [84].

Theme 3: Alienation makes it hard to take ART (S3 Evidence Annex)

Stigma alienates HIV-positive people from their social group and reduces their motivation to take ART. Fear of the consequences of HIV disclosure further impacts adherence and engagement in care.

HIV stigma and discrimination, makes it hard to take ART: HIV is associated with sexual promiscuity, being infectious and close to death [46, 67, 77, 78, 81, 84, 103]. People judge HIV-positive people morally and fear HIV transmission resulting in discrimination by partners, family and friends, employers, educators, and the general community [46, 59, 70, 72, 77, 83, 84, 111]. After disclosure of HIV status people may lose their emotional and financial support systems [39, 67–69, 83, 91, 103, 108]. In addition, some feel ashamed of their HIV status, blame themselves for becoming infected and internalize stigma [46, 67, 73, 83, 84, 103]. Discrimination and internalized stigma can make people feel isolated, hopeless and depressed [39, 46, 64, 67, 83]. It is hard for HIV-positive people to maintain adherence and engage in HIV care when they feel sad, alone and unsupported [46, 81, 100, 103]. Due to the anticipated negative consequences of disclosure people will go to great lengths to keep their HIV status secret, this secrecy makes it difficult to take ART regularly and attend HIV clinic services [46, 48, 53, 68, 79, 83, 94, 104], particularly if disclosure has not occurred in the household [48, 57, 65, 68, 69, 77, 78, 81, 91, 96]. Exposure at HIV clinics creates further challenges in maintaining confidentiality, and if the clinic location, design or staff compromise this, HIV-positive people will disengage from care [46, 47, 53, 56, 67, 74, 79].

Theme 4: People with HIV receive conflicting information, messages and views (S4 Evidence Annex)

Competing ideologies, HIV treatment options and experiences, combined with frequently changing HIV guidelines can create uncertainty about initiating or maintaining ART.

Alternative discourses about HIV and ART: There are many alternative discourses, beliefs and sources of information about the causes and treatment of HIV [38, 39, 43, 67, 74, 79, 80, 82, 91, 104, 105]. People’s beliefs may be informed by religious leaders, politicians, traditional healers, mass media, community members, friends and family [39, 67, 80, 82]. All of these influences can facilitate or hinder engagement. Religious and spiritual healers can sometimes offer better explanations and treatments, especially if those belief systems are well established and trusted by communities [38, 67, 79, 80, 91]. Ideologies which offer a cure are particularly appealing to HIV-positive people [39, 67, 74, 91, 104].

Side-effects undermine the message of improved health on ART: Drug side-effects and deterioration on treatment creates doubt regarding the health benefits of ART, and those who feel well when they initiate treatment may as a consequence disengage from care [65, 74, 87, 104]. Fear of such a deterioration prevents others from linking to care in the first place [47, 80].

Scientific uncertainty confuses patients and reduces confidence in the biomedical health system: Frequent changes to ART guidelines such as reduced CD4 thresholds for treatment initiation and guidance on risks of transmission between couples on ART can confuse patients and reduce confidence the biomedical health system [43, 61, 71, 82].

Choosing an ideology and treatment: People navigate through all the information, their experiences and those of others, weigh up the options and choose the ideologies which fit their needs best at a particular time [43, 82].

Theme 5: “Bad patients” are an unhelpful construct of an authoritarian health system (S5 Evidence Annex)

The authoritarian biomedical health system expects HIV-positive people to adhere to ART. Those who are able to comply are treated well, but when they fail to adhere they may be punished and disrespected.

Authoritarian health system: The biomedical health system and HIV care is delivered within an authoritarian and paternalistic health system [49, 55, 85]. Health workers control people’s clinic experience, decide who will get ART, and set the rules which HIV-positive people are expected to live by [43, 47, 49, 85, 94]. This power imbalance creates a situation where HIV-positive people work hard to carry favor with health workers [43, 49, 55, 85, 87] and some health workers are disrespectful to HIV-positive people [46, 47, 67, 94] because they have few other options for care.

Rules and responsibility: HIV-positive people are expected to follow an explicit set of rules in the form of ART and lifestyle—‘positive living’–guidelines [41, 45, 47, 49, 53, 54, 60, 82, 85, 87, 90, 92, 104]. They are advised; to adhere to clinic appointments and the ART treatment schedule; to obey clinic rules [41, 49, 85, 87]; to understand HIV medical terms [47, 60, 82, 87]; to stop drinking alcohol; to reduce sex [82, 85, 87, 93]; to avoid pregnancy [53, 85, 87]; to eat ‘healthy foods’ [44, 45, 54, 87, 90, 104]; to reduce ‘stress’ [43, 77, 87, 97]; to embrace their HIV-positive identity; and to help other HIV-positive people [43, 87]. Staff expect individuals to adhere to these rules [43, 46, 47, 82, 85] and often ignore how the realities of people’s lives may interfere with the ability to be compliant [43, 53, 85, 87, 90]. For some, this rules-based system works well [45, 55, 86, 87, 91], they adhere and they gain self-confidence [86, 91]; but others who cannot adhere, feel stressed, ashamed, and guilty [43, 60, 66, 94]; and are then labelled ‘drop-outs’, ‘defaulters’ or ‘bad patients’ [47, 49, 96], justifying mistreatment by health workers, who at times see this ‘failure’ as a personal characteristic, and morally judge, blame and inflict punishment [47, 94, 96]. This can drive those who are already struggling to maintain attendance to disengage from care entirely [43, 47, 49, 66, 67, 92, 94].

Theme 6: It is difficult and stressful attending public ART clinics (S6 Evidence Annex)

It is difficult, stressful and often unpleasant attending HIV services in the public sector; partly mirrored in health worker reflections of an overburdened and under-resourced service.

It is difficult and stressful attending public ART health services: The experience of attending HIV health services can be unpleasant. HIV-positive people may have to deal with rude health workers who disrespect and humiliate them [46, 47, 49, 53, 55, 62, 64, 66, 67, 92, 94, 104], inflexible and unaccommodating services with rigid policies [67, 94], long waiting times [41, 46, 48, 56, 62, 74, 79, 104] and unrealistic requirements [60, 80, 88], at times having to navigate confusing systems and unpredictable visits, where they cannot be sure they will receive the care they came for [46, 47, 49, 67]. For some, such services are unacceptable and they disengage from care [47, 49, 67, 79, 94].

It is hard work being a health care worker: Health care workers describe being over-burdened with excessive work-loads due to staff shortages and limited resources [53, 67, 88, 104], having inadequate support and training, and limited skills to deal with the weight of the social problems which they are presented with [67, 85]. They also describe feeling pressured to produce good treatment outcomes to feed back to program funders and managers [47, 85].

Theme 7: The new normal requires daily drugs (S7 Evidence Annex)

Accepting HIV and ART is not easy, it is influenced by perceived risk, time, health status, and belief in the biomedical rationale. Incorporating HIV and ART into a new version of life and identity, helps some adhere and engage in the long term.

Accepting HIV is difficult: Accepting HIV is hard and influenced by an individual’s perception of their own risk for infection prior to testing [79, 97]. A HIV-positive diagnosis is often met with shock [61, 65, 67, 74, 79, 97, 110], and many, at first, deny results and seek out repeat confirmatory tests at different health facilities [46, 61, 69, 74, 79, 80]. People struggle to reconcile how their lives, relationships and reproductive desires may have to change as a result of being HIV-positive [91, 97]. Some have maladaptive responses, feel hopeless and depressed [79–81, 91], or turn to substance abuse to cope [79, 91]. Over time, however, many accept the diagnosis and start to consider attending HIV services and initiation of ART [60, 69, 74, 79, 80, 88, 95].

Time and health status can influence the decision to initiate or maintain ART: The lack of time to prepare for ART is a particular challenge for those offered ART on same day as diagnosis, particularly if they feel well at the time [61, 65, 71, 74, 81]. Those who are healthy when they test HIV-positive or initiate ART are often poorly motivated to link to care or maintain ART [60, 61, 67, 71, 74, 79, 81, 88]; but those who have experienced a decline in health due to HIV and recovered on ART, feel highly motivated, at least initially, to engage in care [65, 67, 70, 73, 78, 83, 93, 104]. Once health is restored, people want to re-integrate into social life and feel normal, and taking ART sometimes impairs the ability to do so, as daily medication and clinic attendance interferes with the sense of leading a ‘normal’ life [43, 78, 85].

Accepting the medicalization of life: Over time many HIV-positive people learn to accept the medicalization of their lives [60, 61, 87, 97] and make several lifestyle changes to adapt to ART [47, 71, 83, 91, 93]. This is not always easy and can result in selective adherence to some rules and adaptation of others [41, 86, 87]. For some accepting the biomedical rationale and feeling like they can take control HIV by adhering to ART guidelines helps [86, 97, 99], this is however not essential and several people maintain alternative beliefs about HIV, and still adhere to ART [39, 65, 67, 78, 82, 91]. Some manage to incorporate HIV and ART into a new hybrid identity allowing them to feel like they can live a ‘normal’ life, with HIV [40, 79, 86, 91, 93, 97]. There are a few select HIV-positive people who fully embrace their HIV status and create an advocate identity which is defined by being HIV-positive [43, 98, 99]. But even among those who accept HIV and ART, many still harbor a deep desire to be rid of the disease and hope for a cure [43, 70, 75, 97].

Theme 8: Personal motivation and support helps (S8 Evidence Annex)

Inherent self-efficacy and a desire to fulfill social responsibilities help HIV-positive people feel motivated to take ART, and emotional, practical or financial support helps them feel accepted, cope with challenges, and adhere to ART.

Self-efficacy varies: How HIV-positive people respond to an HIV diagnosis and life-long treatment is variable, with individuals demonstrating different self-management abilities (self-efficacy) and thresholds for disengagement [57, 79, 86]. Some, despite substantial challenges maintain adherence, and others are driven out of care by what appear to be smaller obstacles [57, 79, 86].

Having others to care for is a motivator: Although it is difficult for people to accept HIV and ART, having a family [57, 80, 89, 92, 99, 104] or an unborn child to protect and care for [53, 57, 61, 65, 69] can motivate HIV positive people to take ART.

Support helps: Social support helps HIV-positive people gain self-confidence and a sense of belonging and offsets the effects of stigma [86, 88, 89, 91]. Partners, friends, family members and others can facilitate HIV acceptance and motivation to take ART [41, 48, 68, 79, 86, 88, 96, 99]. Spirituality, religion and allopathic medicine practitioners also provide support, especially in settings where these practices and belief systems are firmly established [38, 67, 70, 77, 86]. HIV specific health services and peer groups can create a nurturing environment where HIV-positive people can meet, support each other and foster a sense of belonging [41, 67, 80, 86, 89]. HIV counselors offer messages of hope after diagnosis and try to help reframe HIV as a normal chronic disease for those struggling to accept their HIV-positive status [55, 79, 80, 86–88, 97]. Mutually respectful long-term relationships with health care providers are features of health services where patients feel supported [68, 86, 89]. Community organizations, community health workers and home based carers play a variety of roles in the community including providing palliative care to the very sick and encouragement to return to care for those who have disengaged [40, 65, 67, 89]. Provision of financial and food aid can help alleviate some of the struggles of poverty and allow patients to focus on their health, but in settings of severe poverty, dependence on such aid programs can have negative effects on compliance if aid is withdrawn or based on health status [60, 69, 89, 92, 104]. Overall, emotional, practical and financial support, for the most part plays a large role in helping HIV-positive people accept HIV and adhere to ART [45, 48, 64, 80, 88, 104].

Theme 9: The tipping point (S9 Evidence Annex)

Several influences interact and drive people in or out of care, with the final driver at times ascribed to disrespectful health workers or a deterioration in health.

Interacting factors and final events: There is a complex interplay between the individual, their environment, the health system, and time. These elements work together to influence engagement and adherence decisions [67, 74, 81, 94]. For example, disengagement for some, starts with one unexpected absence from care which when followed by various other obstacles leads to sustained disengagement [65–67, 94], and in a few instances a tipping point is described, where a sequence of events culminates in a final episode which drives HIV-positive people out of care, most often related to poor clinic services and disrespectful health workers [60, 65–67, 79, 94]. A similar final event or ‘tipping point’ is also described by some who return to care after an absence or link for the first time, and this decision to engage in care is frequently the result of a deterioration in health [59, 65, 67, 69, 74].

### Conceptual model

**Fig 2:**
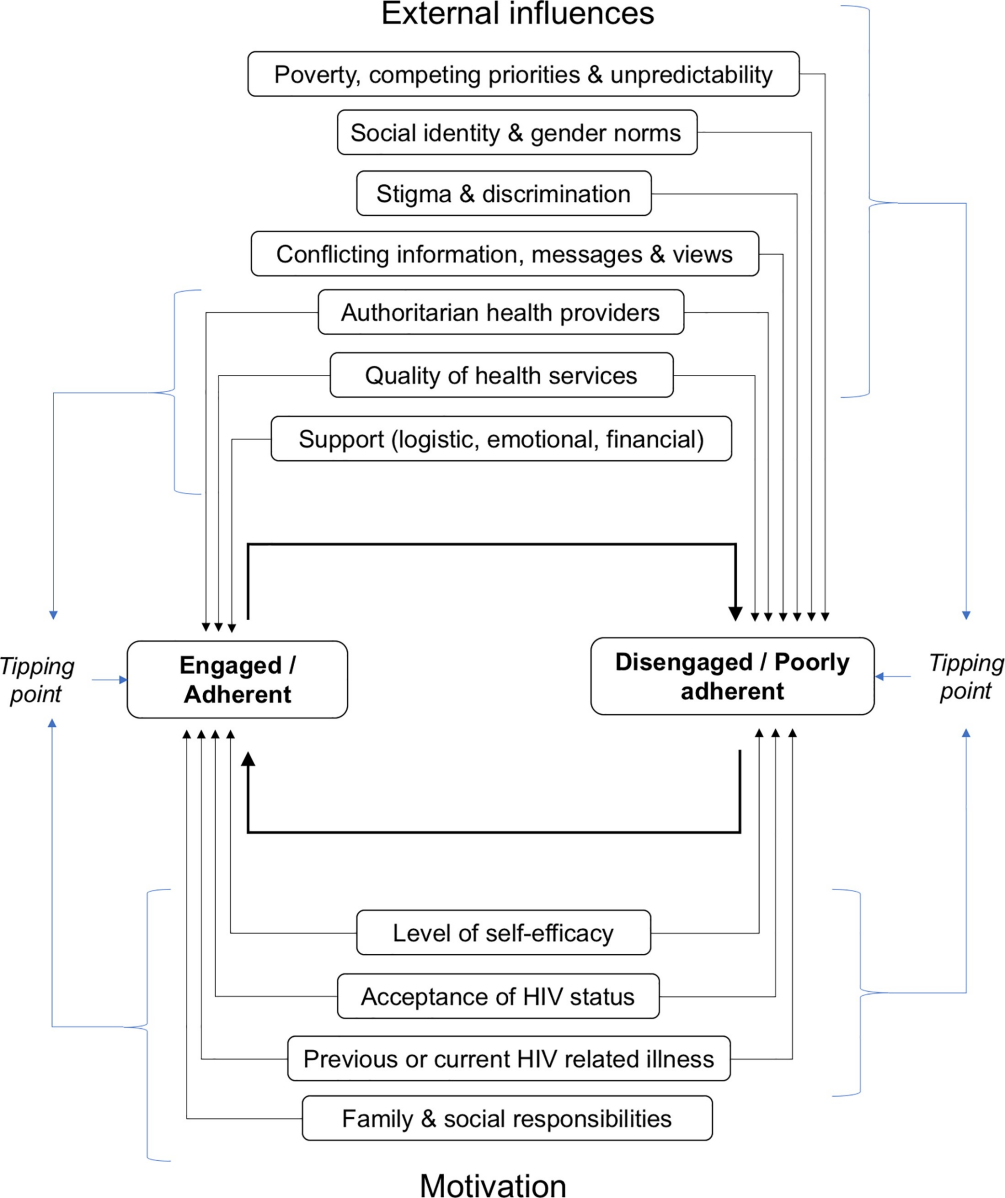
Theoretical model of influences on engagement and adherence to ART.

Based on these themes we developed a theoretical model demonstrating how multiple influences may act on an individual over time and result in changes in engagement and adherence behavior (Fig 2). The model demonstrates how several external influences -representing the effects of politics, society, and health systems—work to drive engagement and adherence behavior. This includes the negative effects of: living in poverty, where multiple competing priorities are constantly weighed up to ensure survival, and frequent unpredictable live events interfere with routines; social and gender norms may impair the ability to incorporate HIV and ART into daily life; and various sources and discourses about HIV and ART may confuse patients. We also highlight that the quality of clinic services can drive patients in or out of care, and that authoritarian rules-based health provision has a bidirectional effect, which may be valued by some patients but disliked by others who prefer to be partners in health care decisions. Logistic, emotional or final support from others: in the household, community, or at the health center, helps patients cope with challenges of taking ART and for the most part act as a positive external influence on engagement in care and adherence. The model further depicts how personal motivators also play a large role in determining treatment and care behavior: with high levels of self-efficacy and acceptance of the HIV diagnosis resulting in better adherence and engagement, and the desire to restore or maintain health for those who have been clinically ill acting as a motivator for many. Additionally, a sense of responsibility to care for and support family members, helps some remain in care and on treatment. Finally, we demonstrate how any combination of external influences and personal motivations may act together to tip patients in or out of care or change levels of adherence, and how HIV positive people may cycle in and out of these states over time. This theoretical model supports the ecological perspective of health behavior [113] and additionally represents how engagement is a dynamic process which fluctuates over the long course of HIV care.

## Discussion

This synthesis, across multiple African populations, revealed common patterns of how HIV-positive people make engagement and adherence decisions.

First, HIV-positive people constantly navigate a complex environment with numerous influences and barriers to accessing care. It was evident that living in poverty prevented many from being able to attend to their health needs either due to other priorities or disruptions, reflecting the pervasive problem of reduced access to health care for those who are poor [114]. It was however described that some of these challenges could be overcome with help from others, but that an HIV diagnosis often compromised support systems. It is well described that belonging to strong familial and extrafamilial social networks can ameliorate some of the difficulties of living in poverty, and that social networks provide social capital which can be drawn on during difficult times [115–117]. HIV-positive people also struggled to accept how their personal and social identity changed with HIV, a challenge faced by many who develop chronic illnesses [118], but made more difficult in the face of HIV-related stigma. HIV stigma posed a major threat to preserving social identity, with some choosing their social world and support networks over ART. The profound negative impact of stigma on ART adherence and engagement has been identified in Africa and other settings since early in the HIV epidemic, Devine and colleagues described in the 1990’s how ‘AIDS’ stigma specifically went beyond people’s fears of being infected and reflected a deeper ‘socially constructed phenomenon’ related to social identity and prejudice—it was seen as a ‘threat to core social values’ [119], findings which remain relevant in the current era [21, 25] and are mirrored in accounts of moral judgements and assumptions about sexual behavior in our synthesis. The descriptions of how stigma impaired HIV care seeking were particularly vivid among marginalized and vulnerable groups due to the intersectionality between various forms of stigma [120]. This was apparent among key populations experiences of ‘double stigma’ related to HIV and lifestyle, and women’s experiences of marked social and economic isolation following HIV disclosure. In addition to these substantial barriers to care seeking, alternative discourses about the causes and treatment of HIV frequently resulted in delayed engagement or disruptions in care. The influence of this medical pluralism on HIV care seeking in Africa cannot be underestimated, and in a recent large multi-country qualitative study, Moshabela and colleagues demonstrate how HIV-positive people ‘shop and switch’ between biomedical, traditional and faith based health care systems and how this leads to bottlenecks along the HIV care cascade if people use this as alternative rather than complementary care [121]. This contributed to people’s overall uncertainty about HIV care, which was in our exploration compounded by frequently changing HIV guidelines and drug side-effects which undermined overall confidence in ART and the biomedical health system.

Second, challenges faced when attending health services can drive HIV-positive people out of care. Accounts of authoritarian health systems, overburdened health workers, disrespectful staff and difficult clinic visits have been reported across health services in Africa and are not unique to HIV care [122–125], however the moral judgment and labelling of poorly adherent patients was an additional aspect which made it difficult for some to remain or return to care.

Third, individual characteristics, time and support systems influence the response to a HIV diagnosis and the ability to maintain long-term adherence and engagement in care. Acceptance of HIV can take time [126] and it appears that the trajectory of becoming an adherent self-managing HIV-positive person follows a similar course to the processes of coping with other chronic illnesses, with people transitioning through several stages, including: learning about the condition, modifying lifestyle, activating psychological, spiritual and social resources, and finally learning to live with HIV and make meaning of it [127, 128]. In our data this process was positively influenced by strong support systems, previous HIV related illnesses and the desire to stay alive to care for dependents.

Finally, even for those who accepted HIV and adapted to ART, the numerous influences fluctuated and changed over time causing people to transition in and out of care, a pattern which has also been identified in HIV cohort data from high income settings [129].

This review goes beyond presenting barriers and facilitators and describes broadly how various external influences and personal motivation interact and drive ART adherence and engagement decisions, and presents a model for understanding ‘why people do what they do’. Our findings are strengthened by a detailed, rigorous and iterative process of data-extraction and synthesis involving the entire author team and a large body of supporting evidence presented in this paper and supplementary materials. We included primary studies from across the African continent and did not identify any major themes which occurred exclusively in any one setting, making these finding generalizable to HIV-positive people across Africa. Although there were no studies conducted in prisoners and transgender people which contributed to our synthesis, we believe that some of our findings regarding experiences of marginalized groups may translate to some extent to these populations, it is possible however that some of their unique challenges are missing from this review. There were few studies identified which provided the perspectives of health workers suggesting that the experiences of health workers deserve further research.

We believe this analysis can help provide a springboard at a national or local level for thoughtful decision making in policy, and for providers, government, non-governmental organizations, and advocacy groups to generate approaches at various levels and create long term solutions. Given the numerous primary studies identified in this review we suggest that researchers intending to conduct more qualitative studies in this field consider what may be different—in light of these findings, and what additional information may be needed to help inform the health policy decisions in their setting.

Our review search was conducted on 9 December 2016 and it is possible that some of the landscape regarding stigma and access to care may have changed in the ‘test and treat’ and differentiated service delivery era. However, after conducting an updated search and rapidly appraising recently published studies (up till 1 August 2018), we identified several studies which supported our findings, specifically regarding quality of health services [130–133], disrespectful health workers and the role of power in health service delivery [132, 134], lack of access to health services [130, 131], competing priorities and unpredictable life circumstances [135–138], the role of the social world [131, 132, 139], influence of gender roles [140], medical pluralism [132, 141], the motivating influence of having dependents [142, 143], the challenges of same day ART initiation[144], the barriers to care caused by stigma [130, 131, 138, 143, 145, 146], challenges to accessing care for PWD [147], the benefits of support [131, 132, 148, 149], and fluctuations in engagement over time [131]. We therefore feel that the findings of this review remain relevant to current HIV populations.

## Conclusions

This analysis broadly reflects what HIV-positive people grapple with on a day-to-day basis as they try to manage a highly stigmatized chronic life-threatening illness within the context of every-day life in Africa. Many of the barriers we identified, have previously been well-articulated and are obvious; and there are many systematic reviews of trials of technical fixes to assure adherence. What this review offers is a new theoretical model of the dynamics of adherence and engagement in care that can help providers understand their patients, their dilemmas, and how this plays out in real time in order to help with the design of service delivery approaches sensitive to what is happening in people’s lives. The themes and theory generated provide a basis for more informed thinking and action on the part of policy makers, providers, and society to understand what influences HIV-positive people in Africa, and how the attitudes and culture of societies and the health service needs to shift to help those with HIV to successfully lead more normal lives.

## Supporting information

S1 Appendix Differences between protocol and review. (DOCX)

S2 Appendix Search strategy. (DOC)

S3 Appendix Codes identified. (XLSX)

S4 Appendix ENTREQ checklist. (DOCX)

S1 Table Methodological quality appraisal tool. (DOCX)

S2 Table Critical appraisal of included studies. (DOCX)

S1 Evidence Annex Theme 1—Poverty, competing priorities and an unpredictable microworld. (DOCX)

S2 Evidence Annex Theme 2—Social identity and gender norms can have a profound impact on care-seeking behavior. (DOCX)

S3 Evidence Annex Theme 3—Alienation makes it hard to take ART. (DOCX)

S4 Evidence Annex Theme 4—People with HIV receive conflicting information, messages and views. (DOCX)

S5 Evidence Annex Theme 5 - “Bad patients” are an unhelpful construct of an authoritarian health system. (DOCX)

S6 Evidence Annex Theme 6—Poor clinic services for patients and inadequate support for health workers. (DOCX)

S7 Evidence Annex Theme 7—The new normal requires daily drugs. (DOCX)

S8 Evidence Annex Theme 8—Self-efficacy, social responsibility and support helps. (DOCX)

S9 Evidence Annex Theme 9—The tipping point. (DOCX)
