# Supplementary material for: Being HIV positive and staying on antiretroviral therapy in Africa: A qualitative systematic review and theoretical model
Source: PLoS One. 2019 Jan 10;14(1):e0210408. doi: 10.1371/journal.pone.0210408 (PMC6328200; doi:10.1371/journal.pone.0210408)
Supplement: S1 Appendix — (DOCX) [file pone.0210408.s001.docx]

# Differences between protocol and final review

1. We intended to include studies from all low and middle income countries, however due to limited studies from settings outside of Africa, we restricted the setting to Africa
2. We applied the purposive sampling technique to our included studies, which was not specified in our protocol. The number of included studies was higher than expected, we therefore employed this technique to help us evaluate the richest studies with the highest yield of information.
3. We had considered using GRADE-CERQual to evaluate quality of evidence contributing to themes, however due the way the themes emerged and the variable evidence contributing to all the codes and subthemes this became unwieldy to use and we opted rather to present overall methodological quality of the included studies
