# Supplementary material for: Being HIV positive and staying on antiretroviral therapy in Africa: A qualitative systematic review and theoretical model
Source: PLoS One. 2019 Jan 10;14(1):e0210408. doi: 10.1371/journal.pone.0210408 (PMC6328200; doi:10.1371/journal.pone.0210408)
Supplement: S2 Appendix — (DOC) [file pone.0210408.s002.doc]

**Search Strategy**

| Search | Query |
| --- | --- |
| [#70](https://www.ncbi.nlm.nih.gov/pubmed/advanced) | Search (#69) AND #45 Filters: Publication date from 2013/01/01; Field: Title/Abstract |
| [#45](https://www.ncbi.nlm.nih.gov/pubmed/advanced) | Search ((#44) AND #29) AND #24 Filters: Publication date from 2013/01/01; Field: Title/Abstract |
| [#69](https://www.ncbi.nlm.nih.gov/pubmed/advanced) | Search ((((((((((((((#68) OR #66) OR #65) OR #64) OR #63) OR #62) OR #61) OR #60) OR ("Self Report"[Mesh] AND ( "2013/01/01"[PDat] : "3000/12/31"[PDat] ))) OR ("Surveys and Questionnaires"[Mesh] AND ( "2013/01/01"[PDat] : "3000/12/31"[PDat] ))) OR ("Health Services Administration"[Mesh] AND ( "2013/01/01"[PDat] : "3000/12/31"[PDat] ))) OR (("Interviews as Topic"[Mesh] OR "Interview" [Publication Type]) AND ( "2013/01/01"[PDat] : "3000/12/31"[PDat] ))) OR ("Focus Groups"[Mesh] AND ( "2013/01/01"[PDat] : "3000/12/31"[PDat] ))) OR ((qualitative or ethno* or emic or etic or phenomenology* or hermeneutic* or heidegger* or husserl* or colaizzi* or giorgi* or glaser or strauss or "van kaam*" or "van manen" or "constant compar*") AND ( "2013/01/01"[PDat] : "3000/12/31"[PDat] ))) OR #47 Filters: Publication date from 2013/01/01; Field: Title/Abstract |
| [#61](https://www.ncbi.nlm.nih.gov/pubmed/advanced) | Search sampl* or ricoeur or spiegelberg* or merleau or metasynthes* or meta-synthes* or metasummar* or meta-summar* or metastud* or Filters: Publication date from 2013/01/01; Field: Title/Abstract |
| [#60](https://www.ncbi.nlm.nih.gov/pubmed/advanced) | Search "focus group*" or "grounded theory" or "narrative analys*" or "lived experience*" or "life experience*" or "theoretical sampl*" or purposive Filters: Publication date from 2013/01/01; Field: Title/Abstract |
| [#47](https://www.ncbi.nlm.nih.gov/pubmed/advanced) | Search "Qualitative Research"[Mesh] Filters: Publication date from 2013/01/01; Field: Title/Abstract |
| [#62](https://www.ncbi.nlm.nih.gov/pubmed/advanced) | Search "thematic* analys*" or "content analy*" or "field note*" or fieldnote* or "field record*" or "field stud*" or "participant* AND observ*" Filters: Publication date from 2013/01/01; Field: Title/Abstract |
| [#63](https://www.ncbi.nlm.nih.gov/pubmed/advanced) | Search "nonparticipant* AND observ*" Filters: Publication date from 2013/01/01; Field: Title/Abstract |
| [#64](https://www.ncbi.nlm.nih.gov/pubmed/advanced) | Search semi-structured or semistructured or "structured categor*" or "unstructured categor*" or "action research" or (audiorecord* or taperecord*or videorecord* or videotap*) or (audio or tape or video*) or interview* or quasi-experiment* or "case stud*" Filters: Publication date from 2013/01/01; Field: Title/Abstract |
| [#65](https://www.ncbi.nlm.nih.gov/pubmed/advanced) | Search semi-structured OR semistructured OR "structured categor*" OR "unstructured categor*" OR "action research" OR (audiorecord* OR tape recorded *or videorecord* OR videotap*) OR (audio OR tape OR video*) OR interview* OR quasi-experiment* OR "case stud*" Filters: Publication date from 2013/01/01; Field: Title/Abstract |
| [#66](https://www.ncbi.nlm.nih.gov/pubmed/advanced) | Search collaborat* or consultat* or experience or involve* or narrative* or opinion* or participat* or partner* or perspective* or story or Filters: Publication date from 2013/01/01; Field: Title/Abstract |
| [#68](https://www.ncbi.nlm.nih.gov/pubmed/advanced) | Search "Anthropology, Cultural"[Mesh] Filters: Publication date from 2013/01/01; Field: Title/Abstract |
| [#59](https://www.ncbi.nlm.nih.gov/pubmed/advanced) | Search "Self Report"[Mesh] Filters: Publication date from 2013/01/01 |
| [#57](https://www.ncbi.nlm.nih.gov/pubmed/advanced) | Search "Surveys and Questionnaires"[Mesh] Filters: Publication date from 2013/01/01 |
| [#55](https://www.ncbi.nlm.nih.gov/pubmed/advanced) | Search "Health Services Administration"[Mesh] Filters: Publication date from 2013/01/01 |
| [#53](https://www.ncbi.nlm.nih.gov/pubmed/advanced) | Search "Interviews as Topic"[Mesh] OR "Interview" [Publication Type] Filters: Publication date from 2013/01/01 |
| [#51](https://www.ncbi.nlm.nih.gov/pubmed/advanced) | Search "Focus Groups"[Mesh] Filters: Publication date from 2013/01/01 |
| [#49](https://www.ncbi.nlm.nih.gov/pubmed/advanced) | Search qualitative or ethno* or emic or etic or phenomenology* or hermeneutic* or heidegger* or husserl* or colaizzi* or giorgi* or glaser or strauss or "van kaam*" or "van manen" or "constant compar*" Filters: Publication date from 2013/01/01 |
| [#22](https://www.ncbi.nlm.nih.gov/pubmed/advanced) | Search "Anti-HIV Agents"[Mesh] Field: Title/Abstract |
| [#11](https://www.ncbi.nlm.nih.gov/pubmed/advanced) | Search antiretroviral* Field: Title/Abstract |
| [#13](https://www.ncbi.nlm.nih.gov/pubmed/advanced) | Search haart therapy Field: Title/Abstract |
| [#16](https://www.ncbi.nlm.nih.gov/pubmed/advanced) | Search "HIV"[Majr] Field: Title/Abstract |
| [#18](https://www.ncbi.nlm.nih.gov/pubmed/advanced) | Search "HIV Infections"[Majr] Field: Title/Abstract |
| [#24](https://www.ncbi.nlm.nih.gov/pubmed/advanced) | Search (((((#22) OR #18) OR #16) OR #13) OR #11) OR ((HIV[Title/Abstract] OR hiv-1[Title/Abstract] OR hiv-2*[Title/Abstract] OR hiv1[Title/Abstract] OR hiv2[Title/Abstract] OR “hiv infect*”[Title/Abstract] OR “human immunodeficiency virus”[Title/Abstract] OR “human immune deficiency virus”[Title/Abstract] OR “human immuno-deficiency virus”[Title/Abstract] OR “human immune-deficiency virus”[Title/Abstract] OR ((human immun*) AND(deficiency virus))[Title/Abstract] OR “acquired immunodeficiency syndromes”[Title/Abstract] OR “acquired immune deficiency syndrome”[Title/Abstract] OR “acquired immuno-deficiency syndrome”[Title/Abstract] OR “acquired immune-deficiency syndrome” [Title/Abstract] OR ((acquired immun*)[Title/Abstract] AND (deficiency syndrome))[Title/Abstract] OR HIV/AIDS[Title/Abstract])) Filters: Publication date from 2013/01/01; Field: Title/Abstract |
| [#29](https://www.ncbi.nlm.nih.gov/pubmed/advanced) | Search ((#28) OR #26) OR #25 Filters: Publication date from 2013/01/01; Field: Title/Abstract |
| [#25](https://www.ncbi.nlm.nih.gov/pubmed/advanced) | Search Retention OR retain* OR "lost to follow-up" OR ("loss*" AND "follow up") OR LTFU OR “loss-to-follow-up” OR attrition OR "loss to care" OR "loss to program*" OR default* OR engage* OR disengage* OR “retention in care” OR “lost to retention” OR “treatment initiation” OR link* OR “link to care” OR “link to treatment” OR “linkage to treatment” OR “link into care” OR “linkage into treatment” Filters: Publication date from 2013/01/01; Field: Title/Abstract |
| [#26](https://www.ncbi.nlm.nih.gov/pubmed/advanced) | Search Adherence OR adher* OR compliance OR complian* OR comply OR complied OR noncomplian* OR non-complian* OR non-adher* OR nonadher* Filters: Publication date from 2013/01/01; Field: Title/Abstract |
| [#28](https://www.ncbi.nlm.nih.gov/pubmed/advanced) | Search "Medication Adherence"[Mesh] Filters: Publication date from 2013/01/01; Field: Title/Abstract |
| [#44](https://www.ncbi.nlm.nih.gov/pubmed/advanced) | Search (((((((((((#43) OR #41) OR #40) OR #39) OR #38) OR #37) OR #36) OR #35) OR #34) OR #33) OR #32) OR #31 Filters: Publication date from 2013/01/01; Field: Title/Abstract |
| [#33](https://www.ncbi.nlm.nih.gov/pubmed/advanced) | Search Armenian or Azerbaijan or Bangladesh or Barbados or Benin or Byelarus or Byelorussian or Belarus or Belorussian or Belorussia or Belize or Bhutan or Bolivia or Bosnia or Herzegovina or Hercegovina or Botswana or Brazil or Bulgaria or Burkina Faso or Burkina Fasso or Upper Volta or Burundi or Urundi or Cambodia or Khmer Republic or Kampuchea or Cameroon or Cameroons or Cameron or Camerons or Cape Verde or Central African Republic or Chad or Chile or China or Colombia or Comoros or Comoro Islands or Comores or Mayotte or Congo or Zaire or Costa Rica or Cote d’Ivoire or Ivory Coast or Croatia or Cuba or Djibouti or French Somaliland or Dominica or Dominican Republic or East Timor or East Timur or Timor Leste or Ecuador or Egypt or United Arab Republic or El Salvador Filters: Publication date from 2013/01/01; Field: Title/Abstract |
| [#32](https://www.ncbi.nlm.nih.gov/pubmed/advanced) | Search Africa or Asia or Caribbean or "West Indies" or "South America" or "Latin America" or "Central America" Filters: Publication date from 2013/01/01; Field: Title/Abstract |
| [#31](https://www.ncbi.nlm.nih.gov/pubmed/advanced) | Search "developing countries" Filters: Publication date from 2013/01/01; Field: Title/Abstract |
| [#36](https://www.ncbi.nlm.nih.gov/pubmed/advanced) | Search eritrea OR ethiopia OR fiji OR gabon OR gabonese republic OR gambia OR gaza OR georgia republic OR georgia republic OR ghana OR gold coast OR grenada OR guatemala OR guinea OR guam OR guiana OR guyana OR haiti OR honduras OR india OR maldives OR indonesia OR iran OR iraq OR jamaica OR jordan OR kazakhstan OR kazakh OR kenya OR kiribati OR korea OR kosovo OR kyrgyzstan OR kirghizia OR kyrgyz republic OR kirghiz OR kyrgyzstan OR lao pdr OR laos OR latvia OR lebanon OR lesotho OR basutoland OR liberia OR libya OR lithuania OR macedonia OR madagascar OR malagasy republic OR malaysia OR malaya OR malay OR sabah OR sarawak OR malawi OR nyasaland OR mali OR marshall islands OR mauritania OR mauritius OR galega islands OR mexico OR micronesia OR middle east OR moldova OR moldova OR moldovan OR mongolia OR montenegro OR morocco OR ifni OR mozambique OR myanmar OR myanmar OR burma OR namibia OR nepal OR netherlands antilles OR new caledonia OR nicaragua OR niger OR nigeria OR northern mariana islands OR oman OR muscat OR pakistan OR palau OR palestine OR panama OR paraguay OR peru OR philippines OR philippines OR philippines OR philippines OR papua new guinea OR portugal OR romania OR rumania OR roumania OR russia OR russian OR rwanda OR ruanda OR saint lucia OR st lucia OR saint vincent OR st vincent OR grenadines OR samoa OR samoan islands OR navigator island OR navigator islands OR sao tome OR senegal OR serbia OR montenegro OR seychelles OR sierra leone OR sri lanka OR ceylon OR solomon islands OR somalia OR sudan OR suriname OR surinam OR swaziland OR south africa OR syria OR tajikistan OR tadzhikistan OR tadjikistan OR tadzhik OR tanzania OR thailand OR togo OR togolese republic OR tonga OR trinidad OR tobago OR tunisia OR turkey OR turkmenistan OR turkmen OR uganda OR ukraine OR uruguay OR ussr OR soviet union OR union of soviet socialist republics OR uzbekistan OR uzbek OR vanuatu OR new hebrides OR venezuela OR vietnam OR viet nam OR west bank OR yemen OR yugoslavia OR zambia OR zimbabwe Filters: Publication date from 2013/01/01; Field: Title/Abstract |
| [#35](https://www.ncbi.nlm.nih.gov/pubmed/advanced) | Search Eritrea or Ethiopia or Fiji or Gabon or Gabonese Republic or Gambia or Gaza or Georgia Republic or Georgian Republic or Ghana or Gold Coast or Grenada or Guatemala or Guinea or Guam or Guiana or Guyana or Haiti or Honduras or India or Maldives or Indonesia or Iran or Iraq or Jamaica or Jordan or Kazakhstan or Kazakh or Kenya or Kiribati or Korea or Kosovo or Kyrgyzstan or Kirghizia or Kyrgyz Republic or Kirghiz or Kirgizstan or Lao PDR or Laos or Latvia or Lebanon or Lesotho or Basutoland or Liberia or Libya or Lithuania or Macedonia or Madagascar or Malagasy Republic or Malaysia or Malaya or Malay or Sabah or Sarawak or Malawi or Nyasaland or Mali or Marshall Islands or Mauritania or Mauritius or Agalega Islands or Mexico or Micronesia or MiddleEast or Moldova or Moldovia or Moldovian or Mongolia or Montenegro or Morocco or Ifni or Mozambique or Myanmar or Myanma or Burma or Namibia or Nepal or Netherlands Antilles or New Caledonia or Nicaragua or Niger or Nigeria or Northern Mariana Islands or Oman or Muscat or Pakistan or Palau or Palestine or Panama or Paraguay or Peru or Philippines or Philipines or Phillipines or Phillippines or Papua New Guinea or Portugal or Romania or Rumania or Roumania or Russia or Russian or Rwanda or Ruanda or Saint Lucia or St Lucia or Saint Vincent or St Vincent or Grenadines or Samoa or Samoan Islands or Navigator Island or Navigator Islands or Sao Tome or Senegal or Serbia or Montenegro or Seychelles or Sierra Leone or Sri Lanka or Ceylon or Solomon Islands or Somalia or Sudan or Suriname or Surinam or Swaziland or South Africa or Syria or Tajikistan or Tadzhikistan or Tadjikistan or Tadzhik or Tanzania or Thailand or Togo or Togolese Republic or Tonga or Trinidad or Tobago or Tunisia or Turkey or Turkmenistan or Turkmen or Uganda or Ukraine or Uruguay or USSR or Soviet Union or Union of Soviet Socialist Republics or Uzbekistan or Uzbek or Vanuatu or NewHebrides or Venezuela or Vietnam or Viet Nam or West Bank or Yemen or Yugoslavia or Zambia or Zimbabwe Filters: Publication date from 2013/01/01; Field: Title/Abstract |
| [#34](https://www.ncbi.nlm.nih.gov/pubmed/advanced) | Search armenian OR azerbaijan OR bangladesh OR barbados OR benin OR byelarus OR byelorussian OR belarus OR belorussian OR belorussia OR belize OR bhutan OR bolivia OR bosnia OR herzegovina OR hercegovina OR botswana OR brazil OR bulgaria OR burkina faso OR burkina faso OR upper volta OR burundi OR urundi OR cambodia OR khmer republic OR kampuchea OR cameroon OR cameroons OR cameron OR cameron OR cape verde OR central african republic OR chad OR chile OR china OR colombia OR comoros OR comoro islands OR comores OR mayotte OR congo OR zaire OR costa rica OR cote d'ivoire OR ivory coast OR croatia OR cuba OR djibouti OR french somaliland OR dominica OR dominican republic OR east timor OR east timur OR timor leste OR ecuador OR egypt OR united arab republic OR el salvador Filters: Publication date from 2013/01/01; Field: Title/Abstract |
| [#37](https://www.ncbi.nlm.nih.gov/pubmed/advanced) | Search (developing or "less* developed" or "under developed" or underdeveloped or "middle income" or "low* income" or underserved or underserved or deprived or poor*) AND (countr* or nation* or population* or world or state*) Filters: Publication date from 2013/01/01; Field: Title/Abstract |
| [#38](https://www.ncbi.nlm.nih.gov/pubmed/advanced) | Search (developing or "less* developed" or "under developed" or underdeveloped or "middle income" or "low* income" or underserved or underserved or deprived or poor*) AND (economy or economies) Filters: Publication date from 2013/01/01; Field: Title/Abstract |
| [#39](https://www.ncbi.nlm.nih.gov/pubmed/advanced) | Search low* AND (gdp or gnp or "gross domestic" or "gross national") Filters: Publication date from 2013/01/01; Field: Title/Abstract |
| [#43](https://www.ncbi.nlm.nih.gov/pubmed/advanced) | Search "Developing Countries"[Mesh] Filters: Publication date from 2013/01/01; Field: Title/Abstract |
| [#41](https://www.ncbi.nlm.nih.gov/pubmed/advanced) | Search "transitional countr*" Filters: Publication date from 2013/01/01; Field: Title/Abstract |
| [#40](https://www.ncbi.nlm.nih.gov/pubmed/advanced) | Search lmic or lmics or "third world" or "lami countr*" Filters: Publication date from 2013/01/01; Field: Title/Abstract |
| [#30](https://www.ncbi.nlm.nih.gov/pubmed/advanced) | Search (#25) AND #24 Filters: Publication date from 2013/01/01; Field: Title/Abstract |
| [#23](https://www.ncbi.nlm.nih.gov/pubmed/advanced) | Search (((((#22) OR #18) OR #16) OR #13) OR #11) OR ((HIV[Title/Abstract] OR hiv-1[Title/Abstract] OR hiv-2*[Title/Abstract] OR hiv1[Title/Abstract] OR hiv2[Title/Abstract] OR “hiv infect*”[Title/Abstract] OR “human immunodeficiency virus”[Title/Abstract] OR “human immune deficiency virus”[Title/Abstract] OR “human immuno-deficiency virus”[Title/Abstract] OR “human immune-deficiency virus”[Title/Abstract] OR ((human immun*) AND(deficiency virus))[Title/Abstract] OR “acquired immunodeficiency syndromes”[Title/Abstract] OR “acquired immune deficiency syndrome”[Title/Abstract] OR “acquired immuno-deficiency syndrome”[Title/Abstract] OR “acquired immune-deficiency syndrome” [Title/Abstract] OR ((acquired immun*)[Title/Abstract] AND (deficiency syndrome))[Title/Abstract] OR HIV/AIDS[Title/Abstract])) Field: Title/Abstract |
| [#12](https://www.ncbi.nlm.nih.gov/pubmed/advanced) | Search ART Field: Title/Abstract |
| [#10](https://www.ncbi.nlm.nih.gov/pubmed/advanced) | Search antiretroviral* |
| [#9](https://www.ncbi.nlm.nih.gov/pubmed/advanced) | Search (HIV[Title/Abstract] OR hiv-1[Title/Abstract] OR hiv-2*[Title/Abstract] OR hiv1[Title/Abstract] OR hiv2[Title/Abstract] OR “hiv infect*”[Title/Abstract] OR “human immunodeficiency virus”[Title/Abstract] OR “human immune deficiency virus”[Title/Abstract] OR “human immuno-deficiency virus”[Title/Abstract] OR “human immune-deficiency virus”[Title/Abstract] OR ((human immun*) AND(deficiency virus))[Title/Abstract] OR “acquired immunodeficiency syndromes”[Title/Abstract] OR “acquired immune deficiency syndrome”[Title/Abstract] OR “acquired immuno-deficiency syndrome”[Title/Abstract] OR “acquired immune-deficiency syndrome” [Title/Abstract] OR ((acquired immun*)[Title/Abstract] AND (deficiency syndrome))[Title/Abstract] OR HIV/AIDS[Title/Abstract]) |

**Database: Embase <1996 to 2016 Week 48>**

**Search Strategy:**

**--------------------------------------------------------------------------------**

1 HIV infection.mp. or HIV Infections/ (60167)

2 hiv.mp. or Human immunodeficiency virus/ (296287)

3 aids.mp. or acquired immune deficiency syndrome/ (139518)

4 highly active antiretroviral therapy/ or antiretrovirals.mp. or antiretrovirus agent/ (62191)

5 1 or 2 or 3 or 4 (361447)

6 (hiv-1 or hiv-2* or hiv1 or "hiv2 acquirered immunodeficiency syndromes" or "acquired immune deficiency syndrome" or "acquired immuno-deficiency syndrome" or "acquired immune-deficiency syndrome" or HIV AIDS).ab. (87425)

7 (hiv-1 or hiv-2* or hiv1 or "hiv2 acquirered immunodeficiency syndromes" or "acquired immune deficiency syndrome" or "acquired immuno-deficiency syndrome" or "acquired immune-deficiency syndrome" or HIV AIDS).ti. (50072)

8 5 or 6 or 7 (361774)

9 limit 8 to yr="2013 -Current" (98317)

10 (Retention or retain* or "lost to follow-up" or LTFU or "loss-to-follow-up" or attrition or "loss to care" or "loss to program*" or default* or engage* or disengage* or "retention in care" or "lost to retention" or "treatment initiation" or link or "link to care" or "link to treatment" or "linkage to treatment" or "link into care" or "linkage into treatment").ti. (57330)

11 (Retention or retain* or "lost to follow-up" or LTFU or "loss-to-follow-up" or attrition or "loss to care" or "loss to program*" or default* or engage* or disengage* or "retention in care" or "lost to retention" or "treatment initiation" or link or "link to care" or "link to treatment" or "linkage to treatment" or "link into care" or "linkage into treatment").ab. (560092)

12 (Adherence or adher* or compliance or complian* or comply or complied or noncomplian* or non-complian* or non-adher* or nonadher*).ab. or (Adherence or adher* or compliance or complian* or comply or complied or noncomplian* or non-complian* or non-adher* or nonadher*).ti. (302214)

13 medication adherence.mp. or Patient Compliance/ or Medication Adherence/ (113944)

14 10 or 11 or 12 or 13 (913504)

15 9 and 14 (13350)

16 "Developing Countries".mp. or developing country/ (79257)

17 (Africa or Asia or Caribbean or "West Indies" or "South America" or "Latin America" or "Central America").ab. or (Africa or Asia or Caribbean or "West Indies" or "South America" or "Latin America" or "Central America").ti. (155481)

18 (Afghanistan or Albania or Algeria or Angola or Antigua or Barbuda or Argentina or Armenia or Armenian or Azerbaijan or Bangladesh or Barbados or Benin or Byelarus or Byelorussian or Belarus or Belorussian or Belorussia or Belize or Bhutan or Bolivia or Bosnia or Herzegovina or Hercegovina or Botswana or Brazil or Bulgaria or Burkina Faso or "Burkina Fasso " or "Upper Volta" or Burundi or Urundi or Cambodia or "Khmer Republic" or Kampuchea or Cameroon or Cameroons or Cameron or Camerons or "Cape Verde" or "Central African Republic").ab. or (Afghanistan or Albania or Algeria or Angola or Antigua or Barbuda or Argentina or Armenia or Armenian or Azerbaijan or Bangladesh or Barbados or Benin or Byelarus or Byelorussian or Belarus or Belorussian or Belorussia or Belize or Bhutan or Bolivia or Bosnia or Herzegovina or Hercegovina or Botswana or Brazil or Bulgaria or Burkina Faso or "Burkina Fasso " or "Upper Volta" or Burundi or Urundi or Cambodia or "Khmer Republic" or Kampuchea or Cameroon or Cameroons or Cameron or Camerons or "Cape Verde" or "Central African Republic").af. (619145)

19 (Chad or Chile or China or Colombia or Comoros or "Comoro Islands" or Comores or Mayotte or Congo or Zaire or "Costa Rica" or "Cote d’Ivoire" or "Ivory Coast" or Croatia or Cuba or Djibouti or "French Somaliland" or Dominica or "Dominican Republic" or "East Timor" or "East Timur" or "Timor Leste" or Ecuador or Egypt or "United Arab Republic" or "El Salvador").mp. [mp=title, abstract, heading word, drug trade name, original title, device manufacturer, drug manufacturer, device trade name, keyword, floating subheading] (272542)

20 (Eritrea or Ethiopia or Fiji or Gabon or Gabonese Republic or Gambia or Gaza or Georgia Republic or Georgian Republic or Ghana or Gold Coast or Grenada or Guatemala or Guinea or Guam or Guiana or Guyana or Haiti or Honduras or India or Maldives or Indonesia or Iran or Iraq or Jamaica or Jordan or Kazakhstan or Kazakh or Kenya or Kiribati or Korea or Kosovo or Kyrgyzstan or Kirghizia or Kyrgyz Republic or Kirghiz or Kirgizstan or Lao PDR or Laos or Latvia or Lebanon or Lesotho or Basutoland or Liberia or Libya or Lithuania or Macedonia or Madagascar or Malagasy Republic or Malaysia or Malaya or Malay or Sabah or Sarawak or Malawi).mp. [mp=title, abstract, heading word, drug trade name, original title, device manufacturer, drug manufacturer, device trade name, keyword, floating subheading] (392153)

21 (Nyasaland or Mali or Marshall Islands or Mauritania or Mauritius or Agalega Islands or Mexico or Micronesia or MiddleEast or Moldova or Moldovia or Moldovian or Mongolia or Montenegro or Morocco or Ifni or Mozambique or Myanmar or Myanma or Burma or Namibia or Nepal or Netherlands Antilles or New Caledonia or Nicaragua or Niger or Nigeria or Northern Mariana Islands or Oman or Muscat or Pakistan or Palau or Palestine or Panama or Paraguay or Peru or Philippines or Philipines or Phillipines or Phillippines or Papua New Guinea or Portugal or Romania or Rumania or Roumania or Russia or Russian or Rwanda or Ruanda).mp. [mp=title, abstract, heading word, drug trade name, original title, device manufacturer, drug manufacturer, device trade name, keyword, floating subheading] (227856)

22 (Saint Lucia or St Lucia or Saint Vincent or St Vincent or Grenadines or Samoa or Samoan Islands or Navigator Island or Navigator Islands or Sao Tome or Senegal or Serbia or Montenegro or Seychelles or Sierra Leone or Sri Lanka or Ceylon or Solomon Islands or Somalia or Sudan or Suriname or Surinam or Swaziland or South Africa or Syria or Tajikistan or Tadzhikistan or Tadjikistan or Tadzhik or Tanzania or Thailand or Togo or Togolese Republic or Tonga or Trinidad or Tobago or Tunisia or Turkey or Turkmenistan or Turkmen or Uganda or Ukraine or Uruguay or USSR or Soviet Union or Union of Soviet Socialist Republics or Uzbekistan or Uzbek or Vanuatu or NewHebrides or Venezuela or Vietnam or Viet Nam or West Bank or Yemen or Yugoslavia or Zambia or Zimbabwe).mp. [mp=title, abstract, heading word, drug trade name, original title, device manufacturer, drug manufacturer, device trade name, keyword, floating subheading] (212780)

23 ((developing or less* developed or under developed or underdeveloped or middle income or low* income or underserved or underserved or deprived or poor*) adj (countr* or nation? or population? or world or state*)).ab. or ((developing or less* developed or under developed or underdeveloped or middle income or low* income or underserved or underserved or deprived or poor*) adj (countr* or nation? or population? or world or state*)).ti. (79278)

24 ((developing or less* developed or under developed or underdeveloped or middle income or low* income) adj (economy or economies)).ab. or ((developing or less* developed or under developed or underdeveloped or middle income or low* income) adj (economy or economies)).ti. (449)

25 (low* adj (gdp or gnp or gross domestic or gross national)).ab. or (low* adj (gdp or gnp or gross domestic or gross national)).ti. (260)

26 (lmic or lmics or third world or lami countr*).ab. or (lmic or lmics or third world or lami countr*).ti. (3465)

27 transitional countr*.ab. or transitional countr*.ti. (180)

28 16 or 17 or 18 or 19 or 20 or 21 or 22 or 23 or 24 or 25 or 26 or 27 (1707524)

29 15 and 28 (4923)

30 qualitative research.mp. or qualitative research/ (52405)

31 (qualitative or ethno* or emic or etic or phenomenology* or hermeneutic* or heidegger* or husserl* or colaizzi* or giorgi* or glaser or strauss or van kaam* or van manen or "constant compar*").ab. or (qualitative or ethno* or emic or etic or phenomenology* or hermeneutic* or heidegger* or husserl* or colaizzi* or giorgi* or glaser or strauss or van kaam* or van manen or "constant compar*").ti. (193884)

32 focus group.mp. (18855)

33 exp interview/ (231016)

34 questionnaire.mp. or questionnaire/ (604527)

35 self report.mp. or self report/ (101952)

36 (focus group* or grounded theory or narrative analys* or lived experience* or life experience* or theoretical sampl* or purposive).ab. or (focus group* or grounded theory or narrative analys* or lived experience* or life experience* or theoretical sampl* or purposive).ti. (57305)

37 (ricoeur or spiegelberg* or merleau or metasynthes* or meta-synthes* or metasummar* or meta-summar* or metastud*).ab. or (ricoeur or spiegelberg* or merleau or metasynthes* or meta-synthes* or metasummar* or meta-summar* or metastud*).ti. (1103)

38 ("maximum variation" or snowball*).ab. or ("maximum variation" or snowball*).ti. (2751)

39 ((thematic* adj analys*) or content analy* or field note* or fieldnote* or field record* or field stud* or (participant* adj observ*) or (nonparticipant* adj observ*) or (non participant* adj observ*)).ab. or ((thematic* adj analys*) or content analy* or field note* or fieldnote* or field record* or field stud* or (participant* adj observ*) or (nonparticipant* adj observ*) or (non participant* adj observ*)).ti. (45181)

40 (semi-structured or semistructured or structured categor* or unstructured categor* or action research or (audiorecord* or taperecord*or videorecord* or videotap*) or ((audio or tape or video*) adj5 record*) or interview* or quasi-experiment* or (case adj stud*)).ab. or (semi-structured or semistructured or structured categor* or unstructured categor* or action research or (audiorecord* or taperecord*or videorecord* or videotap*) or ((audio or tape or video*) adj5 record*) or interview* or quasi-experiment* or (case adj stud*)).ti. (397712)

41 (collaborat* or consultat* or experience or involve* or narrative* or opinion* or participat* or partner* or perspective* or story or stories or "social science*" or view* or voice*).ab. or (collaborat* or consultat* or experience or involve* or narrative* or opinion* or participat* or partner* or perspective* or story or stories or "social science*" or view* or voice*).ti. (3246384)

42 cultural anthropology/ (39465)

43 30 or 31 or 32 or 33 or 34 or 35 or 36 or 37 or 38 or 39 or 40 or 41 or 42 (4028662)

44 29 and 43 (2622)

45 limit 44 to yr="2013 -Current" (2622)

**Database: Ovid MEDLINE(R) In-Process & Other Non-Indexed Citations and Ovid MEDLINE(R) <1946 to Present>**

Search Strategy:

--------------------------------------------------------------------------------

1 HIV infection.mp. or HIV Infections/ (211731)

2 hiv.mp. or Human immunodeficiency virus/ (348982)

3 aids.mp. or acquired immune deficiency syndrome/ (214388)

4 highly active antiretroviral therapy/ or antiretrovirals.mp. or antiretrovirus agent/ (24469)

5 1 or 2 or 3 or 4 (444483)

6 (hiv-1 or hiv-2* or hiv1 or "hiv2 acquirered immunodeficiency syndromes" or "acquired immune deficiency syndrome" or "acquired immuno-deficiency syndrome" or "acquired immune-deficiency syndrome" or HIV AIDS).ab. (101987)

7 (hiv-1 or hiv-2* or hiv1 or "hiv2 acquirered immunodeficiency syndromes" or "acquired immune deficiency syndrome" or "acquired immuno-deficiency syndrome" or "acquired immune-deficiency syndrome" or HIV AIDS).ti. (57945)

8 5 or 6 or 7 (445014)

9 limit 8 to yr="2013 -Current" (79356)

10 (Retention or retain* or "lost to follow-up" or LTFU or "loss-to-follow-up" or attrition or "loss to care" or "loss to program*" or default* or engage* or disengage* or "retention in care" or "lost to retention" or "treatment initiation" or link or "link to care" or "link to treatment" or "linkage to treatment" or "link into care" or "linkage into treatment").ti. (73512)

11 (Retention or retain* or "lost to follow-up" or LTFU or "loss-to-follow-up" or attrition or "loss to care" or "loss to program*" or default* or engage* or disengage* or "retention in care" or "lost to retention" or "treatment initiation" or link or "link to care" or "link to treatment" or "linkage to treatment" or "link into care" or "linkage into treatment").ab. (600596)

12 (Adherence or adher* or compliance or complian* or comply or complied or noncomplian* or non-complian* or non-adher* or nonadher*).ab. or (Adherence or adher* or compliance or complian* or comply or complied or noncomplian* or non-complian* or non-adher* or nonadher*).ti. (280196)

13 medication adherence.mp. or Patient Compliance/ or Medication Adherence/ (72305)

14 10 or 11 or 12 or 13 (930826)

15 9 and 14 (11064)

16 "Developing Countries".mp. or developing country/ (111816)

17 (Africa or Asia or Caribbean or "West Indies" or "South America" or "Latin America" or "Central America").ab. or (Africa or Asia or Caribbean or "West Indies" or "South America" or "Latin America" or "Central America").ti. (153218)

18 (Afghanistan or Albania or Algeria or Angola or Antigua or Barbuda or Argentina or Armenia or Armenian or Azerbaijan or Bangladesh or Barbados or Benin or Byelarus or Byelorussian or Belarus or Belorussian or Belorussia or Belize or Bhutan or Bolivia or Bosnia or Herzegovina or Hercegovina or Botswana or Brazil or Bulgaria or Burkina Faso or "Burkina Fasso " or "Upper Volta" or Burundi or Urundi or Cambodia or "Khmer Republic" or Kampuchea or Cameroon or Cameroons or Cameron or Camerons or "Cape Verde" or "Central African Republic").ab. or (Afghanistan or Albania or Algeria or Angola or Antigua or Barbuda or Argentina or Armenia or Armenian or Azerbaijan or Bangladesh or Barbados or Benin or Byelarus or Byelorussian or Belarus or Belorussian or Belorussia or Belize or Bhutan or Bolivia or Bosnia or Herzegovina or Hercegovina or Botswana or Brazil or Bulgaria or Burkina Faso or "Burkina Fasso " or "Upper Volta" or Burundi or Urundi or Cambodia or "Khmer Republic" or Kampuchea or Cameroon or Cameroons or Cameron or Camerons or "Cape Verde" or "Central African Republic").af. (588665)

19 (Chad or Chile or China or Colombia or Comoros or "Comoro Islands" or Comores or Mayotte or Congo or Zaire or "Costa Rica" or "Cote d’Ivoire" or "Ivory Coast" or Croatia or Cuba or Djibouti or "French Somaliland" or Dominica or "Dominican Republic" or "East Timor" or "East Timur" or "Timor Leste" or Ecuador or Egypt or "United Arab Republic" or "El Salvador").mp. [mp=title, abstract, original title, name of substance word, subject heading word, keyword heading word, protocol supplementary concept word, rare disease supplementary concept word, unique identifier] (263115)

20 (Eritrea or Ethiopia or Fiji or Gabon or Gabonese Republic or Gambia or Gaza or Georgia Republic or Georgian Republic or Ghana or Gold Coast or Grenada or Guatemala or Guinea or Guam or Guiana or Guyana or Haiti or Honduras or India or Maldives or Indonesia or Iran or Iraq or Jamaica or Jordan or Kazakhstan or Kazakh or Kenya or Kiribati or Korea or Kosovo or Kyrgyzstan or Kirghizia or Kyrgyz Republic or Kirghiz or Kirgizstan or Lao PDR or Laos or Latvia or Lebanon or Lesotho or Basutoland or Liberia or Libya or Lithuania or Macedonia or Madagascar or Malagasy Republic or Malaysia or Malaya or Malay or Sabah or Sarawak or Malawi).mp. [mp=title, abstract, original title, name of substance word, subject heading word, keyword heading word, protocol supplementary concept word, rare disease supplementary concept word, unique identifier] (495513)

21 (Nyasaland or Mali or Marshall Islands or Mauritania or Mauritius or Agalega Islands or Mexico or Micronesia or MiddleEast or Moldova or Moldovia or Moldovian or Mongolia or Montenegro or Morocco or Ifni or Mozambique or Myanmar or Myanma or Burma or Namibia or Nepal or Netherlands Antilles or New Caledonia or Nicaragua or Niger or Nigeria or Northern Mariana Islands or Oman or Muscat or Pakistan or Palau or Palestine or Panama or Paraguay or Peru or Philippines or Philipines or Phillipines or Phillippines or Papua New Guinea or Portugal or Romania or Rumania or Roumania or Russia or Russian or Rwanda or Ruanda).mp. [mp=title, abstract, original title, name of substance word, subject heading word, keyword heading word, protocol supplementary concept word, rare disease supplementary concept word, unique identifier] (272544)

22 (Saint Lucia or St Lucia or Saint Vincent or St Vincent or Grenadines or Samoa or Samoan Islands or Navigator Island or Navigator Islands or Sao Tome or Senegal or Serbia or Montenegro or Seychelles or Sierra Leone or Sri Lanka or Ceylon or Solomon Islands or Somalia or Sudan or Suriname or Surinam or Swaziland or South Africa or Syria or Tajikistan or Tadzhikistan or Tadjikistan or Tadzhik or Tanzania or Thailand or Togo or Togolese Republic or Tonga or Trinidad or Tobago or Tunisia or Turkey or Turkmenistan or Turkmen or Uganda or Ukraine or Uruguay or USSR or Soviet Union or Union of Soviet Socialist Republics or Uzbekistan or Uzbek or Vanuatu or NewHebrides or Venezuela or Vietnam or Viet Nam or West Bank or Yemen or Yugoslavia or Zambia or Zimbabwe).mp. [mp=title, abstract, original title, name of substance word, subject heading word, keyword heading word, protocol supplementary concept word, rare disease supplementary concept word, unique identifier] (316774)

23 ((developing or less* developed or under developed or underdeveloped or middle income or low* income or underserved or underserved or deprived or poor*) adj (countr* or nation? or population? or world or state*)).ab. or ((developing or less* developed or under developed or underdeveloped or middle income or low* income or underserved or underserved or deprived or poor*) adj (countr* or nation? or population? or world or state*)).ti. (78710)

24 ((developing or less* developed or under developed or underdeveloped or middle income or low* income) adj (economy or economies)).ab. or ((developing or less* developed or under developed or underdeveloped or middle income or low* income) adj (economy or economies)).ti. (387)

25 (low* adj (gdp or gnp or gross domestic or gross national)).ab. or (low* adj (gdp or gnp or gross domestic or gross national)).ti. (207)

26 (lmic or lmics or third world or lami countr*).ab. or (lmic or lmics or third world or lami countr*).ti. (4740)

27 transitional countr*.ab. or transitional countr*.ti. (146)

28 16 or 17 or 18 or 19 or 20 or 21 or 22 or 23 or 24 or 25 or 26 or 27 (1920955)

29 15 and 28 (4586)

30 qualitative research.mp. or qualitative research/ (41912)

31 (qualitative or ethno* or emic or etic or phenomenology* or hermeneutic* or heidegger* or husserl* or colaizzi* or giorgi* or glaser or strauss or van kaam* or van manen or "constant compar*").ab. or (qualitative or ethno* or emic or etic or phenomenology* or hermeneutic* or heidegger* or husserl* or colaizzi* or giorgi* or glaser or strauss or van kaam* or van manen or "constant compar*").ti. (199426)

32 focus group.mp. (17435)

33 exp interview/ (30046)

34 questionnaire.mp. or questionnaire/ (556292)

35 self report.mp. or self report/ (59012)

36 (focus group* or grounded theory or narrative analys* or lived experience* or life experience* or theoretical sampl* or purposive).ab. or (focus group* or grounded theory or narrative analys* or lived experience* or life experience* or theoretical sampl* or purposive).ti. (53725)

37 (ricoeur or spiegelberg* or merleau or metasynthes* or meta-synthes* or metasummar* or meta-summar* or metastud*).ab. or (ricoeur or spiegelberg* or merleau or metasynthes* or meta-synthes* or metasummar* or meta-summar* or metastud*).ti. (1218)

38 ("maximum variation" or snowball*).ab. or ("maximum variation" or snowball*).ti. (2544)

39 ((thematic* adj analys*) or content analy* or field note* or fieldnote* or field record* or field stud* or (participant* adj observ*) or (nonparticipant* adj observ*) or (non participant* adj observ*)).ab. or ((thematic* adj analys*) or content analy* or field note* or fieldnote* or field record* or field stud* or (participant* adj observ*) or (nonparticipant* adj observ*) or (non participant* adj observ*)).ti. (47423)

40 (semi-structured or semistructured or structured categor* or unstructured categor* or action research or (audiorecord* or taperecord*or videorecord* or videotap*) or ((audio or tape or video*) adj5 record*) or interview* or quasi-experiment* or (case adj stud*)).ab. or (semi-structured or semistructured or structured categor* or unstructured categor* or action research or (audiorecord* or taperecord*or videorecord* or videotap*) or ((audio or tape or video*) adj5 record*) or interview* or quasi-experiment* or (case adj stud*)).ti. (401573)

41 (collaborat* or consultat* or experience or involve* or narrative* or opinion* or participat* or partner* or perspective* or story or stories or "social science*" or view* or voice*).ab. or (collaborat* or consultat* or experience or involve* or narrative* or opinion* or participat* or partner* or perspective* or story or stories or "social science*" or view* or voice*).ti. (3433712)

42 cultural anthropology/ (5845)

43 30 or 31 or 32 or 33 or 34 or 35 or 36 or 37 or 38 or 39 or 40 or 41 or 42 (4169676)

44 29 and 43 (2570)

45 limit 44 to yr="2013 -Current" (2570)

Cinahl

| **#** | **Query** |
| --- | --- |
| S35 | Limit S34 to 2013-present |
| S34 | S22 AND S33 |
| S33 | S23 OR S24 OR S25 OR S26 OR S27 OR S28 OR S29 OR S30 OR S31 OR S32 |
| S32 | (MM "Anthropology, Cultural") |
| S31 | TX collaborat* or consultat* or experience or involve* or narrative* or opinion* or participat* or partner* or perspective* or story or stories or "social science*" or view* or voice*) |
| S30 | TX semi-structured or semistructured or structured categor* or unstructured categor* or action research or (audiorecord* or taperecord*or videorecord* or videotap*) or ((audio or tape or video*) adj5 record*) or interview* or quasi-experiment* or (case adj stud*) |
| S29 | TX ((thematic* adj analys*) or content analy* or field note* or fieldnote* or field record* or field stud* or (participant* adj observ*) or (nonparticipant* adj observ*) or (non participant* adj observ*) |
| S28 | TX "maximum variation" or snowball* |
| S27 | TX ricoeur or spiegelberg* or merleau or metasynthes* or meta-synthes* or metasummar* or meta-summar* or metastud* |
| S26 | TX (focus group* or grounded theory or narrative analys* or lived experience* or life experience* or theoretical sampl* or purposive |
| S25 | (MH "Structured Questionnaires") OR (MH "Interviews") |
| S24 | (MM "Self Report") |
| S23 | TX "qualitative research" OR TX ( (qualitative or ethno* or emic or etic or phenomenology* or hermeneutic* or heidegger* or husserl* or colaizzi* or giorgi* or glaser or strauss or van kaam* or van manen or "constant compar*" ) OR TX "focus ghroup*" |
| S22 | S8 AND S21 |
| S21 | S9 OR S10 OR S11 OR S12 OR S13 OR S14 OR S15 OR S16 OR S17 OR S18 OR S19 OR S20 |
| S20 | TX ( lmic or lmics or third world or lami countr* ) OR TX transitional countr* |
| S19 | TX low* and (gdp or gnp or gross domestic or gross national) |
| S18 | TX (developing or less* developed or under developed or underdeveloped or middle income or low* income) and (economy or economies) |
| S17 | AB ((developing or less* developed or under developed or underdeveloped or middle income or low* income or underserved or underserved or deprived or poor*) AND (countr* or nation* or population* or world or state*) |
| S16 | TX ((developing or less* developed or under developed or underdeveloped or middle income or low* income or underserved or underserved or deprived or poor*) adj (countr* or nation? or population? or world or state*)) |
| S15 | TX Saint Lucia or St Lucia or Saint Vincent or St Vincent or Grenadines or Samoa or Samoan Islands or Navigator Island or Navigator Islands or Sao Tome or Senegal or Serbia or Montenegro or Seychelles or Sierra Leone or Sri Lanka or Ceylon or Solomon Islands or Somalia or Sudan or Suriname or Surinam or Swaziland or South Africa or Syria or Tajikistan or Tadzhikistan or Tadjikistan or Tadzhik or Tanzania or Thailand or Togo or Togolese Republic or Tonga or Trinidad or Tobago or Tunisia or Turkey or Turkmenistan or Turkmen or Uganda or Ukraine or Uruguay or USSR or Soviet Union or Union of Soviet Socialist Republics or Uzbekistan or Uzbek or Vanuatu or NewHebrides or Venezuela or Vietnam or Viet Nam or West Bank or Yemen or Yugoslavia or Zambia or Zimbabwe |
| S14 | TX Nyasaland or Mali or Marshall Islands or Mauritania or Mauritius or Agalega Islands or Mexico or Micronesia or MiddleEast or Moldova or Moldovia or Moldovian or Mongolia or Montenegro or Morocco or Ifni or Mozambique or Myanmar or Myanma or Burma or Namibia or Nepal or Netherlands Antilles or New Caledonia or Nicaragua or Niger or Nigeria or Northern Mariana Islands or Oman or Muscat or Pakistan or Palau or Palestine or Panama or Paraguay or Peru or Philippines or Philipines or Phillipines or Phillippines or Papua New Guinea or Portugal or Romania or Rumania or Roumania or Russia or Russian or Rwanda or Ruanda |
| S13 | TX Afghanistan or Albania or Algeria or Angola or Antigua or Barbuda or Argentina or Armenia or Armenian or Azerbaijan or Bangladesh or Barbados or Benin or Byelarus or Byelorussian or Belarus or Belorussian or Belorussia or Belize or Bhutan or Bolivia or Bosnia or Herzegovina or Hercegovina or Botswana or Brazil or Bulgaria or Burkina Faso or "Burkina Fasso " or "Upper Volta" or Burundi or Urundi or Cambodia or "Khmer Republic" or Kampuchea or Cameroon or Cameroons or Cameron or Camerons or "Cape Verde" or "Central African Republic" |
| S12 | TX Chad or Chile or China or Colombia or Comoros or "Comoro Islands" or Comores or Mayotte or Congo or Zaire or "Costa Rica" or "Cote d’Ivoire" or "Ivory Coast" or Croatia or Cuba or Djibouti or "French Somaliland" or Dominica or "Dominican Republic" or "East Timor" or "East Timur" or "Timor Leste" or Ecuador or Egypt or "United Arab Republic" or "El Salvador" |
| S11 | TX Eritrea or Ethiopia or Fiji or Gabon or Gabonese Republic or Gambia or Gaza or Georgia Republic or Georgian Republic or Ghana or Gold Coast or Grenada or Guatemala or Guinea or Guam or Guiana or Guyana or Haiti or Honduras or India or Maldives or Indonesia or Iran or Iraq or Jamaica or Jordan or Kazakhstan or Kazakh or Kenya or Kiribati or Korea or Kosovo or Kyrgyzstan or Kirghizia or Kyrgyz Republic or Kirghiz or Kirgizstan or Lao PDR or Laos or Latvia or Lebanon or Lesotho or Basutoland or Liberia or Libya or Lithuania or Macedonia or Madagascar or Malagasy Republic or Malaysia or Malaya or Malay or Sabah or Sarawak or Malawi |
| S10 | TI ( Africa or Asia or Caribbean or "West Indies" or "South America" or "Latin America" or "Central America" ) OR AB ( Africa or Asia or Caribbean or "West Indies" or "South America" or "Latin America" or "Central America" ) |
| S9 | MH developing countries OR TI developing countries OR AB developing countries |
| S8 | S3 AND S7 |
| S7 | S4 OR S5 OR S6 |
| S6 | MH medication adherence OR MH patient compliance |
| S5 | TI ( Adherence or adher* or compliance or complian* or comply or complied or noncomplian* or non-complian* or non-adher* or nonadher*).ab. or (Adherence or adher* or compliance or complian* or comply or complied or noncomplian* or non-complian* or non-adher* or nonadher* ) OR AB ( Adherence or adher* or compliance or complian* or comply or complied or noncomplian* or non-complian* or non-adher* or nonadher*).ab. or (Adherence or adher* or compliance or complian* or comply or complied or noncomplian* or non-complian* or non-adher* or nonadher* ) |
| S4 | AB ( (Retention or retain* or "lost to follow-up" or LTFU or "loss-to-follow-up" or attrition or "loss to care" or "loss to program*" or default* or engage* or disengage* or "retention in care" or "lost to retention" or "treatment initiation" or link or "link to care" or "link to treatment" or "linkage to treatment" or "link into care" or "linkage into treatment" ) OR TI ( (Retention or retain* or "lost to follow-up" or LTFU or "loss-to-follow-up" or attrition or "loss to care" or "loss to program*" or default* or engage* or disengage* or "retention in care" or "lost to retention" or "treatment initiation" or link or "link to care" or "link to treatment" or "linkage to treatment" or "link into care" or "linkage into treatment" ) |
| S3 | S1 OR S2 |
| S2 | AB HIV* OR AB AIDS OR AB acquired immunodeficiency syndrome OR AB antiretroviral therapy |
| S1 | TI HIV* OR TI aids OR TI acquired immune deficiency syndrome OR TI antiretroviral therapy |

**PsycInfo**

| **#** | **Query** | **Action** |
| --- | --- | --- |
| S32 | Limit S31 to 2013-present |  |
| S31 | S21 AND S30 |  |
| S30 | S22 OR S23 OR S24 OR S25 OR S26 OR S27 OR S28 OR S29 |  |
| S29 | TX collaborat* or consultat* or experience or involve* or narrative* or opinion* or participat* or partner* or perspective* or story or stories or "social science*" or view* or voice*) |  |
| S28 | TX semi-structured or semistructured or structured categor* or unstructured categor* or action research or (audiorecord* or taperecord*or videorecord* or videotap*) or ((audio or tape or video*) adj5 record*) or interview* or quasi-experiment* or (case adj stud*) |  |
| S27 | TX ((thematic* adj analys*) or content analy* or field note* or fieldnote* or field record* or field stud* or (participant* adj observ*) or (nonparticipant* adj observ*) or (non participant* adj observ*) |  |
| S26 | TX "maximum variation" or snowball* |  |
| S25 | TX ricoeur or spiegelberg* or merleau or metasynthes* or meta-synthes* or metasummar* or meta-summar* or metastud* |  |
| S24 | TX (focus group* or grounded theory or narrative analys* or lived experience* or life experience* or theoretical sampl* or purposive |  |
| S23 | (MH "Structured Questionnaires") OR (MH "Interviews") |  |
| S22 | TX "qualitative research" OR TX ( (qualitative or ethno* or emic or etic or phenomenology* or hermeneutic* or heidegger* or husserl* or colaizzi* or giorgi* or glaser or strauss or van kaam* or van manen or "constant compar*" ) OR TX "focus ghroup*" |  |
| S21 | S8 AND S20 |  |
| S20 | S9 OR S10 OR S11 OR S12 OR S13 OR S14 OR S15 OR S16 OR S17 OR S18 OR S19 |  |
| S19 | TX ( lmic or lmics or third world or lami countr* ) OR TX transitional countr* |  |
| S18 | TX low* and (gdp or gnp or gross domestic or gross national) |  |
| S17 | TX (developing or less* developed or under developed or underdeveloped or middle income or low* income) and (economy or economies) |  |
| S16 | AB ((developing or less* developed or under developed or underdeveloped or middle income or low* income or underserved or underserved or deprived or poor*) AND (countr* or nation* or population* or world or state*) |  |
| S15 | TX Saint Lucia or St Lucia or Saint Vincent or St Vincent or Grenadines or Samoa or Samoan Islands or Navigator Island or Navigator Islands or Sao Tome or Senegal or Serbia or Montenegro or Seychelles or Sierra Leone or Sri Lanka or Ceylon or Solomon Islands or Somalia or Sudan or Suriname or Surinam or Swaziland or South Africa or Syria or Tajikistan or Tadzhikistan or Tadjikistan or Tadzhik or Tanzania or Thailand or Togo or Togolese Republic or Tonga or Trinidad or Tobago or Tunisia or Turkey or Turkmenistan or Turkmen or Uganda or Ukraine or Uruguay or USSR or Soviet Union or Union of Soviet Socialist Republics or Uzbekistan or Uzbek or Vanuatu or NewHebrides or Venezuela or Vietnam or Viet Nam or West Bank or Yemen or Yugoslavia or Zambia or Zimbabwe |  |
| S14 | TX Nyasaland or Mali or Marshall Islands or Mauritania or Mauritius or Agalega Islands or Mexico or Micronesia or MiddleEast or Moldova or Moldovia or Moldovian or Mongolia or Montenegro or Morocco or Ifni or Mozambique or Myanmar or Myanma or Burma or Namibia or Nepal or Netherlands Antilles or New Caledonia or Nicaragua or Niger or Nigeria or Northern Mariana Islands or Oman or Muscat or Pakistan or Palau or Palestine or Panama or Paraguay or Peru or Philippines or Philipines or Phillipines or Phillippines or Papua New Guinea or Portugal or Romania or Rumania or Roumania or Russia or Russian or Rwanda or Ruanda |  |
| S13 | TX Afghanistan or Albania or Algeria or Angola or Antigua or Barbuda or Argentina or Armenia or Armenian or Azerbaijan or Bangladesh or Barbados or Benin or Byelarus or Byelorussian or Belarus or Belorussian or Belorussia or Belize or Bhutan or Bolivia or Bosnia or Herzegovina or Hercegovina or Botswana or Brazil or Bulgaria or Burkina Faso or "Burkina Fasso " or "Upper Volta" or Burundi or Urundi or Cambodia or "Khmer Republic" or Kampuchea or Cameroon or Cameroons or Cameron or Camerons or "Cape Verde" or "Central African Republic" |  |
| S12 | TX Chad or Chile or China or Colombia or Comoros or "Comoro Islands" or Comores or Mayotte or Congo or Zaire or "Costa Rica" or "Cote d’Ivoire" or "Ivory Coast" or Croatia or Cuba or Djibouti or "French Somaliland" or Dominica or "Dominican Republic" or "East Timor" or "East Timur" or "Timor Leste" or Ecuador or Egypt or "United Arab Republic" or "El Salvador" |  |
| S11 | TX Eritrea or Ethiopia or Fiji or Gabon or Gabonese Republic or Gambia or Gaza or Georgia Republic or Georgian Republic or Ghana or Gold Coast or Grenada or Guatemala or Guinea or Guam or Guiana or Guyana or Haiti or Honduras or India or Maldives or Indonesia or Iran or Iraq or Jamaica or Jordan or Kazakhstan or Kazakh or Kenya or Kiribati or Korea or Kosovo or Kyrgyzstan or Kirghizia or Kyrgyz Republic or Kirghiz or Kirgizstan or Lao PDR or Laos or Latvia or Lebanon or Lesotho or Basutoland or Liberia or Libya or Lithuania or Macedonia or Madagascar or Malagasy Republic or Malaysia or Malaya or Malay or Sabah or Sarawak or Malawi |  |
| S10 | TI ( Africa or Asia or Caribbean or "West Indies" or "South America" or "Latin America" or "Central America" ) OR AB ( Africa or Asia or Caribbean or "West Indies" or "South America" or "Latin America" or "Central America" ) |  |
| S9 | developing countries OR TI developing countries OR AB developing countries |  |
| S8 | S3 AND S7 |  |
| S7 | S4 OR S5 OR S6 |  |
| S6 | medication adherence OR patient compliance |  |
| S5 | TI ( Adherence or adher* or compliance or complian* or comply or complied or noncomplian* or non-complian* or non-adher* or nonadher*).ab. or (Adherence or adher* or compliance or complian* or comply or complied or noncomplian* or non-complian* or non-adher* or nonadher* ) OR AB ( Adherence or adher* or compliance or complian* or comply or complied or noncomplian* or non-complian* or non-adher* or nonadher*).ab. or (Adherence or adher* or compliance or complian* or comply or complied or noncomplian* or non-complian* or non-adher* or nonadher* ) |  |
| S4 | AB ( (Retention or retain* or "lost to follow-up" or LTFU or "loss-to-follow-up" or attrition or "loss to care" or "loss to program*" or default* or engage* or disengage* or "retention in care" or "lost to retention" or "treatment initiation" or link or "link to care" or "link to treatment" or "linkage to treatment" or "link into care" or "linkage into treatment" ) OR TI ( (Retention or retain* or "lost to follow-up" or LTFU or "loss-to-follow-up" or attrition or "loss to care" or "loss to program*" or default* or engage* or disengage* or "retention in care" or "lost to retention" or "treatment initiation" or link or "link to care" or "link to treatment" or "linkage to treatment" or "link into care" or "linkage into treatment" ) |  |
| S3 | S1 OR S2 |  |
| S2 | AB HIV* OR AB AIDS OR AB acquired immunodeficiency syndrome OR AB antiretroviral therapy |  |
| S1 | TI HIV* OR TI aids OR TI acquired immune deficiency syndrome OR TI antiretroviral therapy |  |

**Database : LILACS**

Search on : HIV OR AIDS [Words] and linkage or retention or adherence or non-adherence or compliance [Words] and survey or questionnaire or qualitative or focus [Words]

**Global health library**

(tw:(hiv OR aids)) AND (tw:(survey OR questionnaire OR qualitative OR focus)) AND (tw:(adherence OR linkage OR retention OR non-adherence OR compliance))

**Proquest Dissertations and Theses**

S3 TI: (HIV and adherence and qualitative)Limits applied
