# Supplementary material for: Being HIV positive and staying on antiretroviral therapy in Africa: A qualitative systematic review and theoretical model
Source: PLoS One. 2019 Jan 10;14(1):e0210408. doi: 10.1371/journal.pone.0210408 (PMC6328200; doi:10.1371/journal.pone.0210408)
Supplement: S4 Appendix — (DOCX) [file pone.0210408.s004.docx]

**ENTREQ STATEMENT – CHECKLIST**

Enhancing transparency in reporting the synthesis of qualitative research: the ENTREQ statement: Tong et al. BMC Medical Research Methodology 2012, 12:181 (<http://www.biomedcentral.com/1471-2288/12/181> )

| **No** | **Aim** | **State the research question the synthesis addresses** | **Addressed** | **Support from manuscript** |
| --- | --- | --- | --- | --- |
| 1 | Synthesis methodology | Identify the synthesis methodology or theoretical framework which underpins the synthesis, and describe the rationale for choice of methodology (e.g. meta-ethnography, thematic synthesis, critical interpretive synthesis, grounded theory synthesis, realist synthesis, meta-aggregation, meta-study, framework synthesis). | Yes, we used a grounded theory approach to indentify new themes | In our protocol, we developed a conceptual model based on factors identified from the literature, which influence linkage, retention in care and adherence (S1 Fig) that informed our preliminary coding framework (S3 Appendix). Two authors (IEW and AR or LH) independently coded studies deductively, using the coding framework, and inductively, identifying new codes using a grounded theory approach with Atlas.ti software [32]. We used thematic synthesis to identify themes and subthemes. |
| 2 | Approach to searching | Indicate whether the search was pre-planned (comprehensive search strategies to seek all available studies) or iterative (to seek all available concepts until they theoretical saturation is achieved). | Yes, a pre-planned comprehensive search was conducted | We searched Medline, Embase, CINAHL, PsychInfo, LILACS, Global Health Library (date of last search 4 December 2016) and the Proquest Dissertation and Thesis database (9 December 2016). We limited our search to studies published from 1 January 2013 to include the most recent literature. Our search strategy included terms related to HIV, retention in care, adherence, linkage, LMICs and qualitative data collection and analysis methods. The full search strategies for each database are reported in S2 Appendix. |
| 3 | Inclusion criteria | Specify the inclusion/exclusion criteria (e.g. in terms of population, language, year limits, type of publication, study type). | Yes, inclusion and exclusion criteria were explicit | We considered qualitative studies that used ethnographies, process evaluations, case studies, and mixed-methods and used data collection techniques including observations, interviews, focus groups, and document analyses to collect qualitative data; and thematic analyses, narrative analyses, and presentations of findings to analyze qualitative data. Studies that used qualitative data collection methods but not qualitative data analysis methods were ineligible. We restricted studies to those conducted in low-and-middle-income countries (LMICs). We included studies that explored our phenomenon of interest, specifically - perspectives, perceptions, and experiences of HIV-positive people, caregivers, and providers that influenced linkage, retention, and adherence to ART. We included HIV-positive children, adolescents, and adults; healthcare workers and traditional healers who provided services to HIV-positive people, and caregivers of HIV-positive people. We excluded participants that only received ART for prevention of mother to child transmission. |
| 4 | Data sources | Describe the information sources used (e.g. electronic databases (MEDLINE, EMBASE, CINAHL, psycINFO, Econlit), grey literature databases (digital thesis, policy reports), relevant organisational websites, experts, information specialists, generic web searches (Google Scholar) hand searching, reference lists) and when the searches conducted; provide the rationale for using the data sources. | Yes, Data sources are detailed – we conducted database searches of published articles and theses | We searched Medline, Embase, CINAHL, PsychInfo, LILACS, Global Health Library (date of last search 4 December 2016) and the Proquest Dissertation and Thesis database (9 December 2016). We limited our search to studies published from 1 January 2013 to include the most recent literature. Our search strategy included terms related to HIV, retention in care, adherence, linkage, LMICs and qualitative data collection and analysis methods. |
| 5 | Electronic Search strategy | Describe the literature search (e.g. provide electronic search strategies with population terms, clinical or health topic terms, experiential or social phenomena related terms, filters for qualitative research, and search limits). | Yes, search strategies are available | The full search strategies for each database are reported in S2 Appendix. |
| 6 | Study screening methods | Describe the process of study screening and sifting (e.g. title, abstract and full text review, number of independent reviewers who screened studies). | Yes, study screening methods are detailed | After de-duplication, two review authors (IEW and AR or LH) independently screened titles and abstracts to identify potentially relevant studies using Covidence [28], resolving discrepancies through discussions and consultation of a third author. Two authors (IEW and AR or LH) independently screened all full texts to identify eligible studies and one author (IEW) extracted data on the participants, setting, phenomenon of interest and richness of data. Discrepancies in eligibility and extracted data were discussed with a third author. |
| 7 | Study characteristics | Present the characteristics of the included studies (e.g. year of publication, country, population, number of participants, data collection, methodology, analysis, research questions). | Yes, detailed included studies tables are available | A summary of the characteristics of studies included in the qualitative synthesis is presented in S2 Table. Of included studies 15 were conducted in South Africa, 9 in Uganda, 8 in Tanzania 4 in Swaziland, 4 in Zimbabwe, 3 each in Ethiopia, Kenya, Malawi, and Zambia, and 1 each in Cameroon, Lesotho, Mozambique, Nigeria, Rwanda, Kenya, Nigeria, and Tanzania. Studies examined factors influencing linkage to care, adherence to ART or retention in care amongst adults (n=22), adolescents or children (n=10), adults, adolescents and children (n=2), men (n=4), women (n=4), pregnant or postpartum women (n=7), female sex workers (n=3), people who inject drugs (PWID) (n=2), people living with disabilities (PWD) (n=2), men who have sex with men (MSM) (n=2) and refugees (n=1). Studies used a variety of methods to collect data, including in-depth interviews, focus-group discussions, ethnography, participant observation, role-play and photo-elicitation interviews, most studies used a combination of methods. In-depth interviewing was the most common method of collecting data. Study participants comprised HIV-positive as well as a few HIV negative people, caregivers of HIV-positive children and adolescents, healthcare workers, traditional leaders, lay counsellors, community health workers and other key informants. |
| 8 | Study selection results | Identify the number of studies screened and provide reasons for study exclusion (e,g, for comprehensive searching, provide numbers of studies screened and reasons for exclusion indicated in a figure/flowchart; for iterative searching describe reasons for study exclusion and inclusion based on modifications t the research question and/or contribution to theory development). | Yes, PRISMA diagram and supplementary materials are available detailing this | Our search yielded a total of 11,518 records (Figure 1). After removal of duplicates, we screened titles and abstracts of 5792 records and full texts of 397 articles. We excluded 136 full-text articles with reasons. Of the 261 eligible articles, 185 articles were not included in the sample as they were either classified as ‘thin’ studies (n=179), or were conducted in countries outside of Africa (n=6). We purposively sampled 76 articles and included 61 articles [34-95], reporting on 59 studies, in the qualitative synthesis. The remaining 15 articles were not included in the synthesis as data saturation was reached (a more detailed PRISMA flowchart is presented in S2 Fig). |
| 9 | Rationale for appraisal | Describe the rationale and approach used to appraise the included studies or selected findings (e.g. assessment of conduct (validity and robustness), assessment of reporting (transparency), assessment of content and utility of the findings). | Yes, the quality assessment is described in methods and result are presented in the paper and supplementary materials | Critical appraisal of the 61 included papers demonstrated that the studies were of high quality with the majority making a ‘fairly thorough attempt’ at increasing rigor in sampling (57; 93%) and data collection (52; 85%), and grounding the findings in the data (54; 88%). Studies scored less well on ‘rigor in analysis’, and the ‘breadth and depth of their findings’, with 41 (67%) and 40 (66%) being assessed as having made a ‘fairly thorough attempt’ respectively. This lower score for some studies was mostly due to poor reporting of analysis techniques and a lack of transformation of the data beyond lists of the main themes. Critical appraisal findings are presented in S3 Table. |
| 10 | Appraisal items | State the tools, frameworks and criteria used to appraise the studies or selected findings (e.g. Existing tools: CASP, QARI, COREQ, Mays and Pope [25]; reviewer developed tools; describe the domains assessed: research team, study design, data analysis and interpretations, reporting). | Yes, tool is described | We assessed quality of included studies using a modified version of the tool developed by the EPPI-center [33] (S1 Table). Two authors (IEW and LH) independently assessed included studies in terms of (1) rigor in sampling, (2) data collection and analysis, (3) level to which the findings were supported by the data, and (4) the breadth and depth of study findings. Discrepancies were resolved through discussion or consultation of a third author (PG or SO). |
| 11 | Appraisal process | Indicate whether the appraisal was conducted independently by more than one reviewer and if consensus was required. | Yes, appraisal was conducted independently by more than one reviewer | Two authors (IEW and LH) independently assessed included studies in terms of (1) rigor in sampling, (2) data collection and analysis, (3) level to which the findings were supported by the data, and (4) the breadth and depth of study findings. Discrepancies were resolved through discussion or consultation of a third author (PG or SO). |
| 12 | Appraisal results | Present results of the quality assessment and indicate which articles, if any, were weighted/excluded based on the assessment and give the rationale. | No studies were excluded based on the quality assessment |  |
| 13 | Data extraction | Indicate which sections of the primary studies were analysed and how were the data extracted from the primary studies? (e.g. all text under the headings “results /conclusions” were extracted electronically and entered into a computer software). | Yes, We extracted authors results and conclusions | We coded the authors results and conclusions from the primary studies. |
| 14 | Software | State the computer software used, if any. | Yes, detailed | Atlas.ti software [32]; Covidence [28] |
| 15 | Number of reviewers | Identify who was involved in coding and analysis. | Yes, Identified | Two authors (IEW and AR or LH) independently coded studies …… [32]. We discussed the codes of each study, resolved discrepancies and added new codes to the existing framework. …… This was an iterative process through frequent and regular research team meetings (IEW, AR, LH, PG and SO). We repeatedly refined our conceptual model and coding framework according to the preliminary themes. ……Two authors (IEW and AR) independently assessed these studies for inclusion, proceeded with independent coding and discussed each study to resolve discrepancies. |
| 16 | Coding | Describe the process for coding of data (e.g. line by line coding to search for concepts). | Yes, described | We conducted In Vivo coding and generated memos capturing emerging themes using Atlas.ti software [32]. |
| 17 | Study comparison | Describe how were comparisons made within and across studies (e.g. subsequent studies were coded into pre-existing concepts, and new concepts were created when deemed necessary). | Yes, detailed | We discussed the codes of each study, resolved discrepancies and added new codes to the existing framework. We coded the authors results and conclusions from the primary studies. We used thematic synthesis to identify themes and subthemes. This was an iterative process through frequent and regular research team meetings (IEW, AR, LH, PG and SO). We repeatedly refined our conceptual model and coding framework according to the preliminary themes. We then re-evaluated the remaining eligible studies and searched for confirming and disconfirming cases and selected additional studies for data extraction [29]. We selected those that expanded on existing themes, added illustrative examples, added data for new themes or contradicted existing themes. Two authors (IEW and AR) independently assessed these studies for inclusion, proceeded with independent coding and discussed each study to resolve discrepancies. The additional data helped expand on and refine preliminary themes. This process was continued until no new themes emerged and data saturation had been reached. |
| 18 | Derivation of themes | Explain whether the process of deriving the themes or constructs was inductive or deductive. | Yes, we initially started with a deductive approach to identify known barriers, and used inductive coding to identify new codes | In our protocol, we developed a conceptual model based on factors identified from the literature, which influence linkage, retention in care and adherence (S1 Fig) that informed our preliminary coding framework (S3 Appendix). Two authors (IEW and AR or LH) independently coded studies deductively, using the coding framework, and inductively, identifying new codes using a grounded theory approach. |
| 19 | Quotations | Provide quotations from the primary studies to illustrate themes/constructs, and identify whether the quotations were participant quotations of the author’s interpretation. | Yes, Detailed evidence annexes with codes are provided | S1 Evidence annex – S9 Evidence annex with supporting quotes |
| 20 | Synthesis output | Present rich, compelling and useful results that go beyond a summary of the primary studies (e.g. new interpretation, models of evidence, conceptual models, analytical framework, development of a new theory or construct). | Yes, we present a conceptual model and new interpretation | We further highlight this in our conclusions: This review goes beyond presenting barriers and facilitators and describes broadly how various external and internal factors interact and drive ART adherence and engagement decisions, and presents a model for understanding ‘why people do what they do’. |
