# Supplementary material for: Being HIV positive and staying on antiretroviral therapy in Africa: A qualitative systematic review and theoretical model
Source: PLoS One. 2019 Jan 10;14(1):e0210408. doi: 10.1371/journal.pone.0210408 (PMC6328200; doi:10.1371/journal.pone.0210408)
Supplement: S1 Table — (DOCX) [file pone.0210408.s005.docx]

| \| 1. Were steps taken to increase rigour in the sampling? *Consider whether: *the sampling strategy was appropriate to the questions posed in the study (e.g. was the strategy well reasoned and justified?); *attempts were made to obtain a diverse sample of the population in question (think about who might have been excluded; who may have had a different perspective to offer); *characteristics of the sample critical to the understanding of the study context and findings were presented (i.e. do we know who the participants were in terms of, for example, basic socio-demographics, characteristics relevant to the context of the study, etc.).* \| \| --- \| | Yes, a fairly thorough attempt was made  Yes, several steps were taken  Yes, a few steps were taken  No, not at all/Not stated/ Can’t tell |
| --- | --- | --- |
| \| 1. Were steps taken to increase rigour in the data collected? *Consider whether: *data collection tools were piloted/(and if quantitative) validated; *(if qualitative) data collection was comprehensive, flexible and/or sensitive enough to provide a complete and/or vivid and rich description of people’s perspectives and experiences (e.g. did the researchers spend sufficient time at the site/with participants? Did they keep ‘following up’? Was more than one method of data collection used?); * steps were taken to ensure that all participants were able and willing to contribute (e.g. processes for consent, language barriers, power relations between adults and children/young people).* \| \| --- \| | Yes, a fairly thorough attempt was made  Yes, several steps were taken  Yes, a few steps were taken  No, not at all/Not stated/ Can’t tell |
| 1. Were steps taken to increase rigour in the analysis of the data? *Consider whether: * data analysis methods were systematic (e.g. was a method described/can a method be discerned?); *diversity in perspective was explored; * (if qualitative) the analysis was balanced in the extent to which it was guided by preconceptions or by the data); *the analysis sought to rule out alternative explanations for findings (in qualitative research this could be done by, for example, searching for negative cases/exceptions, feeding back preliminary results to participants, asking a colleague to review the data, or reflexivity; in quantitative research this may be done by, for example, significance testing).* | Yes, a fairly thorough attempt was made  Yes, several steps were taken  Yes, a few steps were taken  No, not at all/Not stated/ Can’t tell |
| 1. Were the findings of the study grounded in/ supported by the data? *Consider whether: *enough data are presented to show how the authors arrived at their findings; *the data presented fit the interpretation/support claims about patterns in data; *the data presented illuminate/illustrate the findings; *(for qualitative studies) quotes are numbered or otherwise identified and the reader can see that they don’t just come from one or two people* | Yes, a fairly thorough attempt was made  Yes, several steps were taken  Yes, a few steps were taken  No, not at all/Not stated/ Can’t tell |
| 1. Please rate the findings of the study in terms of their breadth and depth. *Consider whether: (NB: it may be helpful to consider ‘breadth’ as the extent of description and ‘depth’ as the extent to which data has been transformed/analysed); *a range of issues are covered; * the perspectives of participants are fully explored in terms of breadth (contrast of two or more perspectives) and depth (insight into a single perspective); *richness and complexity has been portrayed (e.g. variation explained, meanings illuminated); *there has been theoretical/conceptual development.* | Yes, a fairly thorough attempt was made  Yes, several steps were taken  Yes, a few steps were taken  No, not at all/Not stated/ Can’t tell |

S1 Table: EPPI – Quality appraisal tool
