# Supplementary material for: Being HIV positive and staying on antiretroviral therapy in Africa: A qualitative systematic review and theoretical model
Source: PLoS One. 2019 Jan 10;14(1):e0210408. doi: 10.1371/journal.pone.0210408 (PMC6328200; doi:10.1371/journal.pone.0210408)
Supplement: S2 Table — (DOCX) [file pone.0210408.s006.docx]

| **Author / year** | **Were steps taken to increase rigour in the sampling?** | **Were steps taken to increase rigour in the data collected?** | **Were steps taken to increase rigour in the analysis of the data?** | **Were the findings of the study grounded in/ supported by the data?** | **Please rate the findings of the study in terms of their breadth and depth.** |
| --- | --- | --- | --- | --- | --- |
| Asgary 2014 | 2 | 3 | 2 | 2 | 2 |
| Assefa 2014 | 2 | 1 | 1 | 1 | 1 |
| Axellson 2015 | 3 | 2 | 2 | 3 | 2 |
| Beckmann 2013 | 3 | 3 | 3 | 3 | 3 |
| Belisle 2014 | 3 | 3 | 3 | 3 | 3 |
| Bezabhe 2014 | 2 | 3 | 3 | 3 | 3 |
| Bhagwanjee2013 | 3 | 3 | 3 | 3 | 3 |
| Bogart 2013 | 3 | 3 | 3 | 3 | 2 |
| Braga 2013 | 3 | 3 | 3 | 3 | 3 |
| Busza 2014 | 3 | 3 | 2 | 2 | 1 |
| Campbell 2015 | 3 | 3 | 3 | 3 | 3 |
| Cange 2015 | 3 | 3 | 3 | 3 | 2 |
| Coetzee 2015 | 1 | 1 | 2 | 3 | 1 |
| Dlamini-Simelani 2016 | 3 | 3 | 3 | 3 | 3 |
| Elwell2016 | 3 | 3 | 3 | 3 | 3 |
| Fielding-Miller2014 | 3 | 3 | 1 | 3 | 2 |
| Gourlay 2014 | 3 | 3 | 3 | 3 | 3 |
| Guise2017 | 3 | 3 | 3 | 2 | 2 |
| Hatcher2016 | 3 | 3 | 3 | 3 | 3 |
| Hornschuh 2014 | 3 | 3 | 2 | 3 | 2 |
| Inzaule 2016 | 3 | 3 | 3 | 3 | 3 |
| Jones 2014 | 3 | 3 | 3 | 3 | 3 |
| Katirayi2016 | 3 | 3 | 3 | 3 | 2 |
| Katz 2017 | 2 | 2 | 2 | 3 | 2 |
| Kawuma 2014 | 3 | 3 | 3 | 3 | 3 |
| Kennedy2013 | 3 | 3 | 3 | 2 | 2 |
| Kim 2016 | 3 | 2 | 2 | 3 | 3 |
| Layer 2014 | 3 | 2 | 1 | 2 | 2 |
| Layer 2014 | 3 | 2 | 3 | 3 | 2 |
| Maeri 2016 | 3 | 3 | 3 | 3 | 3 |
| Masquiller 2015 | 3 | 3 | 3 | 3 | 3 |
| Mattes 2014 | 3 | 3 | 2 | 3 | 3 |
| Mbonye 2016 | 3 | 3 | 2 | 3 | 2 |
| Mburu 2014 | 3 | 2 | 3 | 3 | 3 |
| Mburu 2014 | 3 | 3 | 3 | 3 | 3 |
| McMahon2017 | 3 | 3 | 3 | 3 | 3 |
| Mendelsohn 2014 | 3 | 3 | 3 | 3 | 3 |
| Mtetwa 2013 | 3 | 3 | 2 | 3 | 2 |
| Mutumba2015 | 3 | 3 | 3 | 3 | 2 |
| Mutwa2013 | 3 | 3 | 2 | 2 | 2 |
| Naik 2013 | 3 | 3 | 3 | 3 | 3 |
| Nakanwagi2016 | 3 | 3 | 2 | 3 | 2 |
| Ngarina2013 | 3 | 3 | 3 | 3 | 3 |
| Niehaus2013 | 3 | 3 | 3 | 3 | 3 |
| Okoror 2013 | 3 | 3 | 3 | 3 | 3 |
| Parsons 2016 | 3 | 3 | 3 | 3 | 3 |
| Rasmussen 2013 | 3 | 3 | 3 | 3 | 3 |
| Russel 2015 | 3 | 3 | 3 | 3 | 3 |
| Russel 2016 | 3 | 3 | 3 | 3 | 3 |
| Saleem2016 | 3 | 2 | 2 | 3 | 3 |
| Scott 2013 | 3 | 3 | 2 | 3 | 2 |
| Sikstrom 2014 | 3 | 3 | 1 | 3 | 3 |
| Sikweyiya2014 | 3 | 3 | 3 | 3 | 3 |
| Siu 2013 | 3 | 3 | 2 | 3 | 3 |
| Siu 2014 | 3 | 3 | 2 | 3 | 3 |
| Ware2013 | 3 | 3 | 3 | 3 | 3 |
| Watt2016 | 3 | 3 | 3 | 3 | 3 |
| Wolf 2014 | 3 | 3 | 3 | 3 | 3 |
| Wouters 2016 | 3 | 3 | 3 | 3 | 3 |
| Yoshida 2014 | 3 | 3 | 3 | 3 | 3 |
| Zissette2016 | 3 | 3 | 3 | 3 | 3 |
| 0 = Not at all, not stated, can't tell | | | | | |
| 1 = A few steps were taken | | | | | |
| 2 = Several steps were taken | | | | | |
| 3 = A fairly thorough attempt was made | | | | | |

S3 Table: Critical appraisal of included studies
