# Supplementary material for: Being HIV positive and staying on antiretroviral therapy in Africa: A qualitative systematic review and theoretical model
Source: PLoS One. 2019 Jan 10;14(1):e0210408. doi: 10.1371/journal.pone.0210408 (PMC6328200; doi:10.1371/journal.pone.0210408)
Supplement: S1 Evidence Annex — (DOCX) [file pone.0210408.s007.docx]

| **Theme 1: Poverty, competing priorities and an unpredictable microworld** | | | | | |
| --- | --- | --- | --- | --- | --- |
| **Sub-themes** | **Codes** | **Sub-code (1)** | **Sub-code (2)** | **Illustrative quote(s)** | **Supporting papers** |
| **Poverty and the need to prioritize** | **Economic constraints** | Need to earn money instead of attend clinic |  | “Opportunity costs included taking time away from income generating activities and risking loss of employment for those with formal jobs.”(1)  “Patients who had to take time off from work for clinic visits feared losing pay or being terminated as a consequence.”(2)  “It is evident that in this community, barriers such as lack of time and finances play an important role in the linkage to care. These factors may not be firm logistic constraints, but rather contextual elements that work to subtly push care seeking further down on the list of personal priorities. It is not that people do not have money at all, but rather that they are forced to make difficult choices about how to use their limited finances.” (3) | (1-11) |
|  |  | Food insecurity | Adverse events potentiated by hunger leads some to stop ART | “Women reported worsening of side effects when they took ART without food and they stopped ART occasionally when they did not have enough food (N = 4). Women may accept ART for life,but have poor adherence during times of food shortage and side effects become too severe to manage.” (12) | (5, 12-15) |
|  |  |  | Increased appetite on ART may be a challenge for household | “Another often mentioned adverse reaction of the drugs was the almost insatiable hunger they induced (cf. Kalofonos, 2010). In the face of most households' chronic economic constraints – which reflected the critical insufficiency of the local formal and informal labor market and the increasing cleavage between stagnating meager incomes and rapidly rising commodity prices in the wake of the global economic crisis – this was perceived as a severe problem.” (16)  “Respondents stated that a healthy child threatened the financial stability of many households, as an improvement in health also meant a greater appetite, thus requiring more food for the household.”(17) | (7, 13, 16, 17) |
|  |  | Cost of travel and services |  | “To get to the clinic, she took two buses, making bus fare a constraint for timely access to care. “(18)  “Women in particular were deeply concerned and anguished over the need for money to pay fees and medication for themselves and their children. “ (13) | (1, 2, 4, 8, 13, 18-22) |
|  | **Safety concerns** |  |  | “The first pathway was partner disclosure, with violent relationships framing a decision to hide one's HIV status. Some women were unable to maintain careful PMTCT behaviors without risking disclosure, so they opted to take treatment breaks or stop treatment altogether.”(23)   “The level of brutality directed towards MSM was described by one participant. This brutality may include threats of extortion, disclosure of their sexual orientation, physical assault and/or sexual abuse” (24) | (3, 23-25) |
|  | **The need to maintain a social life** | Social and family responsibilities |  | “Interviewees continually confronted competing demands on their time. When demands stemmed from cultural and family obligations, or economic requirements—e.g., caring for a sick family member in a distant location, traveling for work, attending a relative’s funeral in another town—they took precedence over keeping clinic appoint- ments” (21)  “For example, items such as food, transport to work, attending to the needs of other family members, or purchasing children's school uniforms are likely to take priority over care seeking. These "more important" items may repeatedly displace care seeking, diminishing its urgency over time, particularly if clients are still feeling well and unhindered by their illness.”(3) | (3, 5, 21, 26) |
|  |  | Social and family network may be essential for survival |  | “Women also described heightened vulnerabilities related to pregnancy, birth, and motherhood as an additional reason to disengage from care. Respondents described how this period of their lives involved a heavier reliance on family and, to a lesser extent, the broader community, which could be irreparably jeopardized by an HIV-positive status and its accompanying social isolation. In a particularly disturbing case, a woman whose status became known described how she was hemorrhaging during delivery, but nobody in the community or her extended family would donate blood or money to help her survive.” (9)  “…even under the most adverse conditions, people maintain and are guided by a tenacious connection to socially meaningful life, embedded in webs of social relations. This social embeddedness becomes ever more important in the Tanzanian setting, which is characterised by a form of crisis as context…” (27) | (9, 18, 21, 25, 27-29) |
| **Unpredictable life events** | **High mobility and unexpected travel disruptions** |  |  | “Plans were routinely upended by unexpected events, another source of unintentional absences. Accidents, vehicle breakdowns, and other travel delays meant that individuals who had traveled but planned to return before their clinic visit date were stranded away from home.”(21)  “Some women explained that they had to travel frequently and cited this as the reason for poor drug adherence. So most of the women were very mobile and this tended to interfere with proper drug adherence since they sometimes run out of the drugs, forget to take them on a trip, or got motion sickness while travelling, which prevented them from taking the pills.”(5) | (3, 5, 10, 21, 23, 30) |
|  | **Unexpected change in routines** |  |  | “The reason most frequently listed by the patients as a barrier to adherence was a change in routines or an unpredicted event, which could cause the patient to miss doses for various time periods, ranging from days to weeks…..Similarly, one woman said she attended a funeral, another had to take a sick child to the hospital, and a man was prevented from leaving his job because of a flood that made medicine unavailable at dose time.”(7) | (7) |
|  | **Sudden loss of support system** |  |  | “The death of a primary caregiver was particularly dangerous since it led to treatment disruptions and modifications. Katherine, a five-year old girl with HIV was improving slowly after initiating ART treatment; after years of debilitating illness she was finally able to play outside with friends and go to school. However, soon after her mother's death from HIV-related complications Katherine was sent to live with her father's family in another village. In less than a month, Katherine's health deteriorated so rapidly that another aunt became so alarmed that she carried Katherine that day to hospital on her back where she was admitted and treated for advanced symptoms of HIV infection.” (31)  In a similar account provided by a disabled client, the combination of the physical disability and the departure of a treatment supporter lead to a treatment interruption, highlighting the importance of durable treatment support networks for highly vulnerable clients. (15) | (15, 31) |
|  | **Political unrest and refugee status** |  |  | “Jeffrey, a Rwandese refugee who fled to Kenya, described a treatment interruption that resulted from having fled from his home in Rwanda. The urgency of the departure prevented him from organizing a sufficient supply of medication to cover the unknown duration of the transit period spanning his departure and eventual arrival in Kakuma Refugee Camp.”(15) | (15) |

1. Busza J, Dauya E, Bandason T, Mujuru H, Ferrand RA. "I don't want financial support but verbal support." How do caregivers manage children's access to and retention in HIV care in urban Zimbabwe? J Int AIDS Soc. 2014;17:18839.

2. Bogart LM, Chetty S, Giddy J, Sypek A, Sticklor L, Walensky RP, et al. Barriers to care among people living with HIV in South Africa: contrasts between patient and healthcare provider perspectives. AIDS Care. 2013;25(7):843-53.

3. Naik R. Linkage to care following

home-based HIV counseling and testing: a mixed methods study in rural South Africa: University of Boston; 2013.

4. Nakanwagi S, Matovu JK, Kintu BN, Kaharuza F, Wanyenze RK. Facilitators and Barriers to Linkage to HIV Care among Female Sex Workers Receiving HIV Testing Services at a Community-Based Organization in Periurban Uganda: A Qualitative Study. J Sex Transm Dis. 2016;2016:7673014.

5. Ngarina MP, R.; Kilewo, C.; Beberfeld, G.; Ekstrom, A., M. Reasons for poor adherence to antiretroviral therapy postnatally in HIV-1 infected women treated for their own health: experiences from the Mitra Plus study in Tanzania. BMC Public Health. 2013;13(450):<http://www.biomedcentral.com/1471-2458/13/450>.

6. Katz IT, Bogart LM, Cloete C, Crankshaw TL, Giddy J, Govender T, et al. Understanding HIV-infected patients' experiences with PEPFAR-associated transitions at a Centre of Excellence in KwaZulu Natal, South Africa: a qualitative study. AIDS Care. 2015;27(10):1298-303.

7. Axelsson JM, Hallager S, Barfod TS. Antiretroviral therapy adherence strategies used by patients of a large HIV clinic in Lesotho. J Health Popul Nutr. 2015;33:10.

8. Gourlay AW, A.; Birdthisle, I.; Mshana, G.; Michael, D.; Urassa, M. ‘‘It Is Like That, We Didn’t Understand Each Other’’: Exploring the Influence of Patient-Provider Interactions on Prevention of Mother-To-Child Transmission of HIV Service Use in Rural Tanzania. PLoS One. 2014;9(9).

9. McMahon SA, Kennedy CE, Winch PJ, Kombe M, Killewo J, Kilewo C. Stigma, Facility Constraints, and Personal Disbelief: Why Women Disengage from HIV Care During and After Pregnancy in Morogoro Region, Tanzania. AIDS and Behavior. 2016;21(1):317-29.

10. Thorne C, Bezabhe WM, Chalmers L, Bereznicki LR, Peterson GM, Bimirew MA, et al. Barriers and Facilitators of Adherence to Antiretroviral Drug Therapy and Retention in Care among Adult HIV-Positive Patients: A Qualitative Study from Ethiopia. PLoS ONE. 2014;9(5).

11. Wouters E, De Wet K. Women's experience of HIV as a chronic illness in South Africa: hard-earned lives, biographical disruption and moral career. Sociol Health Illn. 2016;38(4):521-42.

12. Kim MH, Zhou A, Mazenga A, Ahmed S, Markham C, Zomba G, et al. Why Did I Stop? Barriers and Facilitators to Uptake and Adherence to ART in Option B+ HIV Care in Lilongwe, Malawi. PLoS One. 2016;11(2):e0149527.

13. Braga B, M., T. “Death is Destiny”: Sovereign Decisions and the Lived Experience of HIV/AIDS and Biomedical Treatment in Central Mozambique: University at Buffalo, State University of New York; 2013.

14. Russell S, Martin F, Zalwango F, Namukwaya S, Nalugya R, Muhumuza R, et al. Finding Meaning: HIV Self-Management and Wellbeing among People Taking Antiretroviral Therapy in Uganda. PLoS One. 2016;11(1):e0147896.

15. Mendelsohn JB, Rhodes T, Spiegel P, Schilperoord M, Burton JW, Balasundaram S, et al. Bounded agency in humanitarian settings: a qualitative study of adherence to antiretroviral therapy among refugees situated in Kenya and Malaysia. Soc Sci Med. 2014;120:387-95.

16. Mattes D. “Life is not a rehearsal, it's a performance”: An ethnographic enquiry into the subjectivities of children and adolescents living with antiretroviral treatment in northeastern Tanzania. Children and Youth Services Review. 2014;45:28-37.

17. Coetzee B, Kagee A, Bland R. Barriers and facilitators to paediatric adherence to antiretroviral therapy in rural South Africa: a multi-stakeholder perspective. AIDS Care. 2015;27(3):315-21.

18. Dlamini-Simelane TTT, Moyer E. ‘Lost to follow up’: rethinking delayed and interrupted HIV treatment among married Swazi women. Health Policy and Planning. 2016.

19. Campbell C, Scott K, Skovdal M, Madanhire C, Nyamukapa C, Gregson S. A good patient? How notions of 'a good patient' affect patient-nurse relationships and ART adherence in Zimbabwe. BMC Infect Dis. 2015;15:404.

20. Layer EH, Brahmbhatt H, Beckham SW, Ntogwisangu J, Mwampashi A, Davis WW, et al. "I pray that they accept me without scolding:" experiences with disengagement and re-engagement in HIV care and treatment services in Tanzania. AIDS Patient Care STDS. 2014;28(9):483-8.

21. Ware NC, Wyatt MA, Geng EH, Kaaya SF, Agbaji OO, Muyindike WR, et al. Toward an understanding of disengagement from HIV treatment and care in sub-Saharan Africa: a qualitative study. PLoS Med. 2013;10(1):e1001369; discussion e.

22. Guise A, Rhodes T, Ndimbii J, Ayon S, Nnaji O. Access to HIV treatment and care for people who inject drugs in Kenya: a short report. AIDS Care. 2016;28(12):1595-9.

23. Hatcher AM, Stockl H, Christofides N, Woollett N, Pallitto CC, Garcia-Moreno C, et al. Mechanisms linking intimate partner violence and prevention of mother-to-child transmission of HIV: A qualitative study in South Africa. Soc Sci Med. 2016;168:130-9.

24. Cange CW, LeBreton M, Billong S, Saylors K, Tamoufe U, Papworth E, et al. Influence of stigma and homophobia on mental health and on the uptake of HIV/sexually transmissible infection services for Cameroonian men who have sex with men. Sex Health. 2015;12(4):315-21.

25. Maeri I, El Ayadi A, Getahun M, Charlebois E, Akatukwasa C, Tumwebaze D, et al. "How can I tell?" Consequences of HIV status disclosure among couples in eastern African communities in the context of an ongoing HIV "test-and-treat" trial. AIDS Care. 2016;28 Suppl 3:59-66.

26. Jones C. Between State and Sickness: The Social Experience of HIV/AIDS illness management and treatment in Grahamstown, South Africa [Dissertation]: Graduate School-New Brunswick

Rutgers, The State University of New Jersey; 2014.

27. Beckmann N. Responding to medical crises: AIDS treatment, responsibilisation and the logic of choice. Anthropol Med. 2013;20(2):160-74.

28. Elwell K. Social and Structural Factors Affecting Women’s Participation in prevention of mother to child transmission(PMTCT) programs in Malawi. Antrhopology. 2015;Doctor of Philosophy:210.

29. Masquillier C, Wouters E, Mortelmans D, van Wyk B. On the road to HIV/AIDS competence in the household: building a health-enabling environment for people living with HIV/AIDS. Int J Environ Res Public Health. 2015;12(3):3264-92.

30. Inzaule SC, Hamers RL, Kityo C, Rinke de Wit TF, Roura M. Long-Term Antiretroviral Treatment Adherence in HIV-Infected Adolescents and Adults in Uganda: A Qualitative Study. PLoS One. 2016;11(11):e0167492.

31. Sikstrom L. “Without the grandparents, life is difficult”: Social hierarchy and therapeutic trajectories for children living with HIV in rural Northern Malawi. Children and Youth Services Review. 2014;45:47-54.
