# Supplementary material for: Being HIV positive and staying on antiretroviral therapy in Africa: A qualitative systematic review and theoretical model
Source: PLoS One. 2019 Jan 10;14(1):e0210408. doi: 10.1371/journal.pone.0210408 (PMC6328200; doi:10.1371/journal.pone.0210408)
Supplement: S2 Evidence Annex — (DOCX) [file pone.0210408.s008.docx]

| **Theme 2:** Social identity can have a profound impact on care-seeking behavior | | | | | |
| --- | --- | --- | --- | --- | --- |
| Sub-themes | Codes | Sub-code (1) | Sub-code (2) | Illustrative quote(s) | Supporting papers |
| **Gender roles interact with HIV care** | Masculinty is at odds with HIV care | Hegemonic features of masculinity | Strong, powerful and brave, risk-takers | “But our data suggest that men under about 40 may have found it particularly difficult to admit to physical weakness and accept illness, perhaps because they were more oriented to expressing their masculinity through physical strength…”(1)   “Social expectations for men to be risk-takers emerged from interviews and focus group discussion with people living with HIV and health providers.” (2) | (1-3) |
|  |  |  | Respected in society | “Maintaining a presence in the community and being known by others in the community is, for the participants, integral to the idea of being respected within the community as a man.” (4) | (2, 4-6) |
|  |  |  | Provide economically for their families | “Work was important for material wellbeing and feeling a sense of independence, particularly for men’s fulfilment of their expected masculine role of provider..”(6) | (1, 2, 4-7) |
|  |  |  | Father children and have a family | “It is interesting that, as noted previously, the notion of family was the one men connected most to being a “real man.” ” (4) | (4, 5) |
|  |  | Interaction between masculinity and HIV | HIV may erode masculine identity | “What is depicted in the above narratives as an occupied position by these men is diminished manhood, a loss position that was fairly consistently described as following the diagnosis.” (5)  “One mechanism by which HIV stigma was perceived to interact with masculinity to prevent men from accessing services was linked to a sense of shame, secrecy, powerlessness and a loss of respect, qualities that were all contrary to masculine notions of respect.” (2)  In general, participants’ accounts suggested that men living with HIV infection and/or ART, both younger and older, endured levels of mistreatment and oppression in their households in ways that substantially contradicted their expectation to enjoy the traditional masculine privilege of respect.(3)  “Waiting times in clinics were also a hindrance. First, waiting with women contradicted the local gender norm that men are served before women” (1) | (1-3, 5) |
|  |  |  | Attending HIV clinics is at odds with masculine identity | “Endorsing male independence, resilience and being dismissive of problems made men reluctant to acknowledge the seriousness of HIV symptoms and assume a sick identity until the symptoms were acute and life threatening. For some men, not starting or delaying to start ART was an achievement and an important part of their masculine identity, which attested to their physical and emotional strength despite symptoms.” (1)  “Participants’ accounts suggested that men often struggled with the idea that they should seek and utilise health services, and accept being linked to long-term care, because as men, they were expected to be physically and mentally strong..” (2)  The use of traditional medicine was linked directly to what it means to “be a man” by participants; the sentiments that “men do not go to clinics,” yet “the first thing a man does when he gets ill is run to [traditional healers] to be healed,” were reiterated by most participants. (4)  “Waiting times in clinics were also a hindrance. First, waiting with women contradicted the local gender norm that men are served before women”(1) | (1, 2, 4, 5) |
|  | Women often have limited choices | Women have defined social roles | Women want to have children and society expects it | “Having children generally has a high social value in Uganda, especially for women, for whom giving birth makes them culturally and socially ‘complete’”(8)  “Just as “friends” may mock women for being diagnosed as HIV-positive, they also mock those women unable to bear children. Informants report that those most likely to deride women for not having children are their female peers.”(9) | (8-10) |
|  |  |  | Women are expected to care for others and may not put themselves or their health first | “Within this context, women in this study have largely positioned themselves with the ‘‘duty’’ of being a carer for the entire family, thereby reducing their own agency in terms of the (re)assignment of this role. “(11)  “Given their extensive responsibilities – caring for their own and other’s children and elderly family members in difficult circumstances while struggling to generate an income – normal roles cannot be cast away to take on the sick role”…. “The women find themselves in a dual position: requiring care themselves as PLWHA on ART and secondly, providing care to others in need. Throughout our interviews, it becomes apparent that the latter role gains ascendancy.” (10) | (9-14) |
|  |  |  | Need permission from others before making health decisions | “They placed great value on their marital relations and stated that making healthcare decisions prior to consulting spouses would jeopardise those relations” ….” Both stories demonstrate that when a married woman has been diagnosed with HIV, she is not free to initiate treatment, even when it is prescribed. Neither is she free to decide on her own to return to the clinic, especially if doing so requires money.” (15). | (9, 15-17) |
|  |  | Economic dependency results in limited choices | Vulnerability due to dependence on husbands/partners | “Data from interviews with both providers and community members revealed concerns centering on women’s vulnerability due to financial dependence on partners” (18)  “Several informants noted the gendered consequences of divorce, highlighting economic consequences as central to women’s fear of HIV status disclosure. Women may hesitate to disclose an AIDS status because they fear being “chased” or abandoned. Divorce for many women has both social and economic consequences, demonstrating the importance of women’s identity as a wife. The economic effects are most salient, as many PMTCT patients rely upon their husbands for economic security.”(9) | (9, 15, 18-21) |
|  |  |  | Poor women are more dependent on family and social network for economic, logisitic and emotional help | “Most women with small children are dependent on others for support and childcare, so having to take drugs secretly at definite times and having to exclusively breastfeed sometimes posed an insurmountable challenge.” (20)  Higher-status professional community women with greater economic and decision- making power were able to more directly challenge potential power inequities within their relationships by deploying power discursively. These women divorced husbands when the relationship was not meeting their needs, employed discursive strategies to get their social and economic needs met in the relationship, and exhibited greater freedom with discursive strategies… (9) | (9, 20) |
|  |  | Unsupportive partners, IPV and sexual abuse impact health decisions and access to care | Unsupportive or obstructive partners | “Some women faced obstructive behavior from their husbands, such as throwing away the pills”. (16)  “A third pathway was partner control and isolation, in which men limited participant access to friends and family, which precluded the social support required for good adherence.”(13) | (11, 13, 16, 18, 22) |
|  |  |  | Fear of IPV | “Participants described this choice as a reasoned response to a dangerous situation. Six participants feared that their partners would react to disclosure with physical violence. For Simphiwe, a 33-year-old woman who had been with her partner for five years, a history of physical violence led her to keep HIV a secret.” (13) | 3, 12, 23, 25, 39) |
|  |  |  | IPV and sexual abuse leads to depression and hopelessness | “A second pathway was mental health, as IPV resulted in depressive views that “life is not worth living” and led to missing doses of medication.”(13)  Approximately a quarter of women in this study described feelings of despair. One woman, who contracted HIV and became pregnant as a result of rape by her employer, used the phrase ‘‘I am empty’’ several times throughout her interview.(19) | (13, 19, 23) |
|  |  | Maintaining gender roles and complying with HIV guidelines is a challenge | Women struggle to reduce family size | “A related issue is the desire to have children: HIV has changed these women’s lives as they – despite the availability of drugs for prevention-of-mother-to-child-transmission (PMTCT) – feel forced to forget the idea of having children because of their status. The practical consequences of HIV thus have important symbolic significance on the lives and resulting identity of these women…” (10)  “Grounding their statements in biomedical risk discourse, these providers assume that HIV-positive women should not have more children because of the nature of their positive status…. Reports of negative attitudes toward women who are HIV-positive and continue to have children also emerged in discussions with current PMTCT patients during a focus group discussion when they were asked about whether they had ever experienced stigma.”(9) | (8-10, 24) |
|  |  |  | Women may choose rather to maintain family/social life if taking ART could threaten this | “There is no doubt that these delays, caused by her desire to be a good wife and her economic dependence on her husband, reduced the likelihood that treatment would be effective and very likely contributed to her death”(15)   “Nearly every woman in this study described how the potential to be socially or economically isolated—in other words, stigmatized—undermined her motivation to continue PMTCT” (19)   “These clients appear to feel "stuck" in a sense, as they may be willing to address the diagnosis and seek care, but lack the support to do so, and feel afraid to jeopardize their relationships.”(22) | (15, 19, 22) |
| **It is confusing and isolating being a child or adolescent with HIV:** | Influence of developmental stage | Strong desire to be like other “normal” children/adolescents |  | “The adolescents used the words ‘‘normal’’ and ‘‘healthy’’ interchangeably throughout the FGDs. Adolescents emphasized the wish to continue living as their HIV uninfected peers.”(25)  “Being reminded, and the way the reminders were delivered, disrupted these children’s desire to be seen as normal children, able to do all the things that other children do, like play.”(26) | (25-27) |
|  |  | Anger, rebellion and confusion |  | This anger in some cases also led to the adolescent being less adherent, sometimes in an attempt to punish their parents and other times in coming to terms or due to confusion about why this happened to them and not to others (sometimes their own siblings) (25)  In one case a child, Duncan (13 years old) felt so poorly treated by his aunt that he decided to stop taking his drugs and not to disclose this because he hoped that he would die. (26) | (25, 26) |
|  |  | Adolescents want to have a future |  | “When asked about their expectations and worries concerning their future, the children's main concern was not related to their health status or the long-term consequences of being on treatment. Rather, they desperately wished to obtain the best possible education that would allow them to find good work and enable them to care for themselves and their extended families.”(28) | (28) |
|  |  | Adolescents struggle to negotiate sexual relationships with HIV |  | “Fear of transmitting HIV to their sexual partners was also expressed in their narratives.”(27)  “An additional barrier relating to adolescent disclosure was fear of abandonment by romantic or sexual partners. Adolescent girls interviewed who had not disclosed their HIV status to their boyfriends cited the fear that disclosure could end their relationships”(29) | (27, 29) |
|  | Highly dependent on support from others | Need stable home and school environments to thrive |  | “The home and school environments are the most salient socio-ecological contexts for adolescent wellbeing and development. Arguably, the presence of adversity within these contexts undermines the well-being of all ALH.”(27) | (14, 25, 27, 30) |
|  |  | There is often a lot of chaos and instability in their support systems |  | “Further, although grandmothers were the primary caregiver, other household members also administered medication to the child, which became problematic when doses were changed.” (31)  “The extensive mobility of children between TMGs (treatment management groups) for reasons ranging from the divorce or death of a family member to providing companionship and labor to an elderly relative also means that over the course of a child's treatment trajectory, there were numerous shifts within the child's TMG.” (14) | (14, 31) |
|  | Children and adolescent are at risk of feeling isolated and depressed | HIV positive children/adolescents experience a lot of losses |  | “In Africa and particularly Rwanda, a large number of HIV-infected children are living without parents, as their parents either died from HIV-related conditions, recent conflict, or other healthcare causes.”(25)  “A striking detail concerning the situation of all children participating in the study was their heavy psychological burden due to traumatic personal experiences. Almost all had lost at least one parent. Several had been passed from one relative to another after losing both parents. Some children's fathers were unknown, others had rejected them after their mothers' death for economic reasons or because they would not face the implications regarding their own possible HIV infection.”(28) | (14, 25, 28) |
|  |  | Depression due to HIV diagnosis |  | “ A few respondents resorted to social isolation in a bid to stem off questions about their illness.” ….. “The lack of disclosure reinforced adolescents’ sense of isolation from both siblings and peers, heightened their emotional distress, and undermined their ability to adhere to their medications and seek social support, particularly when the lack of disclosure occurred within the family” (27)  “The adolescents discussed that non-acceptance of their HIV status, could lead to feelings of depression and isolation. (25)  “Other children attributed temporary deteriorations of their health (as for instance a decrease of their CD4 level) and school performance to negative emotional states like sadness and “too much thinking” (mawazo) and consequently tried to suppress them completely.(28) | (25, 27, 28) |
|  | Adults often do not know how to deal with children and adolescents | Disclosure | Caretakers find it hard to disclose HIV status | “Among parents who were themselves living with HIV, stigma imposed a double burden: parents who had not come to terms with their own HIV status were likely to find it even more difficult to disclose to their children about the children’s HIV infection.”(29) | (29, 30) |
|  |  |  | Social norms may influence communications | “Local norms relating to sexuality also appeared to discourage parents from telling their adolescent children that they had HIV. (29) |  |
|  |  | Health workers may not communicate directly |  | If children were at all present in the adherence classes, there was no room to address their concerns in an adequate way. During follow-up visits children were rarely asked about their difficulties with regard to their drug intake and the assessment of their recovery process was mostly done via their caretakers.(28) | (28, 29) |
|  |  | Transfer to general or adult clinics is difficult |  | “Eight participants from the pediatric clinic stated that they would under no circumstances agree to be transitioned to a different clinic, even the neighboring adult clinic. They cited relationships with the staff and familiarity with the environment as reasons for this. “(32)  “Moreover, while it was noted that adolescents were a group in need of more counseling, this was hindered by a shortage of counselors. Frequent transfers of health-care providers due to shortage in staff also posed a problem to the adolescents who found it a challenge to confide and discuss freely about their health with new health-care providers”(30) | (30, 32) |
|  |  | HCWs may hold adolescents responsible for infringements |  | “Although clinicians expected adolescents to come to clinic with their guardians and expected guardians to support youth compliance, clinicians simultaneously held youth responsible for not showing up to an appointment to the same degree they would an adult, therefore not taking into account their developmental stage and dependent role.”(33) | (31, 33) |
|  | Side effects and poor palatability of formulations |  |  | “Doctors, nurses, counsellors and traditional healers were unanimous about the difficulty that children had with the palatability of Lopinavir/ritonavir.”(31)  Most children, however, also mentioned negative aspects of their medication. They experienced manifold side effects such as skin eczema, nausea, dizziness, stomach irritations, extraordinary fatigue, lipodystrophy, and numbness and pain in their extremities. (28) | (28, 31) |
|  | Fear of rejection, lack of support and poor communication all contribute to poor adherence and engagement in care | Can't maintain art without very good support systems and stable home life |  | “This study showed that social support and living situation, including living in an orphanage or foster family, was directly linked to adherence.”(25)  “Adolescents living with biological parents were perceived to have better adherence compared to those living with caretakers. The latter were reported to lack support for food, transport, medication reminders, and accompaniment by caretakers during clinic visits. Some adolescents also reported that the caretakers denied them education support on the misconception that they would die soon.”(30) | (14, 25, 26, 29, 30) |
|  |  | Stigma, the desire to maintain status a secret and lack of privacy impairs adherence and engagement in care |  | “A few children who found it hard to find a private place would sometimes miss their dose because they were afraid of people interrupting them and finding out they were taking medication” (26)  “Stigma also manifested as fear of unintentional disclosure of HIV status. This challenge was most frequently reported by adolescents attending boarding schools, where the lack of privacy in school dormitories and perceptions of lack of confidentiality among school staff heightened the risk of such unwanted disclosure. Adolescents went to great lengths to avoid acts that could arouse suspicions about their status, for example, by avoiding the use of noisy pill bottles, by not taking medications in the presence of their peers, or frequenting the school clinic, and by not associating with known HIV-positive peers, etc.” (27) | (25-27, 30, 33) |
|  |  | Combination of poor communication and developmental stage can result in confusion about HIV and impair ART adherence |  | “Other children were convinced that if their CD4 level rose sufficiently and they continuously paid attention to proper nutrition, the doctors would eventually allow them to discontinue taking ARVs.”…..  “Inconsistent practices and unclear allocations of the responsibility for disclosure, however, left some children with fragmentary knowledge and led to situations where they were confronted with contradicting information or indirectly learned about their health status “en passant”.”(28)  The concealment of status by implicating other diseases could however impact on the adherence of the adolescents as it foils the understanding of the importance of adherence and subsequent consequences, which are specific to HIV treatment. Moreover this could result in anger and depression when the adolescents become aware of their condition.(30) | (26, 28, 30) |
| **HIV-positive key populations must face stigma related both to HIV and lifestyle choice** | Lack of conforming to social norms and leads to moral judgement from others |  |  | “This quote highlights the various spheres of everyday life where MSM are not fully accepted by their peers: religious organisations, family, tribes and the sometimes harmful consequences to which they are subjugated.”(34)  “FSWs enrolled at the government facilities mentioned discrimination from the health workers who openly exhibited a negative attitude towards sex workers. “(35) | (12, 34-37) |
|  | Discrimination related to lifestyle is often worse than HIV stigma |  |  | “In general, disclosure of HIV status tended to meet with a better reception than disclosure of sex work, but both met with mixed results at best and many of our informants reported fear of disclosure.”(12)  “For example, participants said they might disclose their HIV status to family members as they anticipated receiving some material or emotional support as a result, but they might not disclose their sexual identity to those same family members due to fear of rejection or a negative reaction.” (36) | (12, 34, 36) |
|  | Psychological distress related to MSM lifestyle can be additional burden |  |  | However, over time, many participants said they came to accept their HIV status and learn to cope with the disease. MSM also reported that they had difficulty accepting their sexuality. Some described shame related to having sexual feelings for other men. (36) | (34, 36) |
|  | Lifestyle often associated with criminality which encourages discrimination |  |  | “For example, several highly public cases have brought to the fore that MSM are being held in the Yaoundé Central Prison for violations of article 347bis. As a consequence, the resulting negative publicity from these cases has led to a higher level of scrutiny, stigma and discrimination against Cameroonian MSM. In addition to the reported human rights violations – physical assault, entrapment and attempted rape – the mere threat of this type of structural violence may cause intense stress for these MSM.” (34) | (12, 34, 37) |
|  | Loss of support |  |  | “Due to the double stigma of illicit drug use and HIV—and, for female clients especially, the triple stigma of illicit drug use, sex work and HIV—methadone clients face limited social support.” (37)  “Social networks which our informants [FSW] may have been able to draw on for material support in the past – friends and family from “back home” – were less likely to provide support because of our informants’ line of work and, at times, HIV status” (12) | (34, 36-38) |
|  | For PWID sometimes addiction trumps any other needs |  |  | “For some, their experience of addiction meant that they could not prioritise care even with this support.” (39) | (39) |
|  | Key populations avoid health facilities and need a lot of help to engage in care | Discrimination keeps key populations out of HIV care |  | “Whether women had persevered in pursuing HIV treatment, dropped out early, or never attended their first appointment, reports of active discrimination from hospital staff dominated their narratives. Women with direct experience of the clinic de- scribed how hospital nurses openly expressed their hostility to sex workers, and conducted examinations and counseling with a negative attitude” (38)  “The stigma associated with being an MSM was the pre- dominant barrier to accessing healthcare services for MSM living with HIV. Both perceived and experienced stigma in healthcare settings led to a lack of care-seeking behaviour.” (36)  “A social worker at the methadone clinic described how some methadone clients feel uncomfortable at the HIV clinic because they perceive that others will regard them as criminals due to their history of drug addiction.” (37) |  |
|  |  | Key populations need a lot of support to engage in HIV care |  | “Peer educators and fellow sex workers enabled them to start treatment by encouraging them and helping to allay their fears regarding HIV treatment and occasionally escorting them to the clinics to start medication. Some respondents said they would forget the clinic days because the clinic did not run every day but the peer educators reminded them. During the key informant interviews the peer educators mentioned how tirelessly they worked to follow up the HIV positive FSWs” (35) | (35, 39) |
| **HIV positive people with disabilities defy social expectations** | Discrimination due to disability |  |  | “Many participants [PWD] spoke about how they were seen as ‘other’ and ‘less than human’ by HCPs, both during testing for HIV and when seeking treatment. Becoming HIV-positive was seen as evidence that the PWD had engaged in sexual activity, which ran counter to prevailing assumptions that PWD are asexual.” (40)  Of the participants who had acquired disability early in life, many viewed HIV as creating an additional layer of stigma(41) | (40, 41) |
|  | Difficulty accessing care and need consistent support |  |  | “Participants who were deaf were able to make their way independently. If they experienced mobility impairments, the need for assistance was related to the severity of the impairment.” (40)  In addition, participants’ accounts demonstrated that lack of access to health services was often interlinked with their changing accessibility needs over time. One participant discussed his need for a sign language interpreter…Another participant described how the recent onset of his mobility impairment has compromised his ability to access HIV care.(41)  In a similar account provided by a disabled client, the combi- nation of the physical disability and the departure of a treatment supporter lead to a treatment interruption, highlighting the importance of durable treatment support networks for highly vulnerable clients.” (42) | (40-42) |
|  | Accepting HIV and a recent disability is difficult |  |  | “For participants who acquired their disability shortly before receiving their HIV diagnosis, details of their recently- acquired disability were central to their narratives. Coping appeared to be particularly challenging as many had little time to adapt to their new impairments.”(41) | (41) |
|  | PWD may choose to avoid attending general clinics due to discrimination |  |  | “As occurred in their accounts of interacting with HCPs, such stigmatizing experiences in the queue can have important health consequences. Again, people told of considering foregoing ART rather than being subjected to the taunts of others…The image of PWD preferring “to die quietly at home” rather than be subjected to the negative attitudes of HCPs is powerful. Here stigma is enacted in a space of care and with devastating consequences. “  ”(40) | (40) |

1. Siu GE, Seeley J, Wight D. Dividuality, masculine respectability and reputation: how masculinity affects men's uptake of HIV treatment in rural eastern Uganda. Soc Sci Med. 2013;89:45-52.

2. Mburu GR, M.; Siu, G.; Bitira, D.; Skovdal, M.; Holland, P. Intersectionality of HIV stigma and masculinity in eastern Uganda: implications for involving men in HIV programmes. BMC Public Health. 2014;14(1061):<http://www.biomedcentral.com/1471-2458/14/1061>.

3. Siu GE, Wight D, Seeley J. 'Dented' and 'resuscitated' masculinities: the impact of HIV diagnosis and/or enrolment on antiretroviral treatment on masculine identities in rural eastern Uganda. SAHARA J. 2014;11:211-21.

4. Zissette S, Watt MH, Prose NS, Mntambo N, Moshabela M. "If you don't take a stand for your life, who will help you?": Men's engagement in HIV care in KwaZulu-Natal, South Africa. Psychol Men Masc. 2016;17(3):265-73.

5. Sikweyiya YM, Jewkes R, Dunkle K. Impact of HIV on and the constructions of masculinities among HIV-positive men in South Africa: implications for secondary prevention programs. Glob Health Action. 2014;7:24631.

6. Russell S, Martin F, Zalwango F, Namukwaya S, Nalugya R, Muhumuza R, et al. Finding Meaning: HIV Self-Management and Wellbeing among People Taking Antiretroviral Therapy in Uganda. PLoS One. 2016;11(1):e0147896.

7. Scott K, Campbell C, Madanhire C, Skovdal M, Nyamukapa C, Gregson S. In what ways do communities support optimal antiretroviral treatment in Zimbabwe? Health Promot Int. 2014;29(4):645-54.

8. Rasmussen LM. Counselling clients to follow 'the rules' of safe sex and ARV treatment. Cult Health Sex. 2013;15 Suppl 4:S537-52.

9. Elwell K. Social and Structural Factors Affecting Women’s Participation in prevention of mother to child transmission(PMTCT) programs in Malawi. Antrhopology. 2015;Doctor of Philosophy:210.

10. Wouters E, De Wet K. Women's experience of HIV as a chronic illness in South Africa: hard-earned lives, biographical disruption and moral career. Sociol Health Illn. 2016;38(4):521-42.

11. Bhagwanjee A, Govender K, Reardon C, Johnstone L, George G, Gordon S. Gendered constructions of the impact of HIV and AIDS in the context of the HIV-positive seroconcordant heterosexual relationship. Journal of the International AIDS Society. 2013;16(1).

12. Fielding-Miller RM, Z.; Adams, D.; Baral, S.; Kennedy, C. “There is hunger in my community”: a qualitative study of food security as a cyclical force in sex work in Swaziland. BMC Public Health. 2014;14(79):<http://www.biomedcentral.com/1471-2458/14/79>.

13. Hatcher AM, Stockl H, Christofides N, Woollett N, Pallitto CC, Garcia-Moreno C, et al. Mechanisms linking intimate partner violence and prevention of mother-to-child transmission of HIV: A qualitative study in South Africa. Soc Sci Med. 2016;168:130-9.

14. Sikstrom L. “Without the grandparents, life is difficult”: Social hierarchy and therapeutic trajectories for children living with HIV in rural Northern Malawi. Children and Youth Services Review. 2014;45:47-54.

15. Dlamini-Simelane TTT, Moyer E. ‘Lost to follow up’: rethinking delayed and interrupted HIV treatment among married Swazi women. Health Policy and Planning. 2016.

16. Kim MH, Zhou A, Mazenga A, Ahmed S, Markham C, Zomba G, et al. Why Did I Stop? Barriers and Facilitators to Uptake and Adherence to ART in Option B+ HIV Care in Lilongwe, Malawi. PLoS One. 2016;11(2):e0149527.

17. Katirayi L, Namadingo H, Phiri M, Bobrow EA, Ahimbisibwe A, Berhan AY, et al. HIV-positive pregnant and postpartum women's perspectives about Option B+ in Malawi: a qualitative study. Journal of the International AIDS Society. 2016;19(1).

18. Maeri I, El Ayadi A, Getahun M, Charlebois E, Akatukwasa C, Tumwebaze D, et al. "How can I tell?" Consequences of HIV status disclosure among couples in eastern African communities in the context of an ongoing HIV "test-and-treat" trial. AIDS Care. 2016;28 Suppl 3:59-66.

19. McMahon SA, Kennedy CE, Winch PJ, Kombe M, Killewo J, Kilewo C. Stigma, Facility Constraints, and Personal Disbelief: Why Women Disengage from HIV Care During and After Pregnancy in Morogoro Region, Tanzania. AIDS and Behavior. 2016;21(1):317-29.

20. Ngarina MP, R.; Kilewo, C.; Beberfeld, G.; Ekstrom, A., M. Reasons for poor adherence to antiretroviral therapy postnatally in HIV-1 infected women treated for their own health: experiences from the Mitra Plus study in Tanzania. BMC Public Health. 2013;13(450):<http://www.biomedcentral.com/1471-2458/13/450>.

21. Masquillier C, Wouters E, Mortelmans D, van Wyk B. On the road to HIV/AIDS competence in the household: building a health-enabling environment for people living with HIV/AIDS. Int J Environ Res Public Health. 2015;12(3):3264-92.

22. Naik R. Linkage to care following

home-based HIV counseling and testing: a mixed methods study in rural South Africa: University of Boston; 2013.

23. Watt MH, Dennis AC, Choi KW, Ciya N, Joska JA, Robertson C, et al. Impact of Sexual Trauma on HIV Care Engagement: Perspectives of Female Patients with Trauma Histories in Cape Town, South Africa. AIDS and Behavior. 2016;21(11):3209-18.

24. Beckmann N. Responding to medical crises: AIDS treatment, responsibilisation and the logic of choice. Anthropol Med. 2013;20(2):160-74.

25. Mutwa PR, Van Nuil JI, Asiimwe-Kateera B, Kestelyn E, Vyankandondera J, Pool R, et al. Living situation affects adherence to combination antiretroviral therapy in HIV-infected adolescents in Rwanda: a qualitative study. PLoS One. 2013;8(4):e60073.

26. Kawuma R, Bernays S, Siu G, Rhodes T, Seeley J. ‘Children will always be children’: Exploring perceptions and experiences of HIV-positive children who may not take their treatment and why they may not tell. African Journal of AIDS Research. 2014;13(2):189-95.

27. Mutumba M, Bauermeister JA, Musiime V, Byaruhanga J, Francis K, Snow RC, et al. Psychosocial challenges and strategies for coping with HIV among adolescents in Uganda: a qualitative study. AIDS Patient Care STDS. 2015;29(2):86-94.

28. Mattes D. “Life is not a rehearsal, it's a performance”: An ethnographic enquiry into the subjectivities of children and adolescents living with antiretroviral treatment in northeastern Tanzania. Children and Youth Services Review. 2014;45:28-37.

29. Mburu G, Hodgson I, Kalibala S, Haamujompa C, Cataldo F, Lowenthal ED, et al. Adolescent HIV disclosure in Zambia: barriers, facilitators and outcomes. J Int AIDS Soc. 2014;17:18866.

30. Inzaule SC, Hamers RL, Kityo C, Rinke de Wit TF, Roura M. Long-Term Antiretroviral Treatment Adherence in HIV-Infected Adolescents and Adults in Uganda: A Qualitative Study. PLoS One. 2016;11(11):e0167492.

31. Coetzee B, Kagee A, Bland R. Barriers and facilitators to paediatric adherence to antiretroviral therapy in rural South Africa: a multi-stakeholder perspective. AIDS Care. 2015;27(3):315-21.

32. Hornschuh S, Laher F, Makongoza M, Tshabalala C, Kuijper LDJ, Dietrich J. Experiences of HIV-Positive Adolescents and Young Adults in Care in Soweto, South Africa. Journal of HIV/AIDS & Social Services. 2014;13(4):420-35.

33. Wolf HTH-F, B., L.; Bukusi, E., B; Kawango, E., A; Cohen, A., R.; Auerswald, C., L. “It is all about the fear of being discriminated

[against]...the person suffering from HIV will not

be accepted”: a qualitative study exploring the

reasons for loss to follow-up among HIV-positive

youth in Kisumu, Kenya. BMC Public Health. 2014;14(1154):<http://www.biomedcentral.com/1471-2458/14/1154>.

34. Cange CW, LeBreton M, Billong S, Saylors K, Tamoufe U, Papworth E, et al. Influence of stigma and homophobia on mental health and on the uptake of HIV/sexually transmissible infection services for Cameroonian men who have sex with men. Sex Health. 2015;12(4):315-21.

35. Nakanwagi S, Matovu JK, Kintu BN, Kaharuza F, Wanyenze RK. Facilitators and Barriers to Linkage to HIV Care among Female Sex Workers Receiving HIV Testing Services at a Community-Based Organization in Periurban Uganda: A Qualitative Study. J Sex Transm Dis. 2016;2016:7673014.

36. Kennedy CE, Baral SD, Fielding-Miller R, Adams D, Dludlu P, Sithole B, et al. "They are human beings, they are Swazi": intersecting stigmas and the positive health, dignity and prevention needs of HIV-positive men who have sex with men in Swaziland. J Int AIDS Soc. 2013;16 Suppl 3:18749.

37. Saleem HT, Mushi D, Hassan S, Bruce RD, Cooke A, Mbwambo J, et al. "Can't you initiate me here?": Challenges to timely initiation on antiretroviral therapy among methadone clients in Dar es Salaam, Tanzania. Int J Drug Policy. 2016;30:59-65.

38. Mtetwa S, Busza J, Chidiya S, Mungofa S, Cowan F. "You are wasting our drugs": health service barriers to HIV treatment for sex workers in Zimbabwe. BMC Public Health. 2013;13:698.

39. Guise A, Rhodes T, Ndimbii J, Ayon S, Nnaji O. Access to HIV treatment and care for people who inject drugs in Kenya: a short report. AIDS Care. 2016;28(12):1595-9.

40. Parsons JA, Bond VA, Nixon SA. 'Are We Not Human?' Stories of Stigma, Disability and HIV from Lusaka, Zambia and Their Implications for Access to Health Services. PLoS One. 2015;10(6):e0127392.

41. Yoshida K, Hanass-Hancock J, Nixon S, Bond V. Using intersectionality to explore experiences of disability and HIV among women and men in Zambia. Disability and Rehabilitation. 2014;36(25):2161-8.

42. Mendelsohn JB, Rhodes T, Spiegel P, Schilperoord M, Burton JW, Balasundaram S, et al. Bounded agency in humanitarian settings: a qualitative study of adherence to antiretroviral therapy among refugees situated in Kenya and Malaysia. Soc Sci Med. 2014;120:387-95.
