# Supplementary material for: Being HIV positive and staying on antiretroviral therapy in Africa: A qualitative systematic review and theoretical model
Source: PLoS One. 2019 Jan 10;14(1):e0210408. doi: 10.1371/journal.pone.0210408 (PMC6328200; doi:10.1371/journal.pone.0210408)
Supplement: S3 Evidence Annex — (DOCX) [file pone.0210408.s009.docx]

| **Theme 3: HIV is highly stigmatized and HIV-positive people need a lot of support in order to cope** | | | | |
| --- | --- | --- | --- | --- |
| Sub-themes | Codes | Sub-code | Illustrative quote(s) | Supporting papers |
| **HIV Stigma and discrimination undermines sense of belonging which impairs adherence and engagement in care** | HIV stigma is related to promiscuity, infectiousness, sickness and death |  | “The stigma of HIV arises in part because people in the society associate HIV infection with promiscuity and hence considers it shameful, sinful, and even a punishment from God"(1)  “They recounted stories of family members not allowing patients to use the same utensils, not wanting to eat food prepared by someone with HIV, and not wanting to touch the body of an HIV-positive family member prior to burial….Providers and patients agreed that people with HIV are harshly judged, and that HIV is seen differently than other chronic illnesses, like cancer, in which the person with the disease is not blamed.” (2) | (1-7) |
|  | HIV Stigma results in judgement and discrimination by others | Discrimination can may come from; peers, partners, employers, relatives, educators, community or household members | “Along with the sicknesses participants experienced, was also the reaction from family and community members. Due to participants’ sickly appearance and weight loss, family and community members were described as running away from them or avoiding them all together.”(8)  At home, acts of discrimination included separation of plates, utensils, and shower basins, as well as taunts about their status. A few respondents reported unequal treatment such as enrollment in inferior schools compared to non-HIV- infected children in the household and not being allowed to play with non-HIV-infected siblings.(5) | (2, 3, 5, 8-12) |
|  |  | Discrimination can lead to loss of support | “Other informants reported being shamed and excluded by their families or their spouse’s families and being denied material – especially food – support.” (13) | (13-18) |
|  |  | Discrimination can affect economic opportunities | “ ..stigma could take economic forms (for example, no longer being able to sell foods at a market or being financially exiled by spouses or family members..”(7) | (4, 7, 8, 16, 17) |
|  | HIV stigma can make people feel ashamed and blame themselves |  | “ Several women described sitting in social settings while friends, who were unaware of their HIV status, ridiculed and mocked other women who were known to be HIV- infected using labels such as: ‘‘ruined one,’’ ‘‘hit,’’ ‘‘de- valued,’’ ‘‘rotten,’’ ‘‘empty,’’ ‘‘promiscuous,’’ ‘‘sinful,’’ ‘‘a hooligan’’ (wahuni), ‘‘a prostitute,’’ or ‘‘the one that stepped on a wire.’’ These comments left respondents panicked about their fate (should their HIV serostatus become known) and distraught about how to reconcile their view of themselves with an altered, stigmatizing construction.” (7)  Resigning from the clinic may be one form of internalized stigma, but there are other ways in which PWD/HIV+ take up othering attitudes. One participant recounted blaming himself for becoming HIV-positive (3) | (2-4, 7, 19, 20) |
|  | HIV positive people may feel isolated and depressed |  | “Some PLWHA isolated themselves following their diagnosis due to anticipated stigma” (8)  “Many MSM said that living with a stigmatized sexual identity and a challenging, stigmatized disease led to feelings of depression as well as self-stigma or shame.” (21) | (2, 4, 8, 17, 21) |
|  | Loss of support systems, hopelessness and depression may impair ART adherence and engagement in care |  | “Patients and providers discussed a withdrawal from social support as a consequence of internal stigma and denial. Participants saw this withdrawal as leading people to suffer alone, rather than to seek treatment and support.” (2)  “On the other hand, clients who delayed linkage to care clearly had difficulty  coping with prospect of being HIV-positive. Some were clearly more focused on the potentially negative outcomes of their illness rather than on how care seeking could facilitate their well-being.”(22)  “Three women reported ceasing PMTCT treatments because they felt they had no one who cared for them and they no longer wanted to live.”(7) | (1, 2, 7, 22) |
| **HIV-positive people fear inadvertent disclosure and may disengage or skip doses if confidentiality is at risk** | HIV-positive people will work hard to maintain HIV status a secret |  | “Unique to this group of women was the extent to which they strategized to keep their positive status from the watchful eyes of husbands, neighbors, and the kinship group”(16)  “Lack of privacy was a particular challenge for patients working in a private home or organization…... They had problems with finding a place to keep and take their medications; they thought that they had to hide their pills from employers and colleagues. Patients felt that they might be dismissed from their job if employers learned of their status” (23)  “Some CTC clients travelled long distances to avoid seeing people that they knew at CTC facilities in their communities”(24)  “Both anticipated and experienced discrimination clearly affected children’s retention in care. Caregivers refrained from disclosing their own or a child’s status out of fear of re- percussions, giving numerous examples of discrimination they had witnessed.”(25)  “Many of these stories were cautionary tales of other people in their community, rather than about themselves personally, to explain why disclosure was challenging”(2) | (2, 8, 15, 16, 22-25) |
|  | It is hard to take ART If household members/partner/schoolmates are not informed |  | “The household context plays an important role in this. Second, if a patient decides not to disclose to some or all household members, a burden of secrecy is created, which inhibits the development of HIV/AIDS competence”(18)  “Fear of involuntary disclosure, and resultant lack of disclosure directly affected poor adherence with medication in school”(26)  The majority of the problems were associated with privacy for storing and taking medications within the boarding school. Further, students sometimes had conflicts with their medication schedule and their class schedule: the teachers would not allow them to leave the class so that they are forced to take their medication in front of other students and had to avoid inadvertently disclosing their status. These situations led to students becoming uncomfortable, isolated, and non-adherent. (6) | (1, 5, 6, 14, 15, 18, 25-28) |
|  | If clinic staff, location, layout or method of integration compromises confidentiality patients may disengage |  | “Another huge source of discontent among HIV/AIDS sufferers was that their condition became ostensibly public in the process of integration and they feared stigma and loss of confidentiality”(29)  “Women shared accounts of providers being insensitive to their desire to keep their HIV status confidential. One woman described how she was waiting on a bench located a short distance from ‘‘the PMTCT room’’ so that if a relative or friend saw her at the facility she would not be ‘‘caught’’ near the stigmatized room. When the provider finished with a client, she called the woman’s name loudly across the courtyard, alerting all within earshot (and knowledgeable of the provider’s HIV-related professional role) of the woman’s status.” (7)  “A major stigma-related barrier mentioned by patients and providers was a fear of seeing other patients and providers at their local clinic who resided in the same community and might gossip about their serostatus. This fear led people to hide their serostatus, even from immediate family, presenting challenges for adherence and utilization.”(2) | (2, 4, 7, 16, 22, 26, 29, 30) |

1. Ngarina MP, R.; Kilewo, C.; Beberfeld, G.; Ekstrom, A., M. Reasons for poor adherence to antiretroviral therapy postnatally in HIV-1 infected women treated for their own health: experiences from the Mitra Plus study in Tanzania. BMC Public Health. 2013;13(450):<http://www.biomedcentral.com/1471-2458/13/450>.

2. Bogart LM, Chetty S, Giddy J, Sypek A, Sticklor L, Walensky RP, et al. Barriers to care among people living with HIV in South Africa: contrasts between patient and healthcare provider perspectives. AIDS Care. 2013;25(7):843-53.

3. Parsons JA, Bond VA, Nixon SA. 'Are We Not Human?' Stories of Stigma, Disability and HIV from Lusaka, Zambia and Their Implications for Access to Health Services. PLoS One. 2015;10(6):e0127392.

4. Layer EH, Kennedy CE, Beckham SW, Mbwambo JK, Likindikoki S, Davis WW, et al. Multi-level factors affecting entry into and engagement in the HIV continuum of care in Iringa, Tanzania. PLoS One. 2014;9(8):e104961.

5. Mutumba M, Bauermeister JA, Musiime V, Byaruhanga J, Francis K, Snow RC, et al. Psychosocial challenges and strategies for coping with HIV among adolescents in Uganda: a qualitative study. AIDS Patient Care STDS. 2015;29(2):86-94.

6. Mutwa PR, Van Nuil JI, Asiimwe-Kateera B, Kestelyn E, Vyankandondera J, Pool R, et al. Living situation affects adherence to combination antiretroviral therapy in HIV-infected adolescents in Rwanda: a qualitative study. PLoS One. 2013;8(4):e60073.

7. McMahon SA, Kennedy CE, Winch PJ, Kombe M, Killewo J, Kilewo C. Stigma, Facility Constraints, and Personal Disbelief: Why Women Disengage from HIV Care During and After Pregnancy in Morogoro Region, Tanzania. AIDS and Behavior. 2016;21(1):317-29.

8. Okoror TA, Falade CO, Olorunlana A, Walker EM, Okareh OT. Exploring the cultural context of HIV stigma on antiretroviral therapy adherence among people living with HIV/AIDS in southwest Nigeria. AIDS Patient Care STDS. 2013;27(1):55-64.

9. Mattes D. “Life is not a rehearsal, it's a performance”: An ethnographic enquiry into the subjectivities of children and adolescents living with antiretroviral treatment in northeastern Tanzania. Children and Youth Services Review. 2014;45:28-37.

10. Inzaule SC, Hamers RL, Kityo C, Rinke de Wit TF, Roura M. Long-Term Antiretroviral Treatment Adherence in HIV-Infected Adolescents and Adults in Uganda: A Qualitative Study. PLoS One. 2016;11(11):e0167492.

11. Mburu G, Hodgson I, Kalibala S, Haamujompa C, Cataldo F, Lowenthal ED, et al. Adolescent HIV disclosure in Zambia: barriers, facilitators and outcomes. J Int AIDS Soc. 2014;17:18866.

12. Kawuma R, Bernays S, Siu G, Rhodes T, Seeley J. ‘Children will always be children’: Exploring perceptions and experiences of HIV-positive children who may not take their treatment and why they may not tell. African Journal of AIDS Research. 2014;13(2):189-95.

13. Fielding-Miller RM, Z.; Adams, D.; Baral, S.; Kennedy, C. “There is hunger in my community”: a qualitative study of food security as a cyclical force in sex work in Swaziland. BMC Public Health. 2014;14(79):<http://www.biomedcentral.com/1471-2458/14/79>.

14. Sikweyiya YM, Jewkes R, Dunkle K. Impact of HIV on and the constructions of masculinities among HIV-positive men in South Africa: implications for secondary prevention programs. Glob Health Action. 2014;7:24631.

15. Maeri I, El Ayadi A, Getahun M, Charlebois E, Akatukwasa C, Tumwebaze D, et al. "How can I tell?" Consequences of HIV status disclosure among couples in eastern African communities in the context of an ongoing HIV "test-and-treat" trial. AIDS Care. 2016;28 Suppl 3:59-66.

16. Elwell K. Social and Structural Factors Affecting Women’s Participation in prevention of mother to child transmission(PMTCT) programs in Malawi. Antrhopology. 2015;Doctor of Philosophy:210.

17. Asgary R, Antony S, Grigoryan Z, Aronson J. Community perception, misconception, and discord regarding prevention and treatment of infection with human immunodeficiency virus in Addis Ababa, Ethiopia. Am J Trop Med Hyg. 2014;90(1):153-9.

18. Masquillier C, Wouters E, Mortelmans D, van Wyk B. On the road to HIV/AIDS competence in the household: building a health-enabling environment for people living with HIV/AIDS. Int J Environ Res Public Health. 2015;12(3):3264-92.

19. Wouters E, De Wet K. Women's experience of HIV as a chronic illness in South Africa: hard-earned lives, biographical disruption and moral career. Sociol Health Illn. 2016;38(4):521-42.

20. Mburu GR, M.; Siu, G.; Bitira, D.; Skovdal, M.; Holland, P. Intersectionality of HIV stigma and masculinity in eastern Uganda: implications for involving men in HIV programmes. BMC Public Health. 2014;14(1061):<http://www.biomedcentral.com/1471-2458/14/1061>.

21. Kennedy CE, Baral SD, Fielding-Miller R, Adams D, Dludlu P, Sithole B, et al. "They are human beings, they are Swazi": intersecting stigmas and the positive health, dignity and prevention needs of HIV-positive men who have sex with men in Swaziland. J Int AIDS Soc. 2013;16 Suppl 3:18749.

22. Naik R. Linkage to care following

home-based HIV counseling and testing: a mixed methods study in rural South Africa: University of Boston; 2013.

23. Thorne C, Bezabhe WM, Chalmers L, Bereznicki LR, Peterson GM, Bimirew MA, et al. Barriers and Facilitators of Adherence to Antiretroviral Drug Therapy and Retention in Care among Adult HIV-Positive Patients: A Qualitative Study from Ethiopia. PLoS ONE. 2014;9(5).

24. Ware NC, Wyatt MA, Geng EH, Kaaya SF, Agbaji OO, Muyindike WR, et al. Toward an understanding of disengagement from HIV treatment and care in sub-Saharan Africa: a qualitative study. PLoS Med. 2013;10(1):e1001369; discussion e.

25. Busza J, Dauya E, Bandason T, Mujuru H, Ferrand RA. "I don't want financial support but verbal support." How do caregivers manage children's access to and retention in HIV care in urban Zimbabwe? J Int AIDS Soc. 2014;17:18839.

26. Wolf HTH-F, B., L.; Bukusi, E., B; Kawango, E., A; Cohen, A., R.; Auerswald, C., L. “It is all about the fear of being discriminated

[against]...the person suffering from HIV will not

be accepted”: a qualitative study exploring the

reasons for loss to follow-up among HIV-positive

youth in Kisumu, Kenya. BMC Public Health. 2014;14(1154):<http://www.biomedcentral.com/1471-2458/14/1154>.

27. Hatcher AM, Stockl H, Christofides N, Woollett N, Pallitto CC, Garcia-Moreno C, et al. Mechanisms linking intimate partner violence and prevention of mother-to-child transmission of HIV: A qualitative study in South Africa. Soc Sci Med. 2016;168:130-9.

28. Kim MH, Zhou A, Mazenga A, Ahmed S, Markham C, Zomba G, et al. Why Did I Stop? Barriers and Facilitators to Uptake and Adherence to ART in Option B+ HIV Care in Lilongwe, Malawi. PLoS One. 2016;11(2):e0149527.

29. Braga B, M., T. “Death is Destiny”: Sovereign Decisions and the Lived Experience of HIV/AIDS and Biomedical Treatment in Central Mozambique: University at Buffalo, State University of New York; 2013.

30. Guise A, Rhodes T, Ndimbii J, Ayon S, Nnaji O. Access to HIV treatment and care for people who inject drugs in Kenya: a short report. AIDS Care. 2016;28(12):1595-9.
