# Supplementary material for: Being HIV positive and staying on antiretroviral therapy in Africa: A qualitative systematic review and theoretical model
Source: PLoS One. 2019 Jan 10;14(1):e0210408. doi: 10.1371/journal.pone.0210408 (PMC6328200; doi:10.1371/journal.pone.0210408)
Supplement: S4 Evidence Annex — (DOCX) [file pone.0210408.s010.docx]

| **Theme 5: Plethora of alternative and conflicting explanations about HIV and ART** | | | | |
| --- | --- | --- | --- | --- |
| Sub-themes | Codes | Sub-code (1) | Illustrative quote(s) | Supporting papers |
| **Alternative discourses about HIV & ART** | There are many different explanations for the causes and treatment of HIV |  | “In Tanzania, biomedical explanations of HIV/AIDS coexist alongside explanations that attribute its symptoms to witchcraft, ritual pollution, or divine retribution for immorality, all with very different consequences for prevention and treatment strategies. Uncertainty is what prevails, both within the medical realm and within the wider discourses about HIV/AIDS.” (1)  “A second factor influencing treatment adherence is the presence of plausible alternative interpretations of sickness. What physicians diagnose as AIDS-related diseases, diviners and Christian healers may well interpret as signs of bewitchment.” (2) | (1-5) |
|  | There are many different sources of information about HIV which may influence patients decisions |  | “Different specialized authorities—diviners, Christian healers, politicians, general practitioners, and HIV clinics—act as guarantors for the status of fact” (2)  “A third of the FSWs mentioned that they were using herbs as a substitute for ARVs and therefore saw no need of being registered in an HIV clinic. This was done following negative advice from fellow sex workers.”(6)  “Major sources of information included mass media, “kebele” community groups, neighbors and friends, churches and community workers….The role of the church and religious leaders was acknowledged as influential in spreading information…. Religious leaders are respected, and given authority over health issues.” (5) | (2, 5-7) |
|  | Attending traditional healers is often favoured over the biomedical health system |  | “Using traditional healers seems to have had been a preferred option by some of the men.”(8)  “Most clients also reported that they would not use traditional medicine for HIV and that they were aware that traditional medicine and ARVs should never be mixed. However, a few clients did report not seeking care due to a preference for traditional medicine.”(9) | (6-10) |
|  | Ideologies which offer a cure are particularly desirable and may influence engagement in HIV care |  | Patients preferred complete cure from HIV/AIDS with the holy water treatment rather than taking pills throughout their life. Interviewees and focus group members reported discontinuation of ART treatment in those being baptized with holy water. (4)  When we interviewed Bophelo, he had recently been diagnosed with HIV and mentioned having asked someone to prepare traditional medicine for him to cure the HIV…” (8)  Several clients of spiritual healing services also delayed linking to care because they had faith that God would either cure them or stop disease progression. (7) | (4, 5, 7, 8, 11) |
| **Side-effects undermine the message of better health on ART** | Side-effects create doubt about ART, particularly for the well |  | For several participants, initial side effects on ART were serious and caused doubts about what they had been told. (12)  Side effects made some women question the efficacy of ART. One woman, for instance, felt that the medicine made her feel so weak that she was unable to do basic household chores. (13)  Patients who experienced unanticipated and/or intolerable side effects, like nightmares and psychosis, missed doses or discontinued therapy altogether. This was particularly likely among patients who had started ART when asymptomatic as they considered the medications had worsened their health. (4)  While ARVs can facilitate the process of re-establishing normality in life, they may also be experienced as uncomfortable and even harmful. When the long-term side effects of the drugs become visible, this often leads to increased dissatisfaction with the treatment, and some consider dropping out.(1) | (4, 11-13) |
|  | Some fear negative ART effects and fail to link |  | “They avoided the ARVs by not going to the clinic while some opted to wait until they got other jobs because of the perception that ARVs could weaken them and disrupt their work.” (6)  Some people in Manica believed ARVs could bring or accelerate death to people living with HIV, possibly relating late initiation into ARVs and subsequent deaths. However, with the increase of treatment coverage and widespread patients’ recovery, those beliefs seem to be receding. (3) | (3, 6) |
| **Scientific uncertainty creates confusion and doubt** |  |  | “The suddenness and promptness of recommended ART-initiation contrasted sharply with previous guidelines, when one was taken through a series of preparatory stages and given, they said, ample time to reflect on the life- long decision. This was particularly confusing for women who had been being monitored for CD4+ cell count and then were suddenly told this count did not matter. This might have led to some of them to hesitate to start ART.”(14)  “This absence of bio- medicine’s memory of its past mistakes and revisions of opinion has created uncertainty about the reliability of scientific information and combines with conspiracy theories about the hidden motives behind medical interventions. The controversies around the potential harm and benefits of nevirapine to prevent mother-to-child transmission of HIV in South Africa are a prime example. More recently, education on condoms is being watered down by the latest studies, which show it is safe to drop condom use for couples on ARVs where both partners’ viral levels are undetectable. There are few clear messages in the field of AIDS, no unequivocal agreements, no clearly drawn enemy lines.”(1) | (1, 2, 14, 15) |
| **People choose ideology which suits them best** |  |  | “Knowledge about sickness does not imply unswerving commitment to one set of beliefs—be it witchcraft, pollution, spirit possession, or viral infections. New concepts and beliefs are constantly tried out and added on, and practical considerations often outweigh explanatory consistency.” (2) | (1, 2) |

1. Beckmann N. Responding to medical crises: AIDS treatment, responsibilisation and the logic of choice. Anthropol Med. 2013;20(2):160-74.

2. Niehaus I. Treatment literacy, therapeutic efficacy, and antiretroviral drugs: notes from Bushbuckridge, South Africa. Med Anthropol. 2014;33(4):351-66.

3. Braga B, M., T. “Death is Destiny”: Sovereign Decisions and the Lived Experience of HIV/AIDS and Biomedical Treatment in Central Mozambique: University at Buffalo, State University of New York; 2013.

4. Thorne C, Bezabhe WM, Chalmers L, Bereznicki LR, Peterson GM, Bimirew MA, et al. Barriers and Facilitators of Adherence to Antiretroviral Drug Therapy and Retention in Care among Adult HIV-Positive Patients: A Qualitative Study from Ethiopia. PLoS ONE. 2014;9(5).

5. Asgary R, Antony S, Grigoryan Z, Aronson J. Community perception, misconception, and discord regarding prevention and treatment of infection with human immunodeficiency virus in Addis Ababa, Ethiopia. Am J Trop Med Hyg. 2014;90(1):153-9.

6. Nakanwagi S, Matovu JK, Kintu BN, Kaharuza F, Wanyenze RK. Facilitators and Barriers to Linkage to HIV Care among Female Sex Workers Receiving HIV Testing Services at a Community-Based Organization in Periurban Uganda: A Qualitative Study. J Sex Transm Dis. 2016;2016:7673014.

7. Layer EH, Kennedy CE, Beckham SW, Mbwambo JK, Likindikoki S, Davis WW, et al. Multi-level factors affecting entry into and engagement in the HIV continuum of care in Iringa, Tanzania. PLoS One. 2014;9(8):e104961.

8. Sikweyiya YM, Jewkes R, Dunkle K. Impact of HIV on and the constructions of masculinities among HIV-positive men in South Africa: implications for secondary prevention programs. Glob Health Action. 2014;7:24631.

9. Naik R. Linkage to care following

home-based HIV counseling and testing: a mixed methods study in rural South Africa: University of Boston; 2013.

10. Appelbaum Belisle H, Hennink M, Ordonez CE, John S, Ngubane-Joye E, Hampton J, et al. Concurrent use of traditional medicine and ART: Perspectives of patients, providers and traditional healers in Durban, South Africa. Glob Public Health. 2015;10(1):71-87.

11. McMahon SA, Kennedy CE, Winch PJ, Kombe M, Killewo J, Kilewo C. Stigma, Facility Constraints, and Personal Disbelief: Why Women Disengage from HIV Care During and After Pregnancy in Morogoro Region, Tanzania. AIDS and Behavior. 2016;21(1):317-29.

12. Russell S, Namukwaya S, Zalwango F, Seeley J. The Framing and Fashioning of Therapeutic Citizenship Among People Living With HIV Taking Antiretroviral Therapy in Uganda. Qual Health Res. 2016;26(11):1447-58.

13. Kim MH, Zhou A, Mazenga A, Ahmed S, Markham C, Zomba G, et al. Why Did I Stop? Barriers and Facilitators to Uptake and Adherence to ART in Option B+ HIV Care in Lilongwe, Malawi. PLoS One. 2016;11(2):e0149527.

14. Mbonye M, Seeley J, Nalugya R, Kiwanuka T, Bagiire D, Mugyenyi M, et al. Test and treat: the early experiences in a clinic serving women at high risk of HIV infection in Kampala. AIDS Care. 2016;28 Suppl 3:33-8.

15. Katirayi L, Namadingo H, Phiri M, Bobrow EA, Ahimbisibwe A, Berhan AY, et al. HIV-positive pregnant and postpartum women's perspectives about Option B+ in Malawi: a qualitative study. Journal of the International AIDS Society. 2016;19(1).
