# Supplementary material for: Being HIV positive and staying on antiretroviral therapy in Africa: A qualitative systematic review and theoretical model
Source: PLoS One. 2019 Jan 10;14(1):e0210408. doi: 10.1371/journal.pone.0210408 (PMC6328200; doi:10.1371/journal.pone.0210408)
Supplement: S5 Evidence Annex — (DOCX) [file pone.0210408.s011.docx]

| **Theme 5:** “Bad patients” are an unhelpful construct of an authoritarian health | | | | |
| --- | --- | --- | --- | --- |
| Sub-themes | Codes | Sub-code | Illustrative quote(s) | Supporting papers |
| Authoritarian health system | The biomedical approach is authoritarian, hierarchical and paternalistic |  | I suggest the interface of a political culture marked by an “authoritarian mode” and strict hierarchies reinforced by the authoritative traits and hierarchic features of biomedicine itself seem to be mutually influencing and reinforcing each other, affecting provision of health-care within the National Health Services (NHS) in central Mozambique. (1)  The teacher-student-like quality of counselling must also be seen in the context of counsellors’ authority as HIV professionals. As generally educated people with specialised training in HIV and AIDS, the counsellors occupy positions of authority, which are also derived from their connection to the medical field. Clients in Kampala often referred to their counsellor as musawo – a Luganda term for doctor, broadly used for any health worker. In general, health workers in Uganda adopt a highly authoritarian attitude. Rose’s frustration over clients who ‘do not listen’ can be seen as a frustration of her inability to establish herself as the authority, she believes she ought to be. (2)  Women generally perceived their social status below that of the provider. This has been described in another Tanzanian nursing study , and reflects wider gender and cultural norms, respect for those in positions of authority and the inherent and assumed power structure, although power differentials between patients and health service providers are not uncommon in developed world settings.(3) | (1-4) |
|  | Power imbalance between HCWs and HIV positive people | Health care workers hold all power at clinic | Nurses are in a hugely powerful position compared to their rural, poor and often less educated patients. Their power is clear in many ways; they wear uniforms, which signal their biomedical authority, reprimand patients when they fail to follow directions, have control over queues and order of access to service, and are responsible for administering drugs. (4) | (1, 4, 5) |
|  |  | Patients see HCWs as powerful and try to please them | Terminology used by interviewees and community members to describe providers reflected the attributed power: terms such as ‘experts’, ‘specialists’ and ‘experienced’ were frequently used. (3)  Patients are acutely aware of their dependence on the goodwill of doctors and nurses, which has been heightened by the provision of free antiretroviral treatment. This dependency has stifled the emergence of empowered patients and silenced the voices of activists. (5)  In an effort to appear open and honest, especially in relation to less-than-perfect adherence, patients offer explanations to nurses before even being asked. This not only highlights the authority and power of nurses in their ART treatment, but also the fear patients have of doing something wrong and being reprimanded and how they carefully navigate their interactions with the nurses.(4) | (2-6) |
|  |  | HCWs may abuse their powerful role and disrespect patients | “Patients sometimes felt disrespected by staff, who were described as “short-tempered” and “scolding” in some instances, rather than empathetic and caring. Patients told of staff (usually in public clinics) who took frequent “tea breaks,” made personal calls during work hours, or socialized among themselves while patients waited. Patients sometimes felt helpless in finding out information about appointment delays.”(7)  “Harsh treatment typically referred to behavior perceived by patients to be rude and/or rejecting. For example, interviewees reported being spoken to ‘‘roughly’’ or feeling that the clinic staff ‘‘didn’t care.’’ ‘‘Shouting’’ and ‘‘bad language’’ were cited. (8)  “Many study participants endured verbal abuse and disrespectful treatment by providers at CTC facilities because accepting this mistreatment was the only way to receive ART.” (9) | (1, 7-9) |
|  |  | Guidelines may be presented as rules | Information from providers was referred to as ‘conditions’, ‘directions’, and ‘instructions’, while phrases like being ‘ordered’ by health workers, or ‘violating the conditions’ were also common (8).  The rules stipulate that clients taking ART have to take their medicine every day at the same time, come for clinical appointments on time, eat a balanced diet, avoid smoking, drinking, unprotected sex and overworking, discuss plans to have children with their counsellor and doctor, disclose their status and maintain a positive attitude. The rules reflect how Positive Living is reconfigured in the context of ART: from an ethical self- practice to a set of lifestyle choices people living with HIV are urged to follow in order to successfully ‘save’ their lives with ARTs… Successfully reciting the rules thus functions as a way for clients to demonstrate that they are capable of behaving like a ‘good ART client’. (2).  “Moreover, the biomedical system is strongly hierarchical, with medical professionals carefully guarding their expert knowledge. Patients are generally regarded as ignorant and irrational, in need of being educated and disciplined through counselling and education sessions that repeat standard scripts of managing their conditions through ‘living positively’. This includes embracing their HIV-positive identity, following the doctor’s advice, and striving to be morally good people who neither drink nor smoke nor engage in illicit sex, and who help fellow sufferers through home-based care and peer counselling.”(5) | (1, 2, 5, 8) |
|  |  | People work hard to carry favour with HCWs and facilitate care | “This example illustrates how clients may attempt to demonstrate compliance to avoid getting into discussions about sexual behaviours that conflict with ‘the rules.’”(2)  “Our research identified many ways in which patients ‘signal’ their goodness and deservingness of treatment or their respect for the medical establishment to their nurses and other healthcare givers. Performing within the ‘good patient persona’ is seen to be a way patients can influence their hospital experience by increasing their chance of receiving good services. Securing good care is particularly important in resource-poor settings where staff-time and medication are in short supply.”(4) | (2, 4) |
| Rules and responsibility | HIV-positive people are given and extensive list of adherence and 'positive living guidelines’ | Excellent attendance, adherence and obedience | “The social representation of a good ART patient includes being obedient to the nurses’ instructions, enthusiastic, patient about waiting in queues, and open. In the following quotation, a nurse explains her perception of a good patient, emphasizing the value of following directions, waiting quietly in the queue and being cheerful..”(4)  The message many patients recalled from their counseling was to take the medicine at the exact same time every single day, eat well and keep good hygiene, not to drink alcohol and the importance of monthly pill-refills. (10)  “Successfully reciting the rules thus functions as a way for clients to demonstrate that they are capable of behaving like a ‘good ART client’”(2)  Across all the interviews, on only two occasions was an health workers’ abuse of their authority reported, which was the indirect threat to withdraw treatment as a means of social control over a “difficult” patient. For example, a male participant described a fellow patient who had talked about his rights at the clinic. The health worker had told the patient “if you know more than us, go somewhere else, it will be easier for you.” None of the participants themselves had experienced this threat. One male participant said he had once missed an appointment, and was chastised for this, but that he understood why keeping appointments was important, and on that occasion, he received further counseling and was given the medication.(6) | (2, 4, 6, 10) |
|  |  | Learn HIV language and support the biomedical rationale | A client has to learn how to come to terms with their HIV diagnosis while they simultaneously are ushered into a complex system of healthcare and illness management. Some participants described being overwhelmed by having to learn all of the new “language,” including what CD4 count means, the importance of the viral load, and the basics of how the virus affected their body. (11)  “The main characteristics of this form of therapeutic citizenship were as follows: -Motivation to learn and acquire new knowledge…”(6) | (1, 6, 11, 12) |
|  |  | Reduce sexual activity and eliminate alcohol | Prominent messages absorbed by participants were as follows: first and foremost, to adhere to medication and set times each to take the pills; second, to eat nutritious food, boil drinking water, and stop alcohol consumption; and third, “responsible” sexual behavior. Regarding sexual behavior, messages targeted particularly at men were as follows: “do not be promiscuous,” “be faithful to your partner,” and “give up on multiple partners.” Messages targeted toward women were “abstain (if you can),” “reduce sexual activity,” “avoid pregnancies,” and “giving birth weakens health.”(6)  The counsellors warned clients that re-infection would make the treatment less effective. Jagwe also implied that ‘having too much sex’ could strain the clients’ immune system, thereby threatening treatment efficacy. In this way, counsellors invoke the authority of the medicine to persuade clients to restrict their sexual activity.(2)  “Drinking beer had been a source of pleasure, a means to overcome worries and/or a social activity to interact with others. But proper adherence to HIV treatment imposed restrictions on alcohol use and discouraged smoking, despite these being considered important activities that usually brought men together.”(13) | (2, 6, 12, 13) |
|  |  | Reduce and plan pregnancies | Nevertheless, if clients wish to have children, they are urged to first discuss it with their doctor and counsellor, to assess whether the woman needs to change ART regimen, whether she is well enough to bear a pregnancy to term and to discuss the possible risks of HIV transmission or re-infection. Despite this official policy, there were some counsellors who treated clients’ wishes to have children as morally suspect. The counsellor in the rural clinic claimed, ‘in fact they are not supposed to get pregnant’ (2)  Grounding their statements in biomedical risk discourse, these providers assume that HIV-positive women should not have more children because of the nature of their positive status. (14) | (2, 6, 14) |
|  |  | Eat healthy foods | Messages about eating a good diet, drinking clean water, and hygiene had also been absorbed into the language of self- management. (6)  Our informants were frequently advised at local clinics that they should eat “healthy foods” to manage their HIV infection. This advice was well received, and many informants talked about “healthy foods” when asked how they coped with their HIV status.(15)  Some others patients believed that they had to consume costly food items to take their HIV medications.(16) | (6, 15-18) |
|  |  | Do not stress | “An integral part of positive living includes the ability to acquire a calm state of mind and eliminate those circumstances that would cause worry.”(5)  “Respondents reported that health providers had counseled them to avoid worrying about their HIV status as it would lead to poor health outcomes such as poor drug response and early death.”(19) | (5, 6, 19, 20) |
|  |  | Embrace HIV positive status/ART and help others with HIV | “Peter is in many ways a model patient-citizen, who educates others and serves as an example. He had integrated scientific information on the drugs and the virus into his lay understanding of his condition and views the drugs as his lifeline – without them he would be dead. He scolds others who are lax in their adherence and gets angry when people voice their thoughts on stopping the treatment.”(5)  “The main characteristics of this form of therapeutic citizenship were as follows:  -Having a sense of responsibility for the health of  others; -Raising awareness and supporting other PLWH;  and, -Having a collective sense of belonging to a wider  community of PLWH.” | (5, 6) |
|  | Responsibility is placed on the HIV-positive person | HCWs often put all the responsibility of being adherent on the patient | ““From my weekly observations during 14 consecutive months (approx. 96 days) of counseling sessions in several districts and health units, counseling consisted mainly of transfer of information about HIV/AIDS and ART. It is assumed that patients in ART lack knowledge of what the implications of discontinuing treatment are, because if they knew, they would be adherent”…. “The counselor began by blaming the patient for not coming to the hospital, ignoring the possibility that there might be reasons for it. By saying “these drop-out patients are just like this ...” she is in fact considering “non-adherence” as a personal characteristic of the patient, essentializing and naturalizing it, while decontextualizing the conditions of his engagement with AIDS treatment.” (1)  Rather than responding with care, the constant warning to be ‘good’ patients and make the right choices – to eat healthy food, abstain from sex, avoid pregnancy, etc. – adds to the pressures and confusion patients already face and shifts the blame for treatment failure onto the patients’ shoulders.(5) | (1, 2, 5, 7, 12) |
|  |  | Biomedical model and counselling ignores social responsibilities and lived realities | “During the initial adherence counseling sessions that I observed the nurse slowly flipped through a cardboard slideshow on her desk showing how ‘good food’ acts as a shield protecting the body from the virus. ‘Good food’ in these diagrams included eggs, meat, beans, pineapples, and fish. Unfortunately, nearly all of these foods are either unavailable in this region (such as pineapples) or beyond the financial reach of most, including eggs, fish and especially meat. Fruit in particular was hard to come by in this tobacco growing region because of the reluctance of most land- owners to plant trees for temporary residents. Tenants are also only given land to grow tobacco, and are rarely provided any space to grow vegetables, or beans. Therefore, rather than reassure caregivers, this slideshow had the overall effect of creating feelings of anxiety and frustration that they were failing to meet these expectations.” (18)  “PMTCT patients who are scolded for having additional children when HIV-positive reveal the tension in women who desire to provide a child to the family and lineage, yet at the same time are framed as poor mothers because they have exposed their future child to additional medical risk of contracting HIV.”(14)  “In this context, a number of social, economic and cultural factors shape clients’ behaviours in ways that are not easily compatible with, particularly, the rule of safe sex’(2)  This paper argues that both these approaches to responsibilisation fail to explain the lived experiences of HIV/AIDS among people living with HIV/AIDS (PLHA) in Tanzania, who respond ‘responsibly’ to their condition but not necessarily in ways that are understood by the biomedical authorities. The promotion of individual responsibility for health becomes highly problematic in settings where disease rates are high, poverty is widespread, and access to state health care diminishing. (5) | (2, 5, 6, 14, 18) |
|  | Peoples responses to ART guidelines and rules | Some people trust HCWs, respond well to authority and like the guidelines | “Health workers were viewed to have authoritative knowledge about HIV and participants had been willing to accept the instructions: They were not negotiated with health workers to arrive jointly at a treatment plan. Health workers’ authority, however, did not appear to have fashioned disciplined “docile bodies or undermined participants” sense of agency. On the contrary, they argued the framework had been a tool to support their own adjustment to life on ART; it gave them back a sense of control over their lives and had been a key factor in their recovery. “(6)  “The discourse of normalization positions people living with HIV and AIDS as chronic sufferers and invokes the need to manage their condition by adhering to their ARV medication, exercising, eating healthily and managing stress and anxiety. These constructions reflect an important public health discourse that is critical to the establishment of agency in dealing with illness. On one level, it was apparent that several participants had positioned HIV as an illness that had to be managed. …In the face of the very real public health threat presented by HIV and AIDS, the above discourse is critical in offering containment, reduction of fear and the enablement of a range of healthy behaviours and lifestyle changes.” (17) | (3, 6, 17, 21, 22) |
|  |  | Being able to adhere to guidelines helps some feel good about themselves | “These men derived a sense of importance and self-worth from occupying these caring positions in their communities, positions that are certainly counter to hegemonic ideals of masculinity. In the following extracts, we note the feeling of self-worth which, in turn, seems to have helped these men to regain self-respect and aided them in reconfiguring their masculinities into positive ones.” (22)  “Participants derived a great sense of achievement and pride from following the instructions and seeing the results of their efforts. They felt good about their competence in managing the condition..”(21) | (21, 22) |
|  |  | Stress, guilty, shame and anxiety if they are unable to abide | Rather than responding with care, the constant warning to be ‘good’ patients and make the right choices – to eat healthy food, abstain from sex, avoid pregnancy, etc. – adds to the pressures and confusion patients already face and shifts the blame for treatment failure onto the patients’ shoulders.(5)  Participants in our study commonly viewed their disengagement from care as shameful and blamed themselves for disobeying firm rules around HIV care, even if the decision to disengage was a direct result of abusive treatment by providers at these facilities. In addition, participants were ‘‘warned’’ by service providers not to repeat their behavior, which furthered feelings of guilt and shame.(23) | (5, 8, 11, 23) |
|  | Labelling and punishment | Inability to adhere to rules may result in being labelled | “Labeling diseased bodies as absentees and drop-outs produces categories of disciplined/adherent and undisciplined/non-adherent bodies as “kinds of people.” To be unable to fulfill HIV/AIDS care requirements was constructed as an individual characteristic, as illuminated by the statement that some AIDS sufferers “are drop-outs,” that is, a particular kind of people. “Adherence” is naturalized and essentialized and the specific social and historical complexities of AIDS patients’ lives are concealed.  The category of ‘drop-out” was institutionally established through the HIV/AIDS care and treatment intervention and new groups of people were constituted. The length of patients’ absence quantified, their medical records removed to separate specific cabinets somehow materialized the distinction between “disciplined adherent patients” on the one hand, and absentees and drop-outs on the other.” (1) | (1, 4, 24) |
|  |  | Labelling justifies poor treatment | “In return for access to lifesaving treatment and care, patients are expected to, and agree to, reciprocate with adherence to both medication and clinic appointments. The commitment entails a moral obligation; missed visits are thus a moral failing. Understanding the moral dimensions of the treatment relationship makes sense of patients’ shame, justifies ‘‘scolding’’ by providers, and explains the common usage of the term ‘‘defaulter’’ (i.e., someone who fails to repay a debt) to refer to patients who miss clinic appointments in sub-Saharan Africa.” (8)  “The label of “abandono” (drop-out) marks AIDS sufferers’ bodies and produces a stigmatized identity that, to borrow Biehl’s words (2007) is meant “both to explain their dying and to blame them for it.””(1) | (1, 8, 24) |
|  |  | Shame and punishment leads to disengagement and reluctance to return | Almost all participants in this study encountered negative experiences where they were shouted at, ‘‘scolded’’ or ‘‘punished’’ by one or more providers. Often, negative interactions occurred when a client disobeyed rules set by providers, most commonly arriving late or missing an appointment . When clients returned to the CTC on a day other than their assigned clinic day, they were often either denied services completely or forced to wait until the end of the day as punishment or ‘‘correction’’ for their behavior. Harsh and disrespectful treatment was the most common reason for CTC clients to disengage from care .(9)  Despite the potentially beneficial outcomes of the social representation of ‘good ART patients’, there are some negative potential outcomes that requires further attention. Patients who cannot perform within the ‘good patient persona’ must endure very trying and exhausting visits to the clinic. There are many reasons why patients cannot exhibit the signs of being a good patient, and these patients bear the brunt of nurse reprimands, which reduces quality of life and may discourage retuning to the clinic. (4)  Patient education in clinical settings for ART initiation in Africa has featured stern warnings about the lifelong commitment treatment represents, the meaning and importance of medication adherence, and the dire consequences of lapses. Patients’ feelings of shame at missing visits and fear of clinic staff’s negative response to absences may indicate just how seriously they have taken these warnings. Educational efforts intended to maximize the benefits of ART for patients may paradoxically be driving some away from care.(8)  “However, our findings indicate that these judgmental attitudes and hierarchical relationships contribute to disengagement and reluctance to return.”(23) | (1, 4, 5, 8, 9, 23, 25) |

1. Braga B, M., T. “Death is Destiny”: Sovereign Decisions and the Lived Experience of HIV/AIDS and Biomedical Treatment in Central Mozambique: University at Buffalo, State University of New York; 2013.

2. Rasmussen LM. Counselling clients to follow 'the rules' of safe sex and ARV treatment. Cult Health Sex. 2013;15 Suppl 4:S537-52.

3. Gourlay AW, A.; Birdthisle, I.; Mshana, G.; Michael, D.; Urassa, M. ‘‘It Is Like That, We Didn’t Understand Each Other’’: Exploring the Influence of Patient-Provider Interactions on Prevention of Mother-To-Child Transmission of HIV Service Use in Rural Tanzania. PLoS One. 2014;9(9).

4. Campbell C, Scott K, Skovdal M, Madanhire C, Nyamukapa C, Gregson S. A good patient? How notions of 'a good patient' affect patient-nurse relationships and ART adherence in Zimbabwe. BMC Infect Dis. 2015;15:404.

5. Beckmann N. Responding to medical crises: AIDS treatment, responsibilisation and the logic of choice. Anthropol Med. 2013;20(2):160-74.

6. Russell S, Namukwaya S, Zalwango F, Seeley J. The Framing and Fashioning of Therapeutic Citizenship Among People Living With HIV Taking Antiretroviral Therapy in Uganda. Qual Health Res. 2016;26(11):1447-58.

7. Bogart LM, Chetty S, Giddy J, Sypek A, Sticklor L, Walensky RP, et al. Barriers to care among people living with HIV in South Africa: contrasts between patient and healthcare provider perspectives. AIDS Care. 2013;25(7):843-53.

8. Ware NC, Wyatt MA, Geng EH, Kaaya SF, Agbaji OO, Muyindike WR, et al. Toward an understanding of disengagement from HIV treatment and care in sub-Saharan Africa: a qualitative study. PLoS Med. 2013;10(1):e1001369; discussion e.

9. Layer EH, Kennedy CE, Beckham SW, Mbwambo JK, Likindikoki S, Davis WW, et al. Multi-level factors affecting entry into and engagement in the HIV continuum of care in Iringa, Tanzania. PLoS One. 2014;9(8):e104961.

10. Axelsson JM, Hallager S, Barfod TS. Antiretroviral therapy adherence strategies used by patients of a large HIV clinic in Lesotho. J Health Popul Nutr. 2015;33:10.

11. Jones C. Between State and Sickness: The Social Experience of HIV/AIDS illness management and treatment in Grahamstown, South Africa [Dissertation]: Graduate School-New Brunswick

Rutgers, The State University of New Jersey; 2014.

12. Niehaus I. Treatment literacy, therapeutic efficacy, and antiretroviral drugs: notes from Bushbuckridge, South Africa. Med Anthropol. 2014;33(4):351-66.

13. Siu GE, Wight D, Seeley J. 'Dented' and 'resuscitated' masculinities: the impact of HIV diagnosis and/or enrolment on antiretroviral treatment on masculine identities in rural eastern Uganda. SAHARA J. 2014;11:211-21.

14. Elwell K. Social and Structural Factors Affecting Women’s Participation in prevention of mother to child transmission(PMTCT) programs in Malawi. Antrhopology. 2015;Doctor of Philosophy:210.

15. Fielding-Miller RM, Z.; Adams, D.; Baral, S.; Kennedy, C. “There is hunger in my community”: a qualitative study of food security as a cyclical force in sex work in Swaziland. BMC Public Health. 2014;14(79):<http://www.biomedcentral.com/1471-2458/14/79>.

16. Thorne C, Bezabhe WM, Chalmers L, Bereznicki LR, Peterson GM, Bimirew MA, et al. Barriers and Facilitators of Adherence to Antiretroviral Drug Therapy and Retention in Care among Adult HIV-Positive Patients: A Qualitative Study from Ethiopia. PLoS ONE. 2014;9(5).

17. Bhagwanjee A, Govender K, Reardon C, Johnstone L, George G, Gordon S. Gendered constructions of the impact of HIV and AIDS in the context of the HIV-positive seroconcordant heterosexual relationship. Journal of the International AIDS Society. 2013;16(1).

18. Sikstrom L. “Without the grandparents, life is difficult”: Social hierarchy and therapeutic trajectories for children living with HIV in rural Northern Malawi. Children and Youth Services Review. 2014;45:47-54.

19. Mutumba M, Bauermeister JA, Musiime V, Byaruhanga J, Francis K, Snow RC, et al. Psychosocial challenges and strategies for coping with HIV among adolescents in Uganda: a qualitative study. AIDS Patient Care STDS. 2015;29(2):86-94.

20. Wouters E, De Wet K. Women's experience of HIV as a chronic illness in South Africa: hard-earned lives, biographical disruption and moral career. Sociol Health Illn. 2016;38(4):521-42.

21. Russell S, Martin F, Zalwango F, Namukwaya S, Nalugya R, Muhumuza R, et al. Finding Meaning: HIV Self-Management and Wellbeing among People Taking Antiretroviral Therapy in Uganda. PLoS One. 2016;11(1):e0147896.

22. Sikweyiya YM, Jewkes R, Dunkle K. Impact of HIV on and the constructions of masculinities among HIV-positive men in South Africa: implications for secondary prevention programs. Glob Health Action. 2014;7:24631.

23. Layer EH, Brahmbhatt H, Beckham SW, Ntogwisangu J, Mwampashi A, Davis WW, et al. "I pray that they accept me without scolding:" experiences with disengagement and re-engagement in HIV care and treatment services in Tanzania. AIDS Patient Care STDS. 2014;28(9):483-8.

24. Wolf HTH-F, B., L.; Bukusi, E., B; Kawango, E., A; Cohen, A., R.; Auerswald, C., L. “It is all about the fear of being discriminated

[against]...the person suffering from HIV will not

be accepted”: a qualitative study exploring the

reasons for loss to follow-up among HIV-positive

youth in Kisumu, Kenya. BMC Public Health. 2014;14(1154):<http://www.biomedcentral.com/1471-2458/14/1154>.

25. Siu GE, Seeley J, Wight D. Dividuality, masculine respectability and reputation: how masculinity affects men's uptake of HIV treatment in rural eastern Uganda. Soc Sci Med. 2013;89:45-52.
