# Supplementary material for: Being HIV positive and staying on antiretroviral therapy in Africa: A qualitative systematic review and theoretical model
Source: PLoS One. 2019 Jan 10;14(1):e0210408. doi: 10.1371/journal.pone.0210408 (PMC6328200; doi:10.1371/journal.pone.0210408)
Supplement: S6 Evidence Annex — (DOCX) [file pone.0210408.s012.docx]

| **Theme 6: It is difficult and stressful attending public ART clinics** | | | | |
| --- | --- | --- | --- | --- |
| Sub-themes | Codes | Sub-code | Illustrative quote(s) | Supporting papers |
|  | Disrespectful health workers and poor communication |  | Some participants also expressed frustration at the way they were treated by CTC providers during their initial visit….. One of the most significant barriers to retention in CTC services was disrespectful and abusive treatment by service providers. Almost all participants in this study encountered negative experiences where they were shouted at, ‘‘scolded’’ or ‘‘punished’’ by one or more providers (1).  Harsh treatment typically referred to behavior perceived by patients to be rude and/or rejecting. For example, interviewees reported being spoken to ‘‘roughly’’ or feeling that the clinic staff ‘‘didn’t care.’’ ‘‘Shouting’’ and ‘‘bad language’’ were cited. At one site, references were made to threats that missed visits would result in being dropped from care. Experiences of harsh treatment left patients feeling hurt, angry, and humiliated (2).  Patients discussed how clinic staff did not respect the confidentiality of their serostatus, and of an abrupt style with inadequate communication about delays in test results and appointments, or the purposes of different queues (3).  Several women report negative interactions with some of the health care providers, claiming that nurses and other health care workers “sometimes do not talk to us nicely.” Participants admitted feeling “scared” coming to the clinic because of the manner in which they had been received by providers (4).  Further in my fieldwork several persons living with HIV/AIDS mentioned cases of  mistreatment that were indeed quite frequent, and I also observed other situations of patients’ abuse and humiliation (5). | (1-12) |
| It is difficult and stressful attending public ART health services | Inconvenient and inflexible health services | Rigid clinic policies | Interviewees reported objections to clinic policy (e.g., insistence on adherence to certain days and times of appointments) or some aspect of care organization (e.g., clinic schedules that made for long waiting times) (2).  Participants described frustration after traveling long distances for care only to be turned away if they arrived outside of clinic hours, if providers were too busy to serve them..(1) | (1, 2) |
|  |  | Limited operating hours and long waiting times | Patients had great concerns about the quality of care that they received, mostly centered on inconvenient clinic hours, long clinic queues, not being able to get appointments, and disrespect.(3)  “In contrast, they felt ignored by government clinic staff and frustrated with long wait-times, often missing work while waiting to be seen.”(6)  “Time spent queuing at the clinic is also a “cost”, taking time away from sourcing money.”(13)  “Furthermore, the on-site pharmacy appeared to have staffing shortages leading to exceptionally long waiting times.”(14) | (3, 6, 10, 13-17) |
|  |  | Requirement for a treatment supporter | One provider described a female methadone client who was delayed in initiating ART because she did not have anyone who could serve as her adherence supporter (18)  The four FSWs who were enrolled at government facilities noted that they were discouraged by the requirement to present a treatment supporter coupled with the long procedures for enrolment that involved several visits to the facility (19)  Requiring clients to have a treatment buddy, however, raises important financial concerns and becomes one of many economic negotiations patients make in the help-seeking process. For example, ARVs are free at the point of service, but treatment is not without cost. So, while ART is technically free, the readiness treatment maintenance processes can create a financial burden for participants.(20) | (18-20) |
|  | Navigating HIV health services is confusing and unpredictable |  | “Clients often encountered challenges during their initial visit and were told to leave and return on another day due to restricted opening hours, limited capacity for enrollment and shortages of providers. “ (1)  “Clinic visits are very unpredictable. These range from easy visits taking less than two hours to prolonged full day odysseys through multiple queues and interactions. On many clinic visits, patients simply do not know what is going on, whether there will be enough time for the nurses to see them and whether they will receive their pills.”(8)  So, it was just as AIDS sufferers told me, “they did not know the doors.” What they did not say was that if, for instance they did not hear their names and did not get to the right door in time, they would not be allowed to see the clinician that day. They could spend half the day waiting at the hospital, not to mention the time spent and the difficulties to get to the hospital, and nonetheless fail to get a clinical appointment. If they were “ lucky” they could just be reprimanded and still manage to have an appointment. I had noticed some patients getting anxious, sometimes even panicking if they did not hear their names being called out in Day-Hospital, but if it happened they would still get a consultation.(5)  Patients had difficulties navigating the hospital space. Patients told stories of waiting in the wrong queue or not being able to find the right queue prior to the clinic closing time, and then having to return. (3) | (1, 3, 5, 8) |
|  | Negative clinic experiences discourage engagement in HIV care |  | “Given the already challenging nature of attending the clinic - with regard to time, money, effort, and motivation, it is not difficult to imagine that clients who have poor experiences with the healthcare system feel disillusioned about the whole process, which may result in further delaying or not even re-attempting to seek care.”(16)  Emmanuel explained that he could not justify wasting his time and money on CTC visits when the quality of services was so low and he was in good health, so he disengaged from care and never returned.(1) | (1, 2, 5, 8, 16) |
| It’ s hard work being a health worker | Staff shortages and excessive work-load |  | Mistreatment of patients may signal the broader issue of resource limitations within government-funded health facilities. While many health care workers did not reveal the causes of their work stress, one theme that continued to emerge from interviews and clinical observations was the work burden faced by many health care providers within the government-run health care system. Often health care providers worked in circumstances in which staff numbers were lower than required.(4)    In contrast, healthcare providers were fatigued with a high load of patients; this impaired the quality of the service delivered. Sometimes, it was felt that ART nurses did not have empathy and rushed when writing prescriptions, without addressing patients’ concerns about their treatment. This impaired the ART nurse- patient relationship and patients did not enjoy coming to the clinics.(10) | (1, 4, 10, 18) |
|  | Unable to offer solutions to patients social problems |  | Nevertheless, they were often confronted with a range of ‘social issues’, which posed challenges to their clients’ adherence to the rules but which they were rarely in a position to address. By just repeating that it is the client’s responsibility to follow ‘the rules’, counsellors can divert attention away from the fact that they often cannot help clients address socio-economic barriers to following the rules | (1, 21) |
|  | Service provider burnout |  | Service providers in our study discussed burnout and demotivation as a result of staff shortages, unrealistic workloads and lack of supervision and training.(1)  Additionally, all the clinics were handling a large number of clients and many counsellors complained of feeling burnt out. In the rural hospital, Joyce was in charge of all ongoing counselling. On each clinic day (twice a week) she saw between 10 and 20 clients in sessions, which were to encompass clinical consultation, counselling, adherence monitoring and drug dispensing. In these sessions, Joyce concentrated on giving the clients easily repeatable instructions and not much else. (21) | (1, 21) |
|  | Staff are under pressure to have good treatment outcomes |  | While clients may depend on their counsellor as a patron, counsellors also depend on clients for their own job success. Pushing clients to follow the rules, and refusing clients, who will prove ‘difficult’, helps the counsellors ensure that ‘the data’ will show that they are doing a good job….. The clinics all gather data on enrolment, adherence and mortality rates, which are used in reports to their donors. The donors, in turn, use the numbers to demonstrate how they are saving lives. Especially in the Kampala NGO, these statistics were used as performance indicators for counsellors and community workers. When the data showed setbacks in mortality and retention rates, the management blamed the counsellors for not doing their job well enough (21) | (5, 21) |

1. Layer EH, Kennedy CE, Beckham SW, Mbwambo JK, Likindikoki S, Davis WW, et al. Multi-level factors affecting entry into and engagement in the HIV continuum of care in Iringa, Tanzania. PLoS One. 2014;9(8):e104961.

2. Ware NC, Wyatt MA, Geng EH, Kaaya SF, Agbaji OO, Muyindike WR, et al. Toward an understanding of disengagement from HIV treatment and care in sub-Saharan Africa: a qualitative study. PLoS Med. 2013;10(1):e1001369; discussion e.

3. Bogart LM, Chetty S, Giddy J, Sypek A, Sticklor L, Walensky RP, et al. Barriers to care among people living with HIV in South Africa: contrasts between patient and healthcare provider perspectives. AIDS Care. 2013;25(7):843-53.

4. Elwell K. Social and Structural Factors Affecting Women’s Participation in prevention of mother to child transmission(PMTCT) programs in Malawi. Antrhopology. 2015;Doctor of Philosophy:210.

5. Braga B, M., T. “Death is Destiny”: Sovereign Decisions and the Lived Experience of HIV/AIDS and Biomedical Treatment in Central Mozambique: University at Buffalo, State University of New York; 2013.

6. Katz IT, Bogart LM, Cloete C, Crankshaw TL, Giddy J, Govender T, et al. Understanding HIV-infected patients' experiences with PEPFAR-associated transitions at a Centre of Excellence in KwaZulu Natal, South Africa: a qualitative study. AIDS Care. 2015;27(10):1298-303.

7. Layer EH, Brahmbhatt H, Beckham SW, Ntogwisangu J, Mwampashi A, Davis WW, et al. "I pray that they accept me without scolding:" experiences with disengagement and re-engagement in HIV care and treatment services in Tanzania. AIDS Patient Care STDS. 2014;28(9):483-8.

8. Campbell C, Scott K, Skovdal M, Madanhire C, Nyamukapa C, Gregson S. A good patient? How notions of 'a good patient' affect patient-nurse relationships and ART adherence in Zimbabwe. BMC Infect Dis. 2015;15:404.

9. Gourlay AW, A.; Birdthisle, I.; Mshana, G.; Michael, D.; Urassa, M. ‘‘It Is Like That, We Didn’t Understand Each Other’’: Exploring the Influence of Patient-Provider Interactions on Prevention of Mother-To-Child Transmission of HIV Service Use in Rural Tanzania. PLoS One. 2014;9(9).

10. Thorne C, Bezabhe WM, Chalmers L, Bereznicki LR, Peterson GM, Bimirew MA, et al. Barriers and Facilitators of Adherence to Antiretroviral Drug Therapy and Retention in Care among Adult HIV-Positive Patients: A Qualitative Study from Ethiopia. PLoS ONE. 2014;9(5).

11. Kennedy CE, Baral SD, Fielding-Miller R, Adams D, Dludlu P, Sithole B, et al. "They are human beings, they are Swazi": intersecting stigmas and the positive health, dignity and prevention needs of HIV-positive men who have sex with men in Swaziland. J Int AIDS Soc. 2013;16 Suppl 3:18749.

12. Siu GE, Seeley J, Wight D. Dividuality, masculine respectability and reputation: how masculinity affects men's uptake of HIV treatment in rural eastern Uganda. Soc Sci Med. 2013;89:45-52.

13. Guise A, Rhodes T, Ndimbii J, Ayon S, Nnaji O. Access to HIV treatment and care for people who inject drugs in Kenya: a short report. AIDS Care. 2016;28(12):1595-9.

14. Busza J, Dauya E, Bandason T, Mujuru H, Ferrand RA. "I don't want financial support but verbal support." How do caregivers manage children's access to and retention in HIV care in urban Zimbabwe? J Int AIDS Soc. 2014;17:18839.

15. McMahon SA, Kennedy CE, Winch PJ, Kombe M, Killewo J, Kilewo C. Stigma, Facility Constraints, and Personal Disbelief: Why Women Disengage from HIV Care During and After Pregnancy in Morogoro Region, Tanzania. AIDS and Behavior. 2016;21(1):317-29.

16. Naik R. Linkage to care following

home-based HIV counseling and testing: a mixed methods study in rural South Africa: University of Boston; 2013.

17. Axelsson JM, Hallager S, Barfod TS. Antiretroviral therapy adherence strategies used by patients of a large HIV clinic in Lesotho. J Health Popul Nutr. 2015;33:10.

18. Saleem HT, Mushi D, Hassan S, Bruce RD, Cooke A, Mbwambo J, et al. "Can't you initiate me here?": Challenges to timely initiation on antiretroviral therapy among methadone clients in Dar es Salaam, Tanzania. Int J Drug Policy. 2016;30:59-65.

19. Nakanwagi S, Matovu JK, Kintu BN, Kaharuza F, Wanyenze RK. Facilitators and Barriers to Linkage to HIV Care among Female Sex Workers Receiving HIV Testing Services at a Community-Based Organization in Periurban Uganda: A Qualitative Study. J Sex Transm Dis. 2016;2016:7673014.

20. Jones C. Between State and Sickness: The Social Experience of HIV/AIDS illness management and treatment in Grahamstown, South Africa [Dissertation]: Graduate School-New Brunswick

Rutgers, The State University of New Jersey; 2014.

21. Rasmussen LM. Counselling clients to follow 'the rules' of safe sex and ARV treatment. Cult Health Sex. 2013;15 Suppl 4:S537-52.
