# Supplementary material for: Being HIV positive and staying on antiretroviral therapy in Africa: A qualitative systematic review and theoretical model
Source: PLoS One. 2019 Jan 10;14(1):e0210408. doi: 10.1371/journal.pone.0210408 (PMC6328200; doi:10.1371/journal.pone.0210408)
Supplement: S7 Evidence Annex — (DOCX) [file pone.0210408.s013.docx]

| **Theme 7: The new normal requires daily drugs** | | | | |
| --- | --- | --- | --- | --- |
| **Sub-themes** | **Codes** | **Sub-code** | **Illustrative quote(s)** | **Supporting papers** |
| **Accepting HIV is difficult** | Initial acceptance of diagnosis is often related to perceived risk |  | “First, the illness is often associated with immoral behaviour. One women (Elisabeth) became ill, suspected that it was TB, but was not surprised when she tested positive for HIV, given that her estranged husband was seriously ill with all the symptoms normally ascribed to HIV. However, it is especially difficult for her to reconcile with the fact that HIV was supposed to be happening to others, mostly ‘immoral people’, given her strong religious convictions…”(1)  “Clients' reasons for disbelief of their results were often related to views about their behavior and perceived risk…..Given the prevalence of HIV in the country and their immediate environment and experiences, participants were often expecting a positive diagnosis. One respondent (Susan) for example indicated that her lifestyle – including partying and drinking – left her expecting the negative news. (2) | (1, 2) |
|  | An HIV diagnosis is often met with shock and disbelief |  | “Two other participants (Brenda and Sarah) also indicated that the unfortunate news of a HIV- positive diagnosis was first met with disbelief.”(1)  Participants typically described their initial diagnosis as invoking feelings of shock and fear, including thoughts of impending death (3) | (1-7) |
|  | Denial of HIV status is a common initial response and many want to re-test |  | One woman described how her good health coupled with the lack of technical testing equipment fueled a sense of incredulity about her status. (4)  “Both pregnant and postpartum women reported feeling uncomfortable initiating ART because they did not believe the test result and wanted to get a second test (often at a different facility) before initiating ART.”(5)  Participants discussed the initial shock and denial that the HIV diagnosis engendered, due to the fear of others finding out and discriminating against them. Denial was seen by many as one of the top issues faced by people newly-diagnosed with HIV.(8) | (2, 4, 5, 8-10) |
|  | Coping with the diagnosis | After diagnosis people have to grapple with the possible loss of identity and future aspirations | “Related, the perceived possibility of transmitting HIV to an unborn child or dying because of a complicated pregnancy or birth disrupted the idea of future motherhood for some participants in their reproductive years, which is an important aspect of female identity. These practical barriers have important symbolic significance for the lives and resulting identity of these women.” (1)  Several other men reported feeling diminished by having acquired HIV. In their narratives, there was a great sense of loss…..In the interviews, there was a great deal of angst among men when discussing issues of childbearing and father- hood. Men who had no biological children showed strong emotions of sadness and regret when discussing this topic. For them, having HIV meant that they would not leave anyone behind to perpetuate their family name, proving them to be failures in the eyes of their families. (11) | (1, 11) |
|  |  | Some turn to substance abuse to help them cope with the diagnosis | “Although not commonly mentioned, some clients' difficulty accepting and coping with their newfound status was associated with excessive alcohol use.” (2)  “Consistent with Jeff’s experience, Luxolo highlighted the common use of excessive alcohol as a way of avoiding to confront the reality of the diagnosis” (11) | (2, 11) |
|  |  | Some feel hopeless and depressed after diagnosis | “On the other hand, some participants feared death after testing and lost hope for living; they did not see the need for starting on ART” (10)  “Four women spoke on how they lost hope in life and were less interested in taking ART especially after saving the child” (12) | (2, 10-12) |
|  | Over time, most people learn to accept an HIV diagnosis |  | Since acceptance is a process, even clients who initially had very negative responses to their diagnoses were able to come to terms with it over time. This is clearly illustrated by one client who had suicidal thoughts, but later accepted the situation, and even sought care promptly. (2)  However, most women spoke about how they came to accept their HIV status over time, particularly as they realized that they were not alone in living with HIV. (3)  Both had chosen to delay disclosing her HIV status to most of her family and friends. Her decision seemed to be guided more by a need to personally cope with her diagnosis before sharing it with others, as she had been diagnosed only four months prior to our interview. (13) | (2-4, 9, 10, 13, 14) |
|  | It is hard to initiate ART if you haven’t accepted your HIV diagnosis |  | Others, who reacted with disbelief or denial to their positive test results, put the care continuum on hold for several years .(9)  Some FSWs mentioned that they could not enrol in an HIV clinic since they did not believe their HIV results. Some thought it was just a prick performed hurriedly in the night and wondered how it translated into an HIV positive result so they waited and retested many times (10)  Although they may have felt upset, disturbed, or even struggled with acceptance for a period of time, a common response to the diagnosis among clients who quickly linked to care was an expression of acceptance or at the least, resignation about their HIV status. Thus it seems that having come to terms with an HIV-positive diagnosis is an important factor in facilitating timely linkage to care (2). | (2, 5, 7, 9, 10, 14) |
| **Time and health status may influence motivation to engage in care** | People may need time to prepare to start ART, making same day ART initiation a challenge |  | This may be why we found that in the context of B+, women expressed the need for additional time to prepare. They wanted time to discuss their status with family members, partners, as well as personal time to cope with their status. Also notable were the number of women who stopped and then re-started ART within the first few months of initiation. These women may not have been ready to start in the first place. (7)  Knowing one’s status (by testing) and being asked to start treatment, imposed a heavy burden that many women felt required adequate time and space to prepare and integrate in daily life. (15)  As most of the women were diagnosed to be HIV infected when they were screened at the antenatal clinic they had no time to prepare themselves psychologically and adapt to a new way of life including ART adherence despite the counseling they got. (12)  “Furthermore, women in this study described feeling unprepared or in shock during the counseling session when they were advised on facets of care and treatment.”(4)  Some women reported being too distracted with news of their HIV status to listen to or understand the counselling messages. (5) | (4, 5, 7, 12, 15) |
|  | Health status markedly influences motivation to engage in care and adhere to ART | HIV related illness is a motivator to initiate and maintain art | “Experiencing a decline in health was another reason for starting or restarting. One woman, who had quit three times before due to side effects, started again after feeling sick.” (7)  “An individual’s health was a strong influencing factor in progression through the continuum of care. Those who were visibly sick and had ruled out other causes of disease were most likely to seek HTC services, accept their diagnosis and immediately link to care and treatment.”(6)  Most patients had been confirmed as HIV-positive after suffering a long-term sickness. They had seen the devastating effect of HIV on their bodies and had vivid illnesses and stories. A significant improvement of health witnessed soon after initiating ART heightened trust in the medications.(16) | (6, 7, 16-21) |
|  |  | Feeling healthy reduces motivation to initiate ART | In contrast, participants widely acknowledged that asymptomatic individuals were much less likely to link to a CTC because they either did not believe the HIV test results or did not see the point in receiving care (6)  “For example, items such as food, transport to work, attending to the needs of other family members, or purchasing children's school uniforms are likely to take priority over care seeking. These "more important" items may repeatedly displace care seeking, diminishing its urgency over time, particularly if clients are still feeling well and unhindered by their illness.” (2)  “Motivation was also affected by the fact that most women felt well and were clinically stable and had been put on ART because they were diagnosed during pregnancy.” (12) | (2, 4-6, 12-15) |
|  |  | Improvement of health status allow patients to live normal lives which may impair motivation to continue ART | “Many adolescents discussed their poor health before initiating medication and how their health improved and they were able to resume usual activities after initiation of treatment. The medication gave them not only physical health (such as making them grow or gain weight (section 1, quote 1), but also hope and the ability to lead a ‘‘normal’’ life.” (19)  “When clients’ health improve after taking ART, sexual relations and reproduction may play an important part in their attempts of ‘reinstatement into a social world’ that places great value on marriage and child-bearing” (22)  Peter’s case shows that the biomedical explanation is appealing in the beginning, when it brings a swift recovery from an almost fatal condition. This experience often starts to wear off when the immediate health crisis is overcome and the patient’s health is restored. In the course of recovery, it is argued, the patient turns back into a person again, and many at this stage try to shed, rather than embrace, their HIV-positive identity. Only few had managed to carve out a living as AIDS activists. Most were eager to return to a ‘normal’ life, seeking employment or setting up a small business, remarrying, and having children. Many regarded their HIV identity as impeding this process. (23) | (19, 22, 23) |
| **People need to adapt to the medicalization of life** | Accepting the biomedical rationale helps but is not essential | Some fully accept the biomedical rationale | “Acquiring knowledge about HIV and ART from health workers was a great psychological resource and emotional ‘boost’: it reduced uncertainty and anxiety, provided hope, and gave them an inner strength to carry on. They could start to see the possibility of regaining control over their health and their lives again…” (24)  “Almost all participants viewed the life-long treatment as a second chance at life and were thus very motivated to consciously comply with treatment guidelines. ART was thus cited as a crucial component of the biographical reconstruction after the HIV-positive diagnosis.” (1) | (1, 24, 25) |
|  |  | Others accept ART but maintain their own belief system | “However, the acceptance of alternative labels for his illness did not prevent Reggie from taking ARVs and from carefully adhering to prescriptions about their use.” (26)  “One woman, who stopped because of religious beliefs, also restarted in part due to her religious beliefs because she wanted to combine prayers with ART to protect her unborn child.”(7) | (6, 7, 11, 19, 26, 27) |
|  | Patient need to accept to concept of life long treatment in order to adhere to ART |  | “Phindile, for example, felt that she was not willing to cope with the responsibility of lifelong adherence to ART because medication would, “complicate my life” ”(13)  “An acceptance of the medicalization of their lives was evident: taking the drugs was ingrained into their minds” (28) | (1, 2, 5, 13, 28) |
|  | A lot of changes have to made in people’s lives to incorporate ART |  | “Although physical recovery due to ART meant that some men were able to restore their company and masculinity with other men, they still faced a dilemma finding the right balance between life on ART and the conventional life style of men in the village. For many men, the ‘rules’ of ART, such as the requirement for reduced smoking and alcohol consumption, both of which most men in the village tend to consume, particularly in the company of others in bars, and adherence to strict and fixed time schedules for swallowing the drugs, caused additional and fundamental lifestyle changes.” (20)  “Paulo, and others like him became “patient-citizens” who discipline themselves, and adjust their schedules, their eating habits, their sexuality, their family relationships to the specific demands and challenges posed by AIDS and its treatment, reformulating their lives.” (29) | (11, 15, 17, 20, 29) |
|  | Some learn to adapt HIV/ ART guidelines to suit their needs |  | “Men’s responses overall can be summarized as “abiding by most of the rules, most of the time,” and choosing to interpret them in ways that enabled them to sustain pleasure and existing relationships.”(28) | (24, 28, 30) |
|  | Some form an new identity | Accept a new version of self and create a new identity | “Processes of reduced self-stigmatisation were evident in all the narratives and an important part of a positive ‘adjustment to a new self’. The reconceptualization of HIV as a normal disease and the normalisation of life discussed above helped participants reappraise their identity as a ‘normal’ person. (24)  ART both enabled men to again perform normative ideals of masculinity, and allowed them to re-examine some common norms and develop a different sense of masculinity which they thought was consistent with and safe for their life with HIV. (20) | (1, 2, 11, 20, 24, 31) |
|  |  | Some generate a new advocate/activist identity | However, Dixon’s HIV diagnosis and the acceptance of his status in his community had provided him with a new opportunity related to HIV advocacy and care: he explained that he had become a ‘‘rapid tester’’, ‘‘counselor’’ and a ‘‘community mobiliser’’. (32)  “The participants often pin- pointed this shift toward clinic advocacy to their initiation onto the ART program; they indicated a shift from feeling “afraid” about the diagnosis to feeling hopeful because they “understand now.” This shift, coupled with the role of men in their communities, could point toward a willingness among men to take on advocacy roles.” (25)  Peter is in many ways a model patient-citizen, who educates others and serves as an example. He had integrated scientific information on the drugs and the virus into his lay understanding of his condition and views the drugs as his lifeline – without them he would be dead. He scolds others who are lax in their adherence and gets angry when peo- ple voice their thoughts on stopping the treatment.(23) | (23, 25, 32) |
| **Despite apparent acceptance many still hope for a cure** |  |  | “There is thus a blurred distinction between illness and well-being; an ostensible acceptance combined with a more subdued rejection…Susan and Elisabeth have also mentioned that they wanted to be completely healed from HIV. Susan often asked us when they are going to discover a cure for ‘this illness of mine’, while Elisabeth believes that one day she is actually going to get cured through prayer, as other people in her church have apparently been cured of HIV.” (1)  “As individual morbidity ebbs and surges, as new models of explanation and treatment appear and old ones are discarded, and as phases and situations in life change, so people change their minds, try different approaches, abandon others, and many never give up seeking the one thing that biomedicine cannot offer: a cure.”  “Most children were furthermore convinced that sooner or later a cure for HIV would be found, which certainly contributed to their overall optimism.”(18)  In a separate account, Jeffrey reported a lengthy treatment interruption that was provoked by a group claiming to possess a cure for HIV that required discontinuation of ART. (33) | (1, 18, 23, 33) |

1. Wouters E, De Wet K. Women's experience of HIV as a chronic illness in South Africa: hard-earned lives, biographical disruption and moral career. Sociol Health Illn. 2016;38(4):521-42.

2. Naik R. Linkage to care following

home-based HIV counseling and testing: a mixed methods study in rural South Africa: University of Boston; 2013.

3. Watt MH, Dennis AC, Choi KW, Ciya N, Joska JA, Robertson C, et al. Impact of Sexual Trauma on HIV Care Engagement: Perspectives of Female Patients with Trauma Histories in Cape Town, South Africa. AIDS and Behavior. 2016;21(11):3209-18.

4. McMahon SA, Kennedy CE, Winch PJ, Kombe M, Killewo J, Kilewo C. Stigma, Facility Constraints, and Personal Disbelief: Why Women Disengage from HIV Care During and After Pregnancy in Morogoro Region, Tanzania. AIDS and Behavior. 2016;21(1):317-29.

5. Katirayi L, Namadingo H, Phiri M, Bobrow EA, Ahimbisibwe A, Berhan AY, et al. HIV-positive pregnant and postpartum women's perspectives about Option B+ in Malawi: a qualitative study. Journal of the International AIDS Society. 2016;19(1).

6. Layer EH, Kennedy CE, Beckham SW, Mbwambo JK, Likindikoki S, Davis WW, et al. Multi-level factors affecting entry into and engagement in the HIV continuum of care in Iringa, Tanzania. PLoS One. 2014;9(8):e104961.

7. Kim MH, Zhou A, Mazenga A, Ahmed S, Markham C, Zomba G, et al. Why Did I Stop? Barriers and Facilitators to Uptake and Adherence to ART in Option B+ HIV Care in Lilongwe, Malawi. PLoS One. 2016;11(2):e0149527.

8. Bogart LM, Chetty S, Giddy J, Sypek A, Sticklor L, Walensky RP, et al. Barriers to care among people living with HIV in South Africa: contrasts between patient and healthcare provider perspectives. AIDS Care. 2013;25(7):843-53.

9. Masquillier C, Wouters E, Mortelmans D, van Wyk B. On the road to HIV/AIDS competence in the household: building a health-enabling environment for people living with HIV/AIDS. Int J Environ Res Public Health. 2015;12(3):3264-92.

10. Nakanwagi S, Matovu JK, Kintu BN, Kaharuza F, Wanyenze RK. Facilitators and Barriers to Linkage to HIV Care among Female Sex Workers Receiving HIV Testing Services at a Community-Based Organization in Periurban Uganda: A Qualitative Study. J Sex Transm Dis. 2016;2016:7673014.

11. Sikweyiya YM, Jewkes R, Dunkle K. Impact of HIV on and the constructions of masculinities among HIV-positive men in South Africa: implications for secondary prevention programs. Glob Health Action. 2014;7:24631.

12. Ngarina MP, R.; Kilewo, C.; Beberfeld, G.; Ekstrom, A., M. Reasons for poor adherence to antiretroviral therapy postnatally in HIV-1 infected women treated for their own health: experiences from the Mitra Plus study in Tanzania. BMC Public Health. 2013;13(450):<http://www.biomedcentral.com/1471-2458/13/450>.

13. Jones C. Between State and Sickness: The Social Experience of HIV/AIDS illness management and treatment in Grahamstown, South Africa [Dissertation]: Graduate School-New Brunswick

Rutgers, The State University of New Jersey; 2014.

14. Saleem HT, Mushi D, Hassan S, Bruce RD, Cooke A, Mbwambo J, et al. "Can't you initiate me here?": Challenges to timely initiation on antiretroviral therapy among methadone clients in Dar es Salaam, Tanzania. Int J Drug Policy. 2016;30:59-65.

15. Mbonye M, Seeley J, Nalugya R, Kiwanuka T, Bagiire D, Mugyenyi M, et al. Test and treat: the early experiences in a clinic serving women at high risk of HIV infection in Kampala. AIDS Care. 2016;28 Suppl 3:33-8.

16. Thorne C, Bezabhe WM, Chalmers L, Bereznicki LR, Peterson GM, Bimirew MA, et al. Barriers and Facilitators of Adherence to Antiretroviral Drug Therapy and Retention in Care among Adult HIV-Positive Patients: A Qualitative Study from Ethiopia. PLoS ONE. 2014;9(5).

17. Okoror TA, Falade CO, Olorunlana A, Walker EM, Okareh OT. Exploring the cultural context of HIV stigma on antiretroviral therapy adherence among people living with HIV/AIDS in southwest Nigeria. AIDS Patient Care STDS. 2013;27(1):55-64.

18. Mattes D. “Life is not a rehearsal, it's a performance”: An ethnographic enquiry into the subjectivities of children and adolescents living with antiretroviral treatment in northeastern Tanzania. Children and Youth Services Review. 2014;45:28-37.

19. Mutwa PR, Van Nuil JI, Asiimwe-Kateera B, Kestelyn E, Vyankandondera J, Pool R, et al. Living situation affects adherence to combination antiretroviral therapy in HIV-infected adolescents in Rwanda: a qualitative study. PLoS One. 2013;8(4):e60073.

20. Siu GE, Wight D, Seeley J. 'Dented' and 'resuscitated' masculinities: the impact of HIV diagnosis and/or enrolment on antiretroviral treatment on masculine identities in rural eastern Uganda. SAHARA J. 2014;11:211-21.

21. Mburu GR, M.; Siu, G.; Bitira, D.; Skovdal, M.; Holland, P. Intersectionality of HIV stigma and masculinity in eastern Uganda: implications for involving men in HIV programmes. BMC Public Health. 2014;14(1061):<http://www.biomedcentral.com/1471-2458/14/1061>.

22. Rasmussen LM. Counselling clients to follow 'the rules' of safe sex and ARV treatment. Cult Health Sex. 2013;15 Suppl 4:S537-52.

23. Beckmann N. Responding to medical crises: AIDS treatment, responsibilisation and the logic of choice. Anthropol Med. 2013;20(2):160-74.

24. Russell S, Martin F, Zalwango F, Namukwaya S, Nalugya R, Muhumuza R, et al. Finding Meaning: HIV Self-Management and Wellbeing among People Taking Antiretroviral Therapy in Uganda. PLoS One. 2016;11(1):e0147896.

25. Zissette S, Watt MH, Prose NS, Mntambo N, Moshabela M. "If you don't take a stand for your life, who will help you?": Men's engagement in HIV care in KwaZulu-Natal, South Africa. Psychol Men Masc. 2016;17(3):265-73.

26. Niehaus I. Treatment literacy, therapeutic efficacy, and antiretroviral drugs: notes from Bushbuckridge, South Africa. Med Anthropol. 2014;33(4):351-66.

27. Asgary R, Antony S, Grigoryan Z, Aronson J. Community perception, misconception, and discord regarding prevention and treatment of infection with human immunodeficiency virus in Addis Ababa, Ethiopia. Am J Trop Med Hyg. 2014;90(1):153-9.

28. Russell S, Namukwaya S, Zalwango F, Seeley J. The Framing and Fashioning of Therapeutic Citizenship Among People Living With HIV Taking Antiretroviral Therapy in Uganda. Qual Health Res. 2016;26(11):1447-58.

29. Braga B, M., T. “Death is Destiny”: Sovereign Decisions and the Lived Experience of HIV/AIDS and Biomedical Treatment in Central Mozambique: University at Buffalo, State University of New York; 2013.

30. Axelsson JM, Hallager S, Barfod TS. Antiretroviral therapy adherence strategies used by patients of a large HIV clinic in Lesotho. J Health Popul Nutr. 2015;33:10.

31. Assefa YL, L.; Wouters, E.; Rasshaert, F.; Peeters K.; Van Damme, W.; . How to improve patient retention in an antiretroviral treatment program in Ethiopia: a mixed-methods study. BMC Health Services Research. 2014;14(45):<http://www.biomedcentral.com/1472-6963/14/45>.

32. Yoshida K, Hanass-Hancock J, Nixon S, Bond V. Using intersectionality to explore experiences of disability and HIV among women and men in Zambia. Disability and Rehabilitation. 2014;36(25):2161-8.

33. Mendelsohn JB, Rhodes T, Spiegel P, Schilperoord M, Burton JW, Balasundaram S, et al. Bounded agency in humanitarian settings: a qualitative study of adherence to antiretroviral therapy among refugees situated in Kenya and Malaysia. Soc Sci Med. 2014;120:387-95.
