# Supplementary material for: Being HIV positive and staying on antiretroviral therapy in Africa: A qualitative systematic review and theoretical model
Source: PLoS One. 2019 Jan 10;14(1):e0210408. doi: 10.1371/journal.pone.0210408 (PMC6328200; doi:10.1371/journal.pone.0210408)
Supplement: S8 Evidence Annex — (DOCX) [file pone.0210408.s014.docx]

| **Theme 8: Personal motivations and support helps** | | | | | |
| --- | --- | --- | --- | --- | --- |
| Sub-themes | Codes | Sub-code | Illustrative quote(s) | | Supporting papers |
| **People vary in how they respond to life’s challenges and have variable self-efficacy** |  |  | “For some clients, motivation to seek care may have been hampered by certain practical realities, whereas for others, such barriers were easily overcome. A combination of the "right" factors, different for each person, is needed to ultimately drive timely linkage.”(1)  “In our study, clients for whom awareness of status was enough to motivate linkage, who recognized the importance of seeking help, and who expressed that nothing could prevent them from seeking care, clearly demonstrated a strong internal locus of control. Meanwhile, the locus of control for those who had difficulty accepting their status and who focused on potential negative outcomes was external.”(1)  “At times, he would physically abuse her when she came home from the clinic, presuming she was unfaithful during her times away from home, but she described a stubborn dedication to continue seeking medical treatment”(2)  “The majority of the participants’ sense of self-efficacy and their successful self-management is unlikely to be typical of all PLWH in this setting. They had tested, were adhering to ART, and although the majority were cautious about disclosure, they were open enough about their status to be willing to participate in the research. Not all PLWH in settings like this can self- manage effectively on ART or achieve the levels of wellbeing evidenced here” (3) | | (1-3) |
| **Familial responsibilities motivate people to stay on ART** | Protecting and caring for children and family is a motivator | Motivation to have an HIV negative child | Some female patients in this study indicated that they adhered to their treatment because they wanted to protect their unborn child from becoming infected. (4)  For the HIV-positive mothers in this study, consuming ARVs was described as a strategy to protect children from the HIV virus and nurture them well into their childhood years. (5) | | (2, 4-7) |
|  |  | Having a family or children to support | “Patients’ commitment to raise and educate their children facilitated medication taking. Patients with HIV were at the peak of their reproductive lives…They fear passing away with AIDS, leaving their children as orphans.”(8)  For some women, the concept of motherhood was a source of resilience and helped them stick with HIV medications. At 32 years and postpartum, Nomsa struggled to find clothing and food for her children, but continually reminded herself that treatment was an essential part of being able to care for her children. (2)  Some men drew on the social construct of male caring roles to seek HIV treatment. They argued that treatment extends life, enabling a man to fulfil his roles as a father and husband. (9) | | (2, 8-12) |
| **Supportive environments and structures help HIV positive people cope with HIV and ART** | Support helps people cope with HIV, rebuild self-esteem and offsets the effects of stigma |  | | Good social relationships and a sense of connection are important sources of wellbeing in their own right, and can also enhance psychological coping at times of stress which sustains subjective wellbeing, for example after the onset of serious illness. (3)  Social support appeared to be a buffer against some forms of stigma, particularly internalized stigma. Though it is not uncommon for methadone clients to lack social support as illustrated above, some of the methadone clients, both men and women, interviewed described that the support of family allowed them to accept their HIV status and motivated them to take care of their health (13)  “Being accepted and supported by significant others was valued by and crucial in empowering men to move from the diminished masculine position and reconfigure another masculine identity which allowed them to live positively with HIV.”(14) | (3, 10, 13, 14) |
|  | Support from those who are close to you - partners, family, friends or teachers can have a substantial impact |  | | “A number of positive narratives of disclosure emerged from the analysis, demonstrating that many partners accepted an intimate partner’s HIV status. Some who disclosed reported an increased ability to engage in HIV care, and a sense of personal empowerment that led them to encourage their partners and others to test, engage in care, and initiate ART. Furthermore, successful disclosure within intimate partnerships often led to engagement in risk-reduction strategies.” (15)  “Receipt of support from spouses and partners seemed particularly helpful.”(1)  “Emotional support from one or two close family members, usually the person(s) to whom the participant had first disclosed, was also important and several participants stated that one particular person “was the reason I am still here”” (3)  “Some teachers reported having taken it upon themselves to provide emotional support and counseling to individual students”(16) | (1, 3, 11, 13, 15-18) |
|  | Some look to spirituality, allopathic healers or organized religion for support |  | | “Praying emerged as another important coping strategy. Respondents reported praying to God to give them the strength to persevere through challenges, and achieve their life aspirations.” (19)  “Spiritual and social resources helped psychological coping and wellbeing. In a context where religiosity is widespread many participants felt God was looking over them and would take on the responsibility, and the stress, of any uncertainty about their health and when they might die. God as a supporter and protector helped sustain psychological wellbeing, despite adversity. (3)  “Traditional healers expressed a central role for themselves in the provision of care for PLHIV – to give and restore patients’ hope for life.”(20) | (3, 19-22) |
|  | Supportive health services and support from other HIV positive people helps people cope with the diagnosis and long term treatment | HIV specific health services often create a nurturing environment | | “The participants also described connecting with other patients in the HIV clinic waiting area to expand their support networks. The researchers’ direct observations noticed this behavior, because patients in the waiting area frequently spoke with one another across gender boundaries.” (11)  “The second experience, frequently and powerfully expressed in the narratives, was the importance of “seeing and being with others who had HIV and looked well,” identifying with these others’ positive experiences. Seeing others was also motivational because it helped them see they were not alone.” (23) | (10, 11, 22-26) |
|  |  | HIV counselling can help patients reframe HIV and cope with diagnosis | | “Health workers had helped processes of adjustment by giving participants a new language for thinking and speaking about HIV, helping participants reconceptualise their condition as ‘*normal*’, *like many other diseases*, and also as one of many causes of death: *you can die from many other diseases and many other things; death comes to us all, so how is HIV any different?* HIV was also reconceptualised as a normal disease by referring to its wide prevalence across the community. From the time of diagnosis, health workers had told participants *you are not alone, look around you*. All the participants drew on this language, using the phrases *I am not the only one, I share this problem with many others.”* (3) | (1, 3, 12, 13, 23, 27, 28) |
|  |  | Respectful and supportive healthcare workers can make a difference | | “Positive relationships with health workers were a critically important resource for the participants, especially in the first months following diagnosis and starting treatment. A powerful theme in the narratives was participants’ enthusiasm for the caring and respectful approach of health workers, and the good relationships they had built with them.” (3)  “Developing trust and mutual respect between healthcare workers and patients encouraged patients to continue coming to the clinic and to openly discuss confusion, adherence problems and concerns. Patients spoke frequently of the importance of networks of support available through the clinic.”(10) | (3, 10, 15) |
|  |  | HIV positive people often support each other | | “People spoke passionately of their HIV support group as central to their ability to cope with life as an HIV-positive person and ART adherent. Support groups provided people with an opportunity to speak openly with others in the same situation. They offered a vital connection to others and a place to share problems and gain confidence to cope with challenges” (10)  “Another facilitator of adherence was to have an HIV- positive partner, relative or friend to share the experience with or get reminders and support from.” (17) | (3, 10, 12, 17, 22) |
|  | Community health workers and community organizations offer a variety of support services and often help patients re-engage in care |  | | “An important facilitator to starting or restarting was encouragement from CHWs” (7)  “Home based care providers (HBCs), community volunteers who serve as a link between the health system and community by visiting PLHIV in their homes and following up after missed appointments, were present in two CTC facilities in this study. In these facilities, HBCs were mentioned as a very effective system for following up with patients who did not attend their CTC visits since they lived and worked at the village level. Service providers at the only CTC facility with HBCs said that they rarely lost clients to follow-up due to the strong support provided by HBCs.” (22) | (7, 10, 22, 29) |
|  | Financial support and food parcels can help alleviate economic challenges however dependency may have unexpectedly negative results |  | | “Food aid, distributed by local NGOs to families with HIV positive members, served to help overcome material, symbolic and relational barriers to treatment. On a material level, increasing the amount of food available in the home helped ensure families could access adequate nutrition. Having enough food to go around for all members of the family ensured that PLWHA could eat the necessary meals to accompany each dose of antiretroviral pills. Access to food for families headed by grandparents was a vital step in enabling children on ART to receive optimal treatment and nutritional support.  Informants reported that people became more open to being tested as a way to access food aid…” (10)  “Some PLWHA who relied on social assistance grants began to modify their adherence to ART because improved health would render them ineligible for the grant, leaving them unable to meet their basic needs. The manipulation of this system has created adverse and unanticipated responses to treatment as many patients are tempted to compromise their health, secured through adherence to ART, in order to meet their basic needs through the grant’s financial assistance.” (30)  Some of the patients, being dependent on the food supply from ART clinics, left their pill bottles with the nurses when they did not receive their food ration from the clinic. (8) | (4, 8-10, 30) |
| **All forms of support; practical, emotional, or financial, can help HIV positive people cope with HIV and ART** |  |  | | “Participants reported receiving emotional support from a variety of sources. One MSM said he went to his pastor for support, while another derived comfort from religion but had not disclosed or discussed his life with his church. Only one participant mentioned going to formal counselling services, saying he and his partner saw a private counsellor who knew they were gay. However, most received support from partners, friends or family to whom they had disclosed either their HIV status or their sexual identity.” (31)  “Across all interviews FSWs who were enrolled in savings and other support groups mentioned advantages of joining these groups. They noted that the members of the support groups like village savings groups started by ROM for sex workers only or mixed with other community members helped them to enrol into care. The members of the savings group reportedly advised them to start on treatment and live longer. Being part of a savings group also created hope that money would be available for food and they would have capital to start their own business and live longer” (12)  The most common form of support given and received by the partners in this study was practical support in the form of physical help when the partner was weak, collecting medication for partners, reminders for treatment doses by partners, going with partners to the clinic, cooking for the partner when they were ill, and helping with household chores. (32) | (8, 12, 13, 18, 31, 32) |

1. Naik R. Linkage to care following

home-based HIV counseling and testing: a mixed methods study in rural South Africa: University of Boston; 2013.

2. Hatcher AM, Stockl H, Christofides N, Woollett N, Pallitto CC, Garcia-Moreno C, et al. Mechanisms linking intimate partner violence and prevention of mother-to-child transmission of HIV: A qualitative study in South Africa. Soc Sci Med. 2016;168:130-9.

3. Russell S, Martin F, Zalwango F, Namukwaya S, Nalugya R, Muhumuza R, et al. Finding Meaning: HIV Self-Management and Wellbeing among People Taking Antiretroviral Therapy in Uganda. PLoS One. 2016;11(1):e0147896.

4. Masquillier C, Wouters E, Mortelmans D, van Wyk B. On the road to HIV/AIDS competence in the household: building a health-enabling environment for people living with HIV/AIDS. Int J Environ Res Public Health. 2015;12(3):3264-92.

5. Elwell K. Social and Structural Factors Affecting Women’s Participation in prevention of mother to child transmission(PMTCT) programs in Malawi. Antrhopology. 2015;Doctor of Philosophy:210.

6. Katirayi L, Namadingo H, Phiri M, Bobrow EA, Ahimbisibwe A, Berhan AY, et al. HIV-positive pregnant and postpartum women's perspectives about Option B+ in Malawi: a qualitative study. Journal of the International AIDS Society. 2016;19(1).

7. Kim MH, Zhou A, Mazenga A, Ahmed S, Markham C, Zomba G, et al. Why Did I Stop? Barriers and Facilitators to Uptake and Adherence to ART in Option B+ HIV Care in Lilongwe, Malawi. PLoS One. 2016;11(2):e0149527.

8. Thorne C, Bezabhe WM, Chalmers L, Bereznicki LR, Peterson GM, Bimirew MA, et al. Barriers and Facilitators of Adherence to Antiretroviral Drug Therapy and Retention in Care among Adult HIV-Positive Patients: A Qualitative Study from Ethiopia. PLoS ONE. 2014;9(5).

9. Siu GE, Seeley J, Wight D. Dividuality, masculine respectability and reputation: how masculinity affects men's uptake of HIV treatment in rural eastern Uganda. Soc Sci Med. 2013;89:45-52.

10. Scott K, Campbell C, Madanhire C, Skovdal M, Nyamukapa C, Gregson S. In what ways do communities support optimal antiretroviral treatment in Zimbabwe? Health Promot Int. 2014;29(4):645-54.

11. Zissette S, Watt MH, Prose NS, Mntambo N, Moshabela M. "If you don't take a stand for your life, who will help you?": Men's engagement in HIV care in KwaZulu-Natal, South Africa. Psychol Men Masc. 2016;17(3):265-73.

12. Nakanwagi S, Matovu JK, Kintu BN, Kaharuza F, Wanyenze RK. Facilitators and Barriers to Linkage to HIV Care among Female Sex Workers Receiving HIV Testing Services at a Community-Based Organization in Periurban Uganda: A Qualitative Study. J Sex Transm Dis. 2016;2016:7673014.

13. Saleem HT, Mushi D, Hassan S, Bruce RD, Cooke A, Mbwambo J, et al. "Can't you initiate me here?": Challenges to timely initiation on antiretroviral therapy among methadone clients in Dar es Salaam, Tanzania. Int J Drug Policy. 2016;30:59-65.

14. Sikweyiya YM, Jewkes R, Dunkle K. Impact of HIV on and the constructions of masculinities among HIV-positive men in South Africa: implications for secondary prevention programs. Glob Health Action. 2014;7:24631.

15. Maeri I, El Ayadi A, Getahun M, Charlebois E, Akatukwasa C, Tumwebaze D, et al. "How can I tell?" Consequences of HIV status disclosure among couples in eastern African communities in the context of an ongoing HIV "test-and-treat" trial. AIDS Care. 2016;28 Suppl 3:59-66.

16. Wolf HTH-F, B., L.; Bukusi, E., B; Kawango, E., A; Cohen, A., R.; Auerswald, C., L. “It is all about the fear of being discriminated

[against]...the person suffering from HIV will not

be accepted”: a qualitative study exploring the

reasons for loss to follow-up among HIV-positive

youth in Kisumu, Kenya. BMC Public Health. 2014;14(1154):<http://www.biomedcentral.com/1471-2458/14/1154>.

17. Axelsson JM, Hallager S, Barfod TS. Antiretroviral therapy adherence strategies used by patients of a large HIV clinic in Lesotho. J Health Popul Nutr. 2015;33:10.

18. Busza J, Dauya E, Bandason T, Mujuru H, Ferrand RA. "I don't want financial support but verbal support." How do caregivers manage children's access to and retention in HIV care in urban Zimbabwe? J Int AIDS Soc. 2014;17:18839.

19. Mutumba M, Bauermeister JA, Musiime V, Byaruhanga J, Francis K, Snow RC, et al. Psychosocial challenges and strategies for coping with HIV among adolescents in Uganda: a qualitative study. AIDS Patient Care STDS. 2015;29(2):86-94.

20. Appelbaum Belisle H, Hennink M, Ordonez CE, John S, Ngubane-Joye E, Hampton J, et al. Concurrent use of traditional medicine and ART: Perspectives of patients, providers and traditional healers in Durban, South Africa. Glob Public Health. 2015;10(1):71-87.

21. Mattes D. “Life is not a rehearsal, it's a performance”: An ethnographic enquiry into the subjectivities of children and adolescents living with antiretroviral treatment in northeastern Tanzania. Children and Youth Services Review. 2014;45:28-37.

22. Layer EH, Kennedy CE, Beckham SW, Mbwambo JK, Likindikoki S, Davis WW, et al. Multi-level factors affecting entry into and engagement in the HIV continuum of care in Iringa, Tanzania. PLoS One. 2014;9(8):e104961.

23. Russell S, Namukwaya S, Zalwango F, Seeley J. The Framing and Fashioning of Therapeutic Citizenship Among People Living With HIV Taking Antiretroviral Therapy in Uganda. Qual Health Res. 2016;26(11):1447-58.

24. Hornschuh S, Laher F, Makongoza M, Tshabalala C, Kuijper LDJ, Dietrich J. Experiences of HIV-Positive Adolescents and Young Adults in Care in Soweto, South Africa. Journal of HIV/AIDS & Social Services. 2014;13(4):420-35.

25. Braga B, M., T. “Death is Destiny”: Sovereign Decisions and the Lived Experience of HIV/AIDS and Biomedical Treatment in Central Mozambique: University at Buffalo, State University of New York; 2013.

26. Katz IT, Bogart LM, Cloete C, Crankshaw TL, Giddy J, Govender T, et al. Understanding HIV-infected patients' experiences with PEPFAR-associated transitions at a Centre of Excellence in KwaZulu Natal, South Africa: a qualitative study. AIDS Care. 2015;27(10):1298-303.

27. Wouters E, De Wet K. Women's experience of HIV as a chronic illness in South Africa: hard-earned lives, biographical disruption and moral career. Sociol Health Illn. 2016;38(4):521-42.

28. Gourlay AW, A.; Birdthisle, I.; Mshana, G.; Michael, D.; Urassa, M. ‘‘It Is Like That, We Didn’t Understand Each Other’’: Exploring the Influence of Patient-Provider Interactions on Prevention of Mother-To-Child Transmission of HIV Service Use in Rural Tanzania. PLoS One. 2014;9(9).

29. Assefa YL, L.; Wouters, E.; Rasshaert, F.; Peeters K.; Van Damme, W.; . How to improve patient retention in an antiretroviral treatment program in Ethiopia: a mixed-methods study. BMC Health Services Research. 2014;14(45):<http://www.biomedcentral.com/1472-6963/14/45>.

30. Jones C. Between State and Sickness: The Social Experience of HIV/AIDS illness management and treatment in Grahamstown, South Africa [Dissertation]: Graduate School-New Brunswick

Rutgers, The State University of New Jersey; 2014.

31. Kennedy CE, Baral SD, Fielding-Miller R, Adams D, Dludlu P, Sithole B, et al. "They are human beings, they are Swazi": intersecting stigmas and the positive health, dignity and prevention needs of HIV-positive men who have sex with men in Swaziland. J Int AIDS Soc. 2013;16 Suppl 3:18749.

32. Bhagwanjee A, Govender K, Reardon C, Johnstone L, George G, Gordon S. Gendered constructions of the impact of HIV and AIDS in the context of the HIV-positive seroconcordant heterosexual relationship. Journal of the International AIDS Society. 2013;16(1).
