# Supplementary material for: Being HIV positive and staying on antiretroviral therapy in Africa: A qualitative systematic review and theoretical model
Source: PLoS One. 2019 Jan 10;14(1):e0210408. doi: 10.1371/journal.pone.0210408 (PMC6328200; doi:10.1371/journal.pone.0210408)
Supplement: S9 Evidence Annex — (DOCX) [file pone.0210408.s015.docx]

| **Theme 9:** | | | | |
| --- | --- | --- | --- | --- |
| Sub-themes | Codes | Sub-code | Illustrative quote(s) | Supporting papers |
| Several external and personal influences combined drive engagment decisions | Complex interplay between several influences drives engagement decisions |  | “Similarly, he tried four times to receive his CD4 test results but was denied every time and told to return on another day. Emmanuel explained that he could not justify wasting his time and money on CTC visits when the quality of services was so low and he was in good health, so he disengaged from care and never returned”.… ..”single barriers and facilitators do not capture the complexity of participant experiences, which often involve multiple competing priorities and challenges”(1)  Examined closely, absences are revealed to be less the simple result of one or another ‘‘reason’’ taken individually, and more the product of complex chains of events. To identify only the initial, ‘‘surface’’ reason is to provide an incomplete, sometimes inaccurate, account. For a full understanding, one must follow the events in the chain. An example is the frequently cited ‘‘problem of transport.’’ Transport problems often gloss a much more complicated set of circumstances.(2)  “Finally, at the nexus of stigma, personal disbelief and facility limitations, multiple ancillary reasons arise: women embrace alternative strategies; forget appointments or pill regimens; cite fears of side effects in themselves or their children; and sense heightened financial, logistical, or familial constraints.”(3)  The linkage to care appears to be affected by a variety of factors, which likely operate in concert. Clients each have a unique set of circumstances, which include potential barriers and facilitating factors, not all of which necessarily hinder or encourage linkage, respectively. In some cases, a combination of various difficult circumstances may ultimately hinder linkage, and similarly a combination of positive circumstances may facilitate linkage.(4) | (1-3, 5) |
|  | For some people one initial transitory event leads to a missed visit and other factors result in sustained disengagement |  | “Another lost her ART when her mother fell sick, and she did not start again because there were no negative consequences to her health after stopping.”(6)  In 2012 however, she disengaged from care due to missing one CTC appointment because she was working away from home. When she returned on another date, she was scolded and yelled at by the providers who refused to give her ART. She tried to return several weeks later but was still denied services as a punishment for missing one appointment and did not want to continue to face service providers who treated her so poorly.(1)  In addition to being complex, many obstacles to missed visits are also transitory. They end, dissipate, or cease to be obstacles as circumstances change over time. Recognizing this raises the question, if initial obstacles no longer block clinic attendance, why don’t patients who miss visits then return? A strong reluctance to return for care after an absence emerged from the qualitative data. (2) | (1, 2, 6, 7) |
| For some there is final event often represents a tipping point and not full picture | Getting sick is often then final factor which drive people to engage or re-engage in care |  | “These descriptions were often coupled with an assurance that at the onset of fever, headaches, or other symptoms indicative of HIV infection in themselves or their babies, women would return to facilities for medicines or treatments.”(3)  They ignored their positive status until they developed new symptoms, became pregnant or became so ill that it impacted on their daily functioning, leading them to return to the health facility for retesting (10)  “One key facilitator prompting linkage to care was severe illness at the time of HIV diagnosis. These clients expressed relief in determining the cause of their illness and were happy to immediately link to a CTC in order to initiate treatment.”(1) | (1, 3, 6, 10, 11) |
|  | Poor clinic services are cited as the final cause for disengagement for many |  | Already faced with a host of logistic and personal constraints, and the likelihood of feeling anxious or fearful of stigma, the prospect of unresponsive or demeaning care is enough to deter even those with a desire to seek care. Particularly disheartening were the findings that many who actually made the attempt at linkage still did not receive the needed services. As some clients so aptly described, these experiences can be extremely deflating. Since care seeking in this environment is so burdensome, overcoming the challenges seems to require several factors to converge: time, fmancial resources, strong will, motivation, and household support. When these factors rarely converge, some may lose motivation entirely.(4)  However, several clients disengaged from care when they could no longer handle being degraded, ridiculed and punished, even though they knew their health would suffer as a consequence.(1) | (1, 2, 4, 6, 7, 12) |

1. Layer EH, Kennedy CE, Beckham SW, Mbwambo JK, Likindikoki S, Davis WW, et al. Multi-level factors affecting entry into and engagement in the HIV continuum of care in Iringa, Tanzania. PLoS One. 2014;9(8):e104961.

2. Ware NC, Wyatt MA, Geng EH, Kaaya SF, Agbaji OO, Muyindike WR, et al. Toward an understanding of disengagement from HIV treatment and care in sub-Saharan Africa: a qualitative study. PLoS Med. 2013;10(1):e1001369; discussion e.

3. McMahon SA, Kennedy CE, Winch PJ, Kombe M, Killewo J, Kilewo C. Stigma, Facility Constraints, and Personal Disbelief: Why Women Disengage from HIV Care During and After Pregnancy in Morogoro Region, Tanzania. AIDS and Behavior. 2016;21(1):317-29.

4. Naik R. Linkage to care following

home-based HIV counseling and testing: a mixed methods study in rural South Africa: University of Boston; 2013.

5. Ngarina MP, R.; Kilewo, C.; Beberfeld, G.; Ekstrom, A., M. Reasons for poor adherence to antiretroviral therapy postnatally in HIV-1 infected women treated for their own health: experiences from the Mitra Plus study in Tanzania. BMC Public Health. 2013;13(450):<http://www.biomedcentral.com/1471-2458/13/450>.

6. Kim MH, Zhou A, Mazenga A, Ahmed S, Markham C, Zomba G, et al. Why Did I Stop? Barriers and Facilitators to Uptake and Adherence to ART in Option B+ HIV Care in Lilongwe, Malawi. PLoS One. 2016;11(2):e0149527.

7. Layer EH, Brahmbhatt H, Beckham SW, Ntogwisangu J, Mwampashi A, Davis WW, et al. "I pray that they accept me without scolding:" experiences with disengagement and re-engagement in HIV care and treatment services in Tanzania. AIDS Patient Care STDS. 2014;28(9):483-8.

8. Hatcher AM, Stockl H, Christofides N, Woollett N, Pallitto CC, Garcia-Moreno C, et al. Mechanisms linking intimate partner violence and prevention of mother-to-child transmission of HIV: A qualitative study in South Africa. Soc Sci Med. 2016;168:130-9.

9. Russell S, Martin F, Zalwango F, Namukwaya S, Nalugya R, Muhumuza R, et al. Finding Meaning: HIV Self-Management and Wellbeing among People Taking Antiretroviral Therapy in Uganda. PLoS One. 2016;11(1):e0147896.

10. Masquillier C, Wouters E, Mortelmans D, van Wyk B. On the road to HIV/AIDS competence in the household: building a health-enabling environment for people living with HIV/AIDS. Int J Environ Res Public Health. 2015;12(3):3264-92.

11. Inzaule SC, Hamers RL, Kityo C, Rinke de Wit TF, Roura M. Long-Term Antiretroviral Treatment Adherence in HIV-Infected Adolescents and Adults in Uganda: A Qualitative Study. PLoS One. 2016;11(11):e0167492.

12. Jones C. Between State and Sickness: The Social Experience of HIV/AIDS illness management and treatment in Grahamstown, South Africa [Dissertation]: Graduate School-New Brunswick

Rutgers, The State University of New Jersey; 2014.
